# Molecular identification and larval morphology of spionid polychaetes (Annelida, Spionidae) from northeastern Japan

**DOI:** 10.3897/zookeys.1015.54387

**Published:** 2021-02-04

**Authors:** Hirokazu Abe, Waka Sato‐Okoshi

**Affiliations:** 1 Department of Biology, Center for Liberal Arts & Sciences, Iwate Medical University, Idaidori 1‐1‐1, Yahaba‐cho, Shiwa‐gun, Iwate 028‐3694, Japan Iwate Medical University Yahaba Japan; 2 Laboratory of Biological Oceanography, Graduate School of Agricultural Science, Tohoku University, Aramaki‐Aza‐Aoba 468‐1, Aoba‐ku, Sendai 980‐8572, Japan Tohoku University Sendai Japan

**Keywords:** Larval identification, meroplankton, molecular identification, phylogeny, planktonic larvae, 16S rRNA, 18S rRNA

## Abstract

Planktonic larvae of spionid polychaetes are among the most common and abundant group in coastal meroplankton worldwide. The present study reports the morphology of spionid larvae collected mainly from coastal waters of northeastern Japan that were identified by the comparison of adult and larval 18S and 16S rRNA gene sequences. The molecular analysis effectively discriminated the species. Adult sequences of 48 species from 14 genera (*Aonides* Claparède, 1864; *Boccardia* Carazzi, 1893; *Boccardiella* Blake & Kudenov, 1978; *Dipolydora* Verrill, 1881; *Laonice* Malmgren, 1867; *Malacoceros* Quatrefages, 1843; *Paraprionospio* Caullery, 1914; *Polydora* Bosc, 1802; *Prionospio* Malmgren, 1867; *Pseudopolydora* Czerniavsky, 1881; *Rhynchospio* Hartman, 1936; *Scolelepis* Blainville, 1828; *Spio* Fabricius, 1785; *Spiophanes* Grube, 1860) and larval sequences of 41 species from 14 genera (*Aonides*; *Boccardia*; *Boccardiella*; *Dipolydora*; *Laonice*; *Paraprionospio*; *Poecilochaetus* Claparède in Ehlers, 1875; *Polydora*; *Prionospio*; *Pseudopolydora*; *Rhynchospio*; *Scolelepis*; *Spio*; *Spiophanes*) of spionid polychaetes were obtained; sequences of 27 of these species matched between adults and larvae. Morphology of the larvae was generally species‐specific, and larvae from the same genus mostly shared morphological features, with some exceptions. Color and number of eyes, overall body shape, and type and arrangement of pigmentation are the most obvious differences between genera or species. The morphological information on spionid larvae provided in this study contributes to species or genus level larval identification of this taxon in the studied area. Identification keys to genera and species of planktonic spionid larvae in northeastern Japan are provided. The preliminary results of the molecular phylogeny of the family Spionidae using 18S and 16S rRNA gene regions are also provided.

## Introduction

Many marine invertebrates including polychaetes pass through a planktonic larval phase during their early life history. As such, planktonic larvae derived from the benthic polychaetes are one of the most numerous and diverse groups of coastal zooplankton (Thorson 1946; Hansen 1999; Omel’yanenko and Kulikova 2002; Blake 2017). Polychaetes are often represented in coastal benthic fauna with high species richness all over the world, and members of this group play a large role in the functioning and food webs of marine ecosystems (Aller 1982; Laffaille et al. 2005; Tomiyama et al. 2005, 2007). However, the field study of larval ecology has been restricted because of difficulties in larval identification, which is largely caused by the radical morphological differences between larval and adult stages, lack of diagnostic key characters of larvae, and lack of information on larval forms of many species as is also the case for other marine invertebrates (Branscomb and Vedder 1982; Shanks 1986; Levin 1990). Extensive efforts to describe planktonic polychaete larvae from coastal waters have been performed for species from European and American waters (Blake 2017, and the references cited therein). However, most of these studies are very limited regarding other areas, although Carrasco (1976) and Wu et al. (1978) described polychaete larvae of many species from Chilean and Chinese waters, respectively. In Japan, the larval development and morphology of some polychaete species have been studied (e.g., Izuka 1912: Nereididae Blainville, 1818; Okada 1930: Syllidae Grube, 1850; Okuda 1946: 9 families; Imajima 1959: Spionidae Grube, 1850; Choe 1960: Eunicidae Berthold, 1827; Yamaji 1966: 16 families; Tokioka 1970: Amphinomidae Lamarck, 1818; Imai 1975, 1982: Eunicidae; Miura and Kajihara 1981: Serpulidae Rafinesque, 1815; Yokoyama 1981, 1996: Spionidae; Sasaki and Brown 1983: Saccocirridae Bobretzky, 1872; Yokouchi 1985, 1988: 27 families; Sato and Tsuchiya 1991: Nereididae; Nishi and Yamasu 1992a, 1992b, 1992c, 1992d: Serpulidae; Yokouchi and Yokouchi 1997: 23 families; Koya et al. 2003: Nereididae; Tosuji and Sato 2006: Nereididae; Kondoh et al. 2017: Spionidae; Kan et al. 2020: Nereididae), and some field ecological investigations of polychaete larvae have been conducted (Yokouchi 1984, 1991; Yokoyama 1990, 1995; Abe et al. 2011, 2014; Kan et al. 2020).

Spionidae is one of the largest taxa of polychaete annelids and currently comprises more than 500 nominal species belonging to approximately 38 genera (Radashevsky 2012; Read and Fauchald 2020, excluding *Poecilochaetus* Claparède in Ehlers 1875 and *Trochochaeta* Levinsen, 1883). Planktonic spionid larvae are often the most common and abundant group in the coastal meroplankton (Anger et al. 1986; Levin 1986; Abe et al. 2011, 2014) because of their high abundance and species richness in coastal zones, high reproductive capacity, and relatively long planktonic stage (Blake 1996; Blake and Arnofsky 1999). As they are often seasonally dominant in coastal zooplankton communities, spionid larvae can play a major role in planktonic trophic dynamics (Martin et al. 1996; Pedersen et al. 2010). They are also reported to constitute a large portion of ballast water species (Carlton and Geller 1993; Carlton 1996). Spionidae includes species that adult inhabit a wide range of substrates and some are symbionts of other invertebrates (Martin and Britayev 1998, 2018; Sato‐Okoshi 1999, 2000; Abe et al. 2019b). Among these, symbionts polydorids (i.e., from the *Polydora* complex or tribe Polydorini, see Radashevsky 2012) are well known as harmful pests in molluscan aquaculture because of their shell boring activities (Blake and Evans 1972; Handley and Bergquist 1997; Simon et al. 2006; Simon and Sato‐Okoshi 2015). Understanding larval dynamics and dispersal is important to prevent the settlement of pest spionid species on the shells of aquaculture mollusks (Sato‐Okoshi et al. 1990; Simon 2015; David et al. 2016). The host/substrate selectivity and settlement mechanism of spionid larvae during their developmental process are also interesting aspects of larval biology. Although various morphological characteristics of larvae including body shape, pigment patterns (placement and number), ciliary organization, and to some extent, chaetae can be generally used to identify the planktonic larvae of spionid species (Blake and Arnofsky 1999), species‐level identification is still difficult because of the lack of information on the larval forms of many species.

The link between larval and adult form has been traditionally achieved by labor‐intensive culturing approaches either through rearing larvae collected from plankton or by spawning adults in the laboratory (Shanks 2001). In recent years, ecological studies on the diversity and distribution of marine planktonic larvae are increasingly depending on molecular methods for accurate taxonomic identification to species level (Andre et al. 1999; Hosoi et al. 2004; Pradillon et al. 2007; Phillips et al. 2008; Heimeier et al. 2010). For future metabarcoding studies, establishment of a comprehensive DNA barcoding library is very useful for rapid identification of planktonic larvae. Meanwhile, the use of molecular methods for identifying planktonic larvae in extensive field surveys handling large numbers of collected samples still requires extensive cost. Since the direct microscopic observation, which allows prompt identification of larvae at low cost, remains the popular technique for distinguishing planktonic larvae, information on larval morphology would be useful for such studies.

The aim of the present study is identification of spionid larvae that dominantly appear among the planktonic polychaete larvae from northeastern Japan (Abe et al. 2011, 2014) by comparing adult and larval gene sequences. The 18S rRNA and 16S rRNA genes was herein used as a marker for species‐level discrimination in Spionidae. Moreover, we report the results of preliminary phylogenetic analysis using these genetic regions and describe the morphologies of spionid larvae with photomicrographs of living specimens.

## Materials and methods

### Sample collection and morphological observation

**Figure 1.**
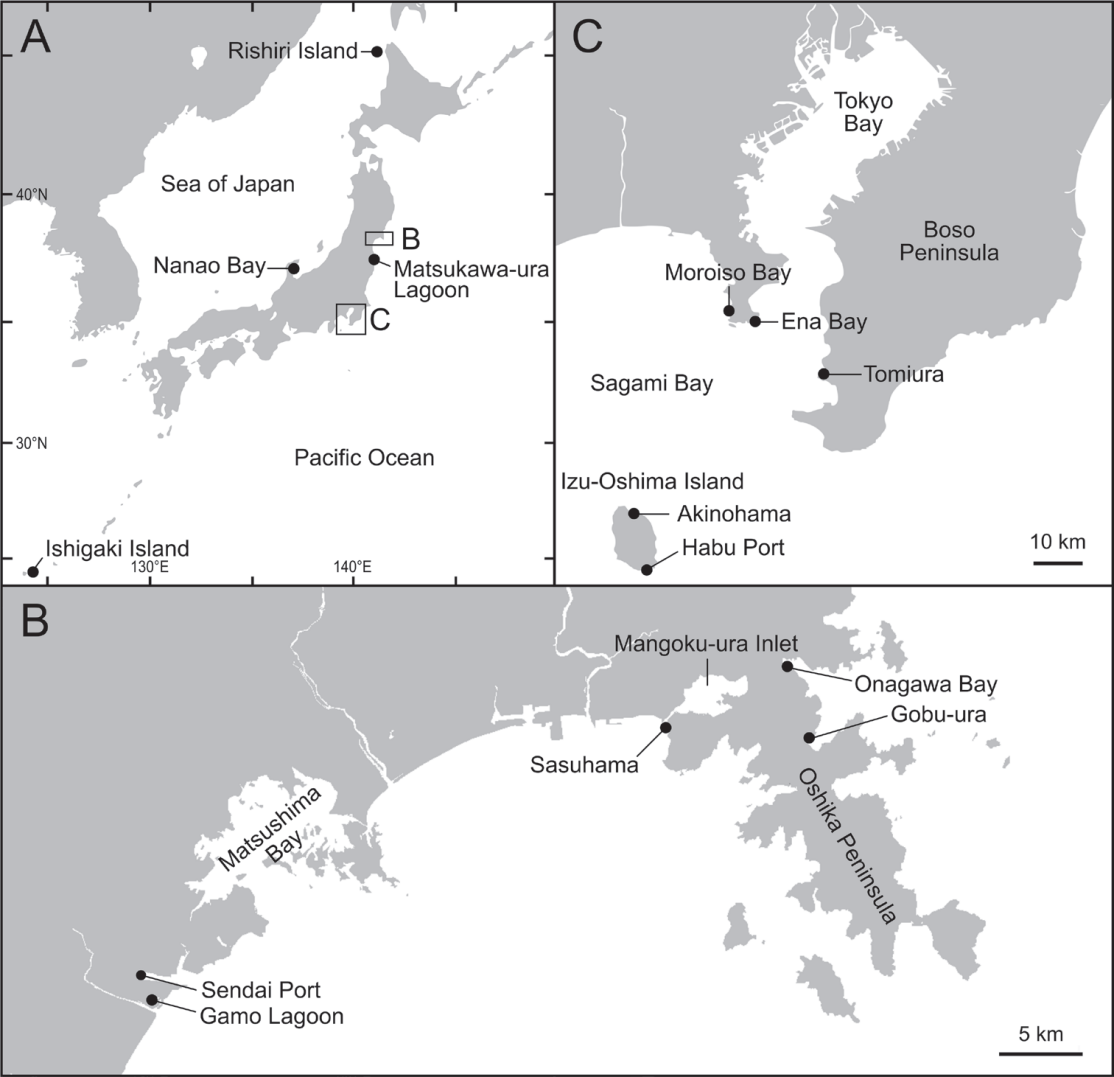
Maps showing sampling locations of the present study.

Planktonic larvae of spionid polychaetes were collected mainly from a coastal station in Onagawa Bay (38°26'15"N, 141°27'42"E; depth: 22 m), but also from Gobu-ura (38°24'01"N, 141°27'59"E), Sasuhama (38°24'22"N, 141°22'08"E), Sendai Port (38°16'22"N, 141°00'01"E), and Gamo Lagoon (38°15'18"N, 141°00'48"E) in Miyagi Prefecture, northeastern Japan, and Tomiura (35°02'20"N, 139°49'16"E) in Boso Peninsula and Habu Port (34°41'09"N, 139°26'16"E) in Izu‐Oshima Island in eastern Japan (Table 1, Fig. 1). Plankton samples were collected in Onagawa Bay once a month, from April 2011 to August 2012, by vertical hauls from the bottom to the surface using a NORPAC net (Motoda 1957) with a mesh size of 110 µm. In the other areas, the plankton samples were collected in 2011–2016 by using a simple plankton net with a mesh size of 100 µm and a mouth diameter of 30 cm. Morphological characteristics of live spionid larvae were observed under stereomicroscopes (Leica, WILD MZ8; Olympus, SZX 16), and light photomicrographs were taken by using digital cameras (Nikon E950, E4500; Olympus DP25, DP73; Sony α6000) attached to the microscope. The larvae were anesthetized with magnesium chloride solution when necessary before the photography. Background, brightness, and contrast of the obtained images were adjusted using GNU Image Manipulation Program (GIMP) 2.10.6 (www.gimp.org). The terms trochophore, metatrochophore, and nectochaeta were defined as larvae with prototroch, clear signs of segmentation, and functional parapodia, respectively according to Rouse (2006).

Adult spionid polychaetes were collected from coastal waters in Shinminato (45°12'27"N, 141°08'09"E) and Numaura (45°06'54.0"N, 141°17'10.0"E) in Rishiri Island, Onagawa Bay, Sasuhama, Matsushima Bay (38°19'54"N, 141°08'44"E), Gamo Lagoon, Ninzaki (37°12'14"N, 136°55'07"E) and Kashima (37°05'13"N, 136°55'35"E) in Nanao Bay, Iwaki (36°55'14"N, 140°51'31"E), Moroiso Bay (35°09'27"N, 139°36'43"E), Ena Bay (35°08'46"N, 139°39'57"E), Tomiura, Akinohama (34°47'12"N, 139°24'32"E) in Izu‐Oshima Island, Ishigaki Island (24°24'01"N, 124°08'30"E), and from a 103‐m depth (by dredging) in Sagami Bay (35°05'N, 139°37'E) in Japan in 2011–2018 (Table 1, Fig. 1). Specimens were fixed in 70% or 99% ethanol. These fixed specimens of adult spionids were observed under stereomicroscopes (Leica, WILD MZ8; Olympus, SZX 16) and a biological light microscope (Nikon, Eclipse80i) and identified based on their morphological characteristics. Following Blake (2006) and Radashevsky et al. (2018), the genera *Poecilochaetus* and *Trochochaeta* were considered as belonging to the family Spionidae.

**Table 1.** Spionid polychaete species collected from Japan in the present and previous studies. Data on life stage (adult/larva), type, and sampling locality, DDBJ/EMBL/GenBank accession numbers, and sequence lengths are given. Accession numbers of gene sequences newly obtained in the present study are highlighted in boldface type.

Classification	Type locality	Sampling locality		Accession no. (length: bp)			
				18S		16S	
		Adult Larvae		Adult Larvae		Adult Larvae	
Nerininae Söderström, 1920							
*Aonides* Claparède, 1864							
Aonides aff. oxycephala (Sars, 1862)	Norway	Onagawa Bay	Onagawa Bay	**LC545853** (1753)	**LC545854** (1753)	**LC595683** (498)	**LC595684** (498)
*Laonice* Malmgren, 1867							
*Laonice* sp. 1	–	Onagawa Bay	Onagawa Bay	**LC545855** (1754)	**LC545856** (1754)	**LC595685** (504)	**LC595686** (504)
*Malacoceros* Quatrefages, 1843							
*Malacoceros indicus* (Fauvel, 1928)	Gulf of Mannar	Ishigaki Island	–	**LC545857** (1757)	–	**LC595687** (510)	–
*Malacoceros* sp.	–	Iwaki	–	**LC545858** (1761)	–	**LC595688** (503)	–
*Paraprionospio* Caullery, 1914							
*Paraprionospio coora* Wilson, 1990	Australia	Onagawa Bay	Onagawa Bay, Sasuhama	**LC545859** (1754)	LC545860 (1754)	**LC595689** (500)	**LC595690** (500)
*Paraprionospio patiens* Yokoyama, 2007	Japan	Ena Bay	–	LC545861 (1709)	–	**LC595691** (500)	–
*Poecilochaetus* Claparède in Ehlers, 1875							
*Poecilochaetus* sp.	–	–	Onagawa Bay	–	LC545862 (1765)	–	**LC595692** (509)
*Prionospio* Malmgren, 1867							
Prionospio aff. cirrifera Wirén, 1883	–	Sasuhama	–	**LC545863** (1752)	–	**LC595693** (497)	–
*Prionospio elongata* Imajima, 1990	Japan	Nanao Bay	–	LC545864 (1752)	–	**LC595694** (466)	–
*Prionospio japonica* Okuda, 1935	Japan	Gamo Lgoon	–	LC545865 (1730)	–	**LC595695** (509)	–
*Prionospio krusadensis* Fauvel, 1929	Gulf of Manaar	Sasuhama	Onagawa Bay	LC545866 (1749)	**LC545867** (1751)	**LC595696** (507)	**LC595697** (507)
*Prionospio lineata* Imajima, 1990	Japan	Nanao Bay	–	LC545868 (1752)	–	**LC595698** (506)	–
*Prionospio membranacea* Imajima, 1990	Japan	Onagawa Bay	Onagawa Bay	**LC545869** (1752)	**LC545870** (1752), **LC545876** (1750)	**LC595699** (505)	**LC595700** (505), LC595701 (505)
Prionospio cf. saccifera Mackie & Hartley, 1990	Hong Kong	Onagawa Bay	–	LC545871 (1753)	–	**LC595702** (497)	–
*Prionospio sexoculata* Augener, 1918	Namibia	Onagawa Bay	–	**LC545872** (1752)	–	**LC595703** (459)	–
*Prionospio variegata* Imajima, 1990	Japan	Akinohama	–	LC545873 (1753)	–	**LC595704** (505)	–
*Prionospio* sp.1	–	–	Onagawa Bay	–	**LC545874** (1752)	–	**LC595705** (500)
*Prionospio* sp.2	–	–	Onagawa Bay	–	**LC545875** (1752)	–	**LC595706** (494)
*Rhynchospio* Hartman, 1936							
Rhynchospio aff. asiatica sensu Radashevsky et al. (2014)	–	Gamo Lagoon, Sasuhama	Onagawa Bay, Gamo Lagoon, Sasuhama	LC545877 (1783)	LC545878 (1783)	**LC595707** (503)	**LC595708** (477)
*Scolelepis* Blainville, 1828							
Scolelepis aff. daphoinos Zhou, Ji & Li, 2009	China	Rishiri Island		LC545879 (1819)		**LC595709** (505)	–
Scolelepis cf. kudenovi Hartmann-Schröder, 1981	Australia		Sasuhama		LC545880 (1819)	–	**LC595710** (505)
*Scolelepis planata* Imajima, 1992	Japan	Ena Bay	–	LC545881 (1816)	–	**LC595711** (501)	–
*Scolelepis texana* Foster, 1971	USA	Nanao Bay, Matsukawa–ura Lagoon	–	**LC545882** (1821)	–	**LC595712** (501)	–
*Scolelepis* sp. 1	–	Onagawa Bay	Onagawa Bay	LC545883 (1819)	LC545884 (1819)	**LC595713** (505)	**LC595714** (505)
*Scolelepis* sp. 2	–	–	Onagawa Bay	–	LC545885 (1820)	–	LC595715 (505)
*Spiophanes* Grube, 1860							
Spiophanes aff. kroyeri Grube, 1860	Greenland Sea	Onagawa Bay	–	LC545886 (1750)	–	**LC595716** (500)	–
*Spiophanes uschakowi* Zachs, 1933	Russia	–	Onagawa Bay	–	**LC545887** (1750)	–	**LC595717** (504)
Spiophanes aff. uschakowi Zachs, 1933	Russia	Sasuhama	Onagawa Bay, Sasuhama	**LC545888** (1750)	LC545889 (1750)	**LC595718** (504)	**LC595719** (504)
*Spiophanes wigleyi* Pettibone, 1962	Georges Bank	Sagami Bay	–	LC545890 (1749)	–	**LC595720** (513)	–
Spioninae Söderström, 1920							
*Boccardia* Carazzi, 1893							
*Boccardia proboscidea* Hartman, 1940	USA	Sasuhama	Sasuhama	LC107607 (1768)^e^	**LC545891** (1768)	**LC595721** (472)	**LC595722** (472)
*Boccardia pseudonatrix* Day, 1961	South Africa	Tomiura	Tomiura	**LC545892** (1745)	LC545893 (1745)	**LC595723** (466)	**LC595724** (466)
*Boccardia* sp. 1	–	–	Onagawa Bay	–	LC545894 (1705)	–	**LC595725** (472)
*Boccardia* sp. 2	–	–	Onagawa Bay, Sashama, Sendai Port	–	**LC545895** (1705)	–	**LC595726** (472)
*Boccardiella* Blake & Kudenov, 1978							
*Boccardiella hamata* (Webster, 1879)	USA	Sasuhama, Gamo Lagoon	Onagawa Bay, Gobu–ura, Sasuhama	LC107608 (1772)^e^	LC545896 (1772)	**LC595727** (472)	**LC595728** (472)
*Dipolydora* Verrill, 1881							
*Dipolydora armata* (Langerhans, 1880)	Madeira	Akinohama	–	LC545897 (1772)	–	**LC595729** (473)	–
*Dipolydora bidentata* (Zachs, 1933)	Russia	Sasuhama	Onagawa Bay	LC107609 (1770)^e^	LC545898 (1770)	**LC595730** (475)	**LC595731** (475)
Dipolydora cf. commensalis (Andrews, 1891)	USA	–	Sasuhama	–	LC545899 (1769)	–	**LC595732** (474)
*Dipolydora giardi* (Mesnil, 1893)	France	Onagawa Bay	Onagawa Bay	**LC545900** (1770)	LC545901 (1766)	**LC595733** (474)	**LC595734** (474)
Dipolydora cf. socialis (Schmarda, 1861)	Chile	Onagawa Bay, Sasuhama	Onagawa Bay	LC545902 (1770)	LC545903 (1770)	**LC595735** (475)	**LC595736** (475)
*Dipolydora* sp.	–	–	Onagawa Bay	–	LC545904 (1770)	–	**LC595737** (476)
*Polydora* Bosc, 1802							
*Polydora aura* Sato-Okoshi, 1998	Japan	Hiroshima Bay	–	AB705409 (1771)^a^	–	LC500931 (473)^g^	–
*Polydora brevipalpa* Zachs, 1933	Russia	Mutsu Bay, Onagawa Bay	Onagawa Bay, Sasuhama	AB705407 (1771)^a^	LC545905 (1766)	**LC595738** (474)	**LC595739** (474)
*Polydora calcarea* (Templeton, 1836)	UK	Kitaibaraki	–	AB705403 (1771)^b^	–	**LC595740** (475)	–
*Polydora cornuta* Bosc, 1802	USA	Sasuhama, Gamo Lagoon	Gamo Lagoon	LC541483 (1742)^g^	LC545906 (1770)	LC541484 (470)^g^	**LC595741** (470)
Polydora cf. glycymerica Radashevsky, 1993	Russia	–	Onagawa Bay, Sendai Port	–	LC545907 (1771)	–	**LC595742** (472)
*Polydora hoplura* Claparède, 1868	Italy	Kitaibaraki	Onagawa Bay, Gobu–ura	LC101841 (1771)^c^	LC545908 (1769)	LC101870 (475)^c^	**LC595743** (475)
*Polydora neocaeca* Williams & Radashevsky, 1999	USA	Hiroshima Bay	–	AB705404 (1771)^b^	–	**LC595744** (471)	–
*Polydora onagawaensis* Teramoto, Sato-Okoshi, Abe, Nishitani & Endo, 2013	Japan	Onagawa Bay	Onagawa Bay	AB691768 (1771)^d^	**LC545909** (1771)	**LC595745** (473)	**LC595746** (473)
Polydora cf. spongicola Berkeley & Berkeley, 1950	Canada	Moroiso Bay	Sasuhama	**LC545910** (1771)	LC545911 (1771)	**LC595747** (475)	**LC595748** (475)
*Polydora websteri* Hartman in Loosanoff & Engle, 1943	USA	Nakatsu tidal flats	–	AB705402 (1771)^b^	–	**LC595749** (468)	–
*Polydora* sp. 1	–	Sasuhama	Onagawa Bay, Sasuhama	**LC545912** (1771)	**LC545913** (1771)	**LC595750** (476)	**LC595751** (476)
*Polydora* sp. 2	–	–	Sasuhama, Gamo Lagoon	–	LC545914 (1771)	–	**LC595752** (4702)
*Polydora* sp. 3	–	–	Onagawa Bay, Sasuhama	–	LC545915 (1771)	–	**LC595753** (473)
*Pseudopolydora* Czerniavsky, 1881							
Pseudopolydora aff. achaeta Radashevsky & Hsieh, 2000	Taiwan	Onagawa Bay	Onagawa Bay	LC019989 (1773)^e^	LC545916 (1773)	**LC595754** (468)	**LC595755** (468)
Pseudopolydora cf. kempi (Southern, 1921)	India	Gamo Lagoon	Gamo Lagoon	LC019990 (1772)^e^	**LC545917** (1772)	**LC595756** (471)	**LC595757** (471)
*Pseudopolydora paucibranchiata* (Okuda, 1937)	Japan	Mangoku–ura Inlet	Onagawa Bay	LC019991 (1784)^e^	**LC545918** (1784)	**LC595758** (455)	**LC595759** (455)
Pseudopolydora cf. reticulata Radashevsky & Hsieh, 2000	Taiwan	Gamo Lagoon	Onagawa Bay, Gamo Lagoon, Sendai Port	LC019988 (1775)^e^	LC545919 (1775)	**LC595760** (470)	**LC595761** (470)
*Pseudopolydora tsubaki* Simon, Sato-Okoshi & Abe, 2017	Japan	Habu Port	Tomiura, Habu Port	AB973929 (1713)^f^	LC545920 (1749)	LC107857 (475)^f^	**LC595762** (425)
*Pseudopolydora ushioni* Simon, Sato-Okoshi & Abe, 2017	Japan	Uranouchi Bay	–	AB973927 (1713)^f^	–	LC107855 (474)^f^	–
*Pseudopolydora* sp.	–	–	Sasuhama	–	**LC545921** (1781)	–	**LC595763** (471)
*Spio* Fabricius, 1785							
*Spio* sp. 1	–	Rishiri Island	Onagawa Bay	**LC545922** (1762)	LC545923 (1762)	**LC595764** (467)	**LC595765** (467)
*Spio* sp. 2	–	Sasuhama, Matsushima Bay	Onagawa Bay, Sasuhama	LC545924 (1760)	LC545925 (1760)	**LC595766** (462)	**LC595767** (462)

^a^: Sato-Okoshi and Abe (2012); ^b^: Sato-Okoshi and Abe (2013); ^c^: Sato-Okoshi et al. (2017); ^d^: Teramoto et al. (2013); ^e^: Abe et al. (2016); ^f^: Simon et al. (2019); ^g^: Abe & Sato-Okoshi (2020).

### DNA analysis and larval identification

**Figure 2.**
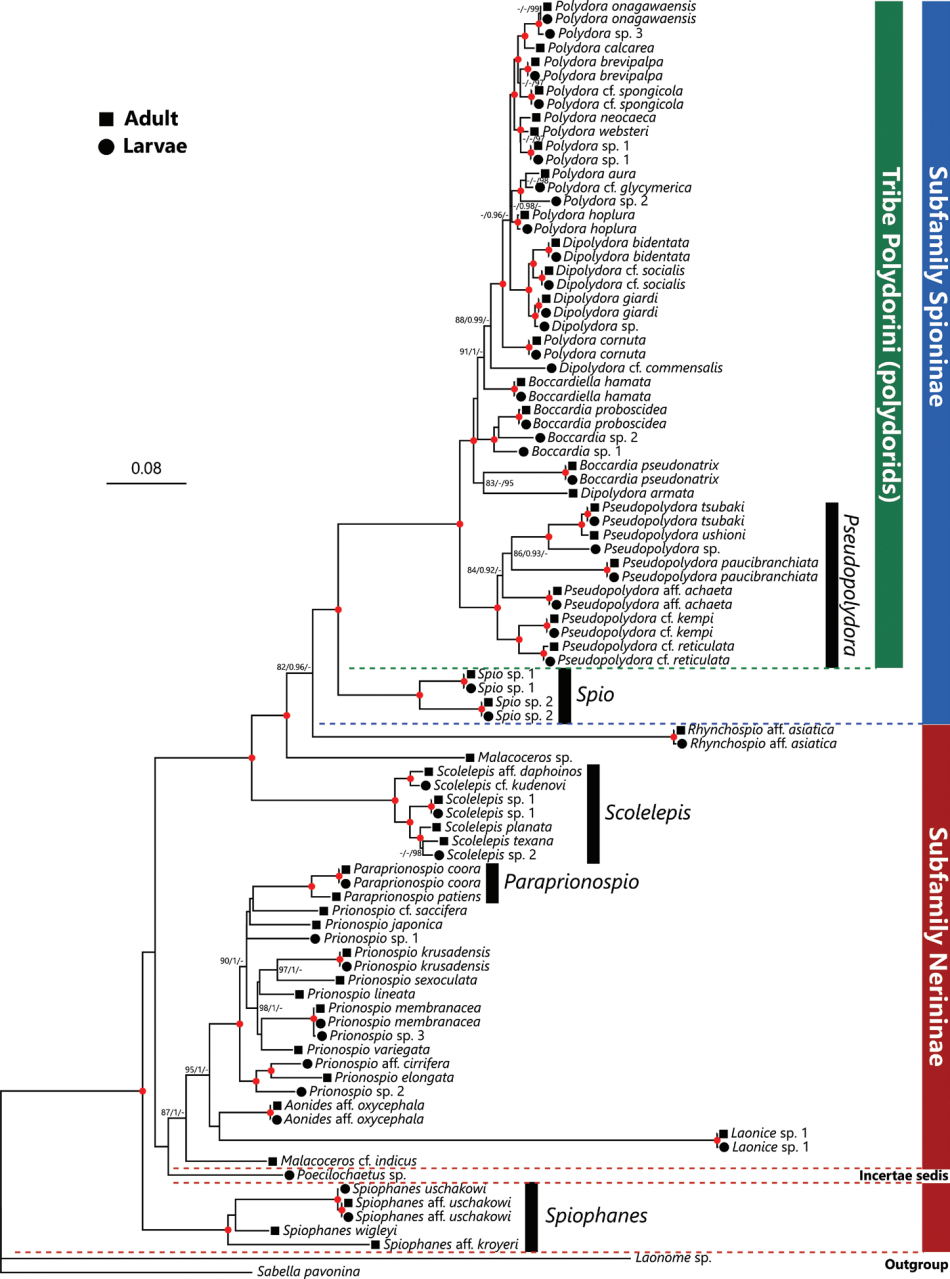
Maximum Likelihood tree inferred from nuclear 18S and mitochondrial 16S rRNA gene sequences of spionids obtained from Japan in the present and previous studies (provided in Table 1). The gene sequences of adult and larval spionid polychaetes are indicated by solid squares and circles in front of each species name, respectively. SH-aLRT/approximate Bayes support/ultrafast bootstrap support values of ≥ 80%, ≥ 0.95, ≥ 95%, respectively are given beside the respective nodes. Nodes with red circles indicate triple high support values of SH-aLRT ≥ 80, approximate Bayes support ≥ 0.95, and ultrafast bootstrap support ≥ 95. The scale bar represents the number of substitutions per site. Sequences of *Laonome* sp. and *Sabella pavonina* Savigny, 1822 obtained from DDBJ/EMBL/GenBank were used for outgroup rooting.

**Figure 3.**
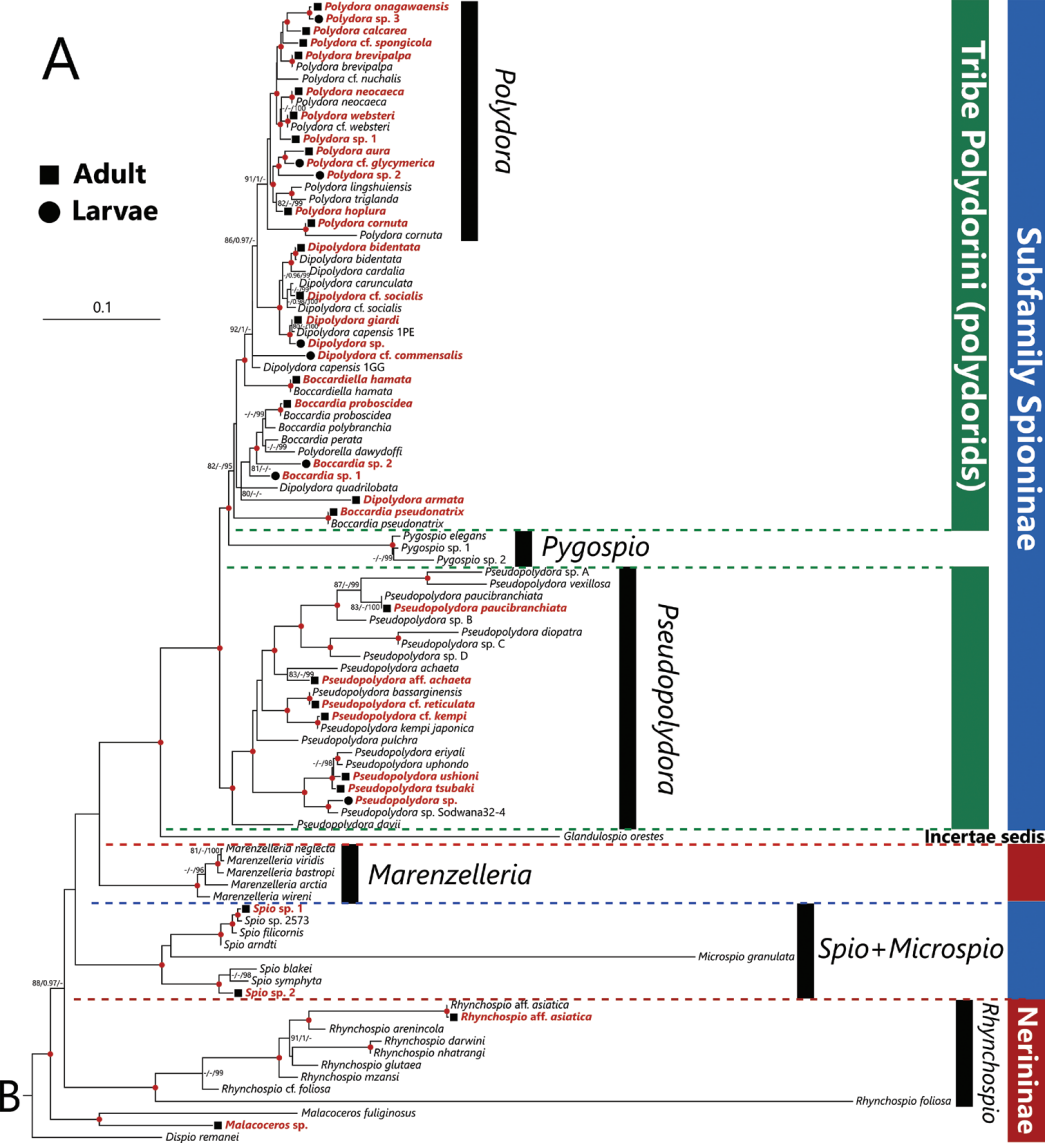
Maximum Likelihood tree inferred from nuclear 18S and mitochondrial 16S rRNA gene sequences of spionid polychaetes obtained from Japan in the present and previous studies (shown in Table 1) and from DDBJ/EMBL/GenBank (shown in Table 2). The tree is divided into two parts **A, B**. The gene sequences obtained in the present study are highlighted by bold and red color and the adult and larval sequences are indicated by solid squares and circles in front of each species name, respectively. SH-aLRT/approximate Bayes support/ultrafast bootstrap support values of ≥80%/≥0.95/≥95%, respectively are given beside the respective nodes. Nodes with red circles indicate triple high support values of SH-aLRT ≥ 80, approximate Bayes support ≥ 0.95, and ultrafast bootstrap support ≥ 95. The scale bar represents the number of substitutions per site. Sequences of *Laonome* sp. and *Sabella pavonina* Savigny, 1822 obtained from GenBank were used for outgroup rooting.

**Figure 4.**
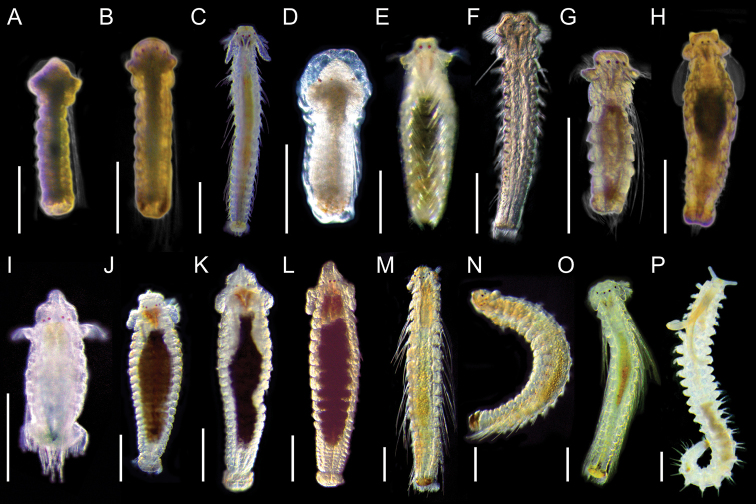
Light micrographs showing morphologies of living spionid larvae of *Aonides*, *Laonice*, *Rhynchospio*, *Scolelepis*, and *Spiophanes***A–C**Aonides cf. oxycephala, dorsal view of early planktonic (**A**), 8‐chaetiger (**B**), and 18‐chaetiger larvae (**C**) **D***Laonice* sp. 1, dorsal view of early planktonic larva **E***Laonice* sp. 2, dorsal view of 12‐chaetiger larva **F***Poecilochaetus* sp., dorsal view of 17‐chaetiger larva **G, H**Rhynchospio aff. asiatica, dorsal view of 6‐chaetiger (**G**) and 12‐chaetiger larvae (**H**) **I**Scolelepis cf. kudenovi, dorsal view of 7‐chaetiger larva **J, K***Scolelepis* sp. 1, dorsal view of 17‐chaetiger (**J**) and 19‐chaetiger larvae (**K**) **L***Scolelepis* sp. 2, dorsal view of 20‐chaetiger larva **M, N***Spiophanes uschakowi*, dorsal (**M**) and lateral view (**N**) of 18‐chaetiger larvae **O, P**Spiophanes aff. uschakowi, dorsal view of 16‐chaetiger larva (**O**) and 27‐chaetiger juvenile (**P**). Scale bars: 300 μm.

Adults and larvae of one or more individuals, respectively, were subjected to DNA analysis. In order to clarify the development links between the different stages, we analyzed the DNA of as many larvae of different stages as possible. Except for *Laonice* sp. 2 (Fig. 4E), all larvae pictured in this paper have been identified by DNA analysis. All individuals were washed by several transfers in sterile filtered (pore size 0.2 μm) seawater and distilled water to remove as much extraneous matter as possible before DNA extraction. Genomic DNA was extracted from live or ethanol‐preserved larval (from the whole body) and adult (from palp or a small piece of tissue) spionid specimens by grinding and heating at 95 °C for 20 min in 50 μl TE buffer (pH 8.0) with 10% Chelex 100 (Bio‐Rad; Richlen and Barber 2005). Undiluted or 10‐fold diluted extracted DNA in TE buffer was used as template for polymerase chain reaction (PCR) depending on the DNA concentration. Partial sequences of nuclear 18S rRNA gene were amplified by PCR according to the methods described by Sato‐Okoshi and Abe (2012, 2013) and Teramoto et al. (2013) using the following primer pairs (Nishitani et al. 2012): 18S‐1F1 (AACCTGGTTKATCCTGCCAG) and 18S‐1R632 (ACTACGAGCTTTTTAACYGCARC), 18S‐2F576 (GGTAATTCCAGCTCYAATRG) and 18S‐2R1209 (AAGTTTYCCCGTGTTGARTC), and 18S‐3F1129 (GCTGAAACTTAAAGRAATTGACGG) and 18S‐R1772 (TCACCTACGGAAACCTTGTTACG). Partial sequences of mitochondrial 16S rRNA gene were amplified by PCR according to the methods described by Abe et al. (2019a) using the 16Sar (CGCCTGTTTATCAAAAACAT) and 16Sbr (CCGGTCTGAACTCAGATCACGT) primer pair (Palumbi et al. 1991). The PCR products were purified using ExoSAP‐IT (Affymetrix, Cleveland, OH, USA) and sequenced by Eurofins Genomics (Tokyo, Japan). The forward and reverse complementary sequences and contigs were assembled using GeneStudio ver. 2.2.0.0 (GeneStudio, Inc. Suwanee, GA, USA). Larval and adult gene sequences obtained in the present study (Table 1) were aligned using the MAFFT online service ver. 7 with the L‐INS‐i algorithm (Katoh et al. 2017) with (Fig. 2) and without (Fig. 3) the sequences of other spionid species available in the DNA Data Bank of Japan (DDBJ), the European Nucleotide Archive (ENA), or GenBank databases (Table 2). The 18S and 16S ribosomal RNA gene sequences of *Sabella pavonina* Savigny, 1822 (DDBJ/EMBL/GenBank ID: U67144 and AY340482) and *Laonome* sp. (KP793139 and KP793138) obtained from DDBJ/ENA/GenBank were used as outgroup taxa. Ambiguously aligned regions of 2 alignments were eliminated by employing Gblocks (Talavera and Castresana 2007) implemented in PhyloSuite v.1.2.2 (Zhang et al. 2020) with the following relaxed settings: minimum number of sequences for a conserved/flank position: half the number of sequences + 1, maximum number of contiguous non-conserved positions: 10, minimum length of a block: 5, and with half of the allowed gap positions. The final lengths of the alignments were 1738 (18S) and 447 (16S) bp for the multiple sequence alignment (MSA) without DDBJ/ENA/GenBank sequences and 1644 (18S) and 410 (16S) bp for MSA with DDBJ/ENA/GenBank sequences. Phylogenetic trees were constructed based on the concatenated sequences of 18S and 16S rRNA gene region by maximum likelihood (ML) analyses performed using IQ-TREE (Nguyen et al. 2015) implemented in PhyloSuite under Edge-linked partition model. The TIM2e+I+G4 and TIM2+F+I+G4 models were selected for the 18S and 16S rRNA gene region, respectively as the best substitution model by ModelFinder (Kalyaanamoorthy et al. 2017) as implemented in IQ-TREE under the Bayesian information criterion (BIC). The robustness of the ML trees was evaluated by the Shimodaira–Hasegawa–like approximate likelihood-ratio test (SH-aLRT) with 5,000 replicates (Guindon et al. 2010), approximate Bayes (aBayes) test (Anisimova et al. 2011), and ultrafast bootstraps (UFBoot) with 5000 replicates (Hoang et al. 2018). SH-aLRT ≥ 80%, aBayes ≥ 0.95, and UFBoot ≥ 95% were defined as robust statistical support. All the sequences newly generated in this study were deposited in the DDBJ/ENA/GenBank nucleotide sequence database under accession numbers LC545853 to LC545925 and LC595683 to LC595767 (Table 1). Part of the sequences used in the present study was reported in the previous studies (see Table 1). The planktonic spionid larvae were identified by comparing larval and adult sequences obtained in the present study and/or by larval morphology.

**Table 2.** Terminal taxa whose sequences were obtained from DDBJ/EMBL/GenBank and herein used in the phylogenetic analyses. Type and collection localities, accession numbers, sequence lengths, and references are shown.

Classification	Type locality	Collection locality	Accession number (Length: bp)		Reference
			18S	16S	
Spionidae					
Nerininae Söderström, 1920					
*Aonidella* López-Jamar, 1989					
Aonidella cf. dayi Maciolek in López-Jamar, 1989	Gulf of Cadiz, Spain	Great Meteor Seamount, NE Atlantic	KF434504 (483)	KF434508 (443)	Meißner et al. (2014)
*Aonides* Claparède, 1864					
*Aonides oxycephala* (Sars, 1862)	Norway	France	MG913226 (1699)	MG878895 (337)	Radashevsky et al. (unpubl.)
*Aonides selvagensis* Brito, Núñez & Riera, 2006	Savage Islands, Portugal	Irving Seamount, NE Atlantic	KF434507 (516)	–	Meißner et al. (2014)
*Aurospio* Maciolek, 1981					
*Aurospio dibranchiata* Maciolek, 1981	Argentine Basin, SW Atlantic	Kaplan, Pacific Mn nodule province	EU340091 (1797)	EU340087 (484)	Mincks et al. (2009)
*Aurospio foodbancsia* Mincks, Dyal, Paterson, Smith & Glover, 2009	Bellingshausen Sea, Antarctica	West Antarctic Peninsula shelf	EU340097 (1765)	EU340078 (552)	Mincks et al. (2009)
*Aurospio* sp. Q	–	India	–	KF459948 (443)	Periasamy et al. (unpubl.)
*Aurospio* sp. R	–	Ross Sea	–	KF713473 (397)	Gallego et al. (2014)
*Aurospio* sp. S	–	Eastern Vema Fracture Zone	MN447187 (844)	MN441726 (409)	Guggolz et al. (2020)
*Aurospio* sp. T	–	Clarion Clipperton Fracture Zone	–	MN441512 (411)	Guggolz et al. (2020)
*Dispio* Hartman, 1951					
*Dispio remanei* Friedrich, 1956	Pacific Ocean, Central America	Brazil	KU900474 (671)	–	Rebelo & Schettini (unpubl.)
*Glandulospio* Meißner, Bick, Guggolz & Götting, 2014					
*Glandulospio orestes* Meißner, Bick, Guggolz & Götting, 2014	Little Meteor Seamount, NE Atlantic	Little Meteor Seamount, NE Atlantic	KF434505 (402)	KF434511 (446)	Meißner et al. (2014)
*Laonice* Malmgren, 1867					
Laonice cf. antarctica Hartman, 1953	Rio Grande do Sul	Antarctic		KX867280 (373)	Brasier et al. (2016)
*Laonice cirrata* (M. Sars, 1851)	Norway	Russia	KM998754 (1744)	–	Radashevsky et al. (unpubl.)
*Laonice norgensis* Sikorski, 2003	Norwegian Sea, North Atlantic	Little Meteor Seamount	KF434506 (514)	KF434512 (454)	Meißner et al. (2014)
Laonice cf. vieitezi López, 2011	Bellingshausen Sea, West Antarctica	Antarctic	–	KX867288 (368)	Brasier et al. (2016)
*Laonice weddellia* Hartman, 1978	Weddell Sea	Antarctic	–	KX867313 (379)	Brasier et al. (2016)
*Lapnice* sp. VR-2006	–	Bohuslän, Sweden	DQ779655 (1705)	DQ779619 (342)	Rousset et al. (2007)
*Laonice* sp. SLM-2008	–	California borderland basins, USA	EU340089 (1784)	EU340088 (546)	Mincks et al. (2009)
*Laonice* sp. A	–	Eastern Vema Fracture Zone	MK507647 (1017)	MK507653 (469)	Guggolz et al. (2019)
*Laonice* sp. B	–	Eastern Vema Fracture Zone	MK507651 (1017)	MK507657 (470)	Guggolz et al. (2019)
*Laonice* sp. C	–	Western Vema–Fracture Zone	MK507650 (1017)	MK507658 (450)	Guggolz et al. (2019)
*Laonice* sp. D	–	Western Vema–Fracture Zone	MK507638 (1017)	MK507723 (475)	Guggolz et al. (2019)
*Laonice* sp. E	–	Western Vema–Fracture Zone	MK507644 (966)	MK507706 (473)	Guggolz et al. (2019)
*Laonice* sp. F	–	Vema Transform Fault	MK507623 (1017)	MK507718 (473)	Guggolz et al. (2019)
*Laonice* sp. G	–	Puerto Rico Trench	MK507624 (1017)	MK507708 (474)	Guggolz et al. (2019)
*Laonice* sp. H	–	Puerto Rico Trench	MK507617 (1017)	MK507720 (474)	Guggolz et al. (2019)
*Malacoceros* Quatrefages, 1843					
*Malacoceros fuliginosus* (Claparède, 1868)	Italy	St. Efflau, France	AY525632 (1765)	–	Struck and Purschke (2005)
		Helgoland, Germany	–	EF431961 (417)	Blank and Bastrop (2009)
*Malacoceros indicus* (Fauvel, 1928)	Gulf of Mannar	Lizard Island, Australia	KP636512 (454)	KP636511 (391)	Meißner and Götting (2015)
*Marenzelleria* Mesnil, 1896					
*Marenzelleria arctia* (Chamberlin, 1920)	Beaufort Sea	Kara Sea, Russia	KJ546264 (1775)	KJ546306 (343)	Radashevsky et al. (2014)
*Marenzelleria bastropi* Bick, 2005	North Carolina,USA	USA	EF446959 (468), EF446967 (577)	EF431963 (419)	Blank and Bastrop (2009)
*Marenzelleria neglecta* Sikorski & Bick, 2004	Germany	Baltic Sea	EF446955 (470), EF446963 (578)	DQ309248 (419)	Bastrop and Blank (2006), Blank and Bastrop (2009)
*Marenzelleria viridis* (Verrill, 1873)	New Jersey, USA	Barlow’s Landing, MA, USA	EU418860 (1810)	–	Struck et al. (2008)
		Ringkøbing Fjord	–	DQ309252 (419)	Bastrop and Blank (2006)
*Marenzelleria wireni* Augener, 1913	Franz Jozef Land, Russia	Spitsbergen, Norway	EF446957 (472), EF446965 (579)	EF431980 (417)	Blank and Bastrop (2009)
*Paraprionospio* Caullery, 1914					
*Paraprionospio cordifolia* Yokoyama, 2007	Wakasa Bay, Japan	Eastern Arabian Sea, India	KT900309 (1655)	–	Rengaiyan and Ingole (2018)
*Paraprionospio cristata* Zhou,Yokoyama & Li, 2008	East China Sea, China	India	KY704338 (520)	–	Vijapure et al. (unpubl.)
*Paraprionospio patiens* Yokoyama, 2007	Osaka Bay, Japan	India	KT900307 (1684)	KY704331 (519)	Rengaiyan and Ingole (2018), Vijapure et al. (unpubl.)
*Paraprionospio* sp. EPK-2019	–	–	MN069511 (588)	–	Kiskaddon et al. (unpubl.)
*Poecilochaetus* Claparède in Ehlers, 1875					
*Poecilochaetus serpens* Allen, 1904	English Channel	Arcachon, France	AY569652 (1833)	AY569680 (463)	Bleidorn et al. (2005)
*Poecilochaetus* sp. VR-2006	–	Banyuls, France	DQ779667 (1710)	DQ779630 (344)	Rousset et al. (2007)
*Poecilochaetus* sp. 18 PB	–	Clarion–Clipperton Fracture Zone	–	MK971106 (419)	Bonifácio et al. (2020)
*Prionospio* Malmgren, 1867					
*Prionospio dubia* Day, 1961	South Africa	Southern New England, MA, USA	EU418859 (1823)	–	Struck et al. (2008)
*Prionospio* sp. A	–	Clarion Clipperton Fracture Zone	–	MN441557 (416)	Guggolz et al. (2020)
*Prionospio* sp. B	–	Eastern Vema Fracture Zone	MN447146 (846)	MN441645 (331)	Guggolz et al. (2020)
*Prionospio* sp. C (as *Prionospio* sp. 29 PB)	–	Clarion–Clipperton Fracture Zone	MK971148 (1677)	MK971035 (422)	Bonifácio et al. (2020)
*Prionospio* sp. D	–	Eastern Vema Fracture Zone	MN447192 (842)	MN441641 (409)	Guggolz et al. (2020)
*Prionospio* sp. E (as *Prionospio ehlersi*)	–	CROZEX	EU340095 (1812)	EU340081 (549)	Mincks et al. (2009)
*Prionospio* sp. F	–	Clarion Clipperton Fracture Zone	–	MN441542 (405)	Guggolz et al. (2020)
*Prionospio* sp. G	–	Eastern Vema Fracture Zone	MN447188 (844)	MN441564 (397)	Guggolz et al. (2020)
*Prionospio* sp. H	–	Clarion Clipperton Fracture Zone/ eastern Vema Fracture Zone	MN447158 (844)	MN441554 (411)	Guggolz et al. (2020)
*Prionospio* sp. I	–	Puerto Rico Trench	MN447157 (844)	MN441749 (409)	Guggolz et al. (2020)
*Prionospio* sp. K	–	Clarion Clipperton Fracture Zone	–	MN441555 (413)	Guggolz et al. (2020)
*Prionospio* sp. L	–	Western Vema Fracture Zone	MN447168 (844)	MN441745 (408)	Guggolz et al. (2020)
*Prionospio* sp. M	–	Eastern Vema Fracture Zone	MN447160 (844)	MN441561 (411)	Guggolz et al. (2020)
*Prionospio* sp. N	–	Eastern Vema Fracture Zone	MN447180 (844)	MN441604 (342)	Guggolz et al. (2020)
*Prionospio* sp. O	–	Eastern Vema Fracture Zone	MN447159 (844)	MN441748 (342)	Guggolz et al. (2020)
*Prionospio* sp. P	–	Eastern Vema Fracture Zone	MN447173 (844)	MN441753 (339)	Guggolz et al. (2020)
*Prionospio* sp. KJO-2005	–	Monterey Bay, CA, USA	DQ209226 (1703)	–	Osborn et al. (2007)
*Pygospio* Claparède, 1863					
*Pygospio elegans* Claparède, 1863	Normandy, France	Russia	KJ747074 (1719)	KJ747084 (468)	Radashevsky et al. (2016b)
*Pygospio* sp. 1 (as *Pygospio* sp. 2583)	–	Russia	KP940584 (1709)	KP940582 (306)	Radashevsky et al. (2016b)
*Pygospio* sp. 2 (as *Pygospio* sp. VVP-2014)	–	USA	KJ747077 (1756)	KJ747087 (306)	Radashevsky et al. (2016b)
*Rhynchospio* Hartman, 1936					
*Rhynchospio arenicola* Hartman, 1936	CA, USA	USA	KJ546286 (1737)	KJ546318 (341)	Radashevsky et al. (2014)
Rhynchospio aff. asiatica sensu Radashevsky et al. (2014)	–	South Korea	KJ546296 (1731)	KJ546345 (492)	Radashevsky et al. (2014)
*Rhynchospio darwini* Radashevsky, 2015 (as *Rhynchospio* sp. 44)	Australia	Australia	KP986493 (1789)	KP986492 (316)	Radashevsky et al. (2016a)
Rhynchospio cf. foliosa Imajima, 1991 (as *Rhynchospio foliosa*)	Japan	USA	KP986489 (1765)	KP986488 (450)	Radashevsky et al. (2016a)
*Rhynchospio glutaea* (Ehlers, 1897)	Strait of Magellan, Chile	Argentina	KJ546281 (1747)	KJ546332 (341)	Radashevsky et al. (2014)
*Rhynchospio mzansi* Simon, Williams & Henninger, 2018	South Africa	South Africa	MF625258 (1662)	MF625254 (290)	Simon et al. (2019b)
*Rhynchospio nhatrangi* Radashevsky, 2007	Vietnam	Vietnam	KJ546299 (1717)	KJ546343 (499)	Radashevsky et al. (2014)
*Scolelepis* Blainville, 1828					
*Scolelepis acuta* (Treadwell, 1914)	San Diego, USA	Brazil	KU900479 (683)	–	Rebelo & Schettini (unpubl.)
*Scolelepis bonnieri* Mesnil, 1896	English Chanel	Helgoland, Germany	EU084878 (1711)	–	Vortsepneva et al. (2008)
*Scolelepis chilensis* (Hartmann-Schröder, 1962)	Chile	Brazil	KU900475 (689)	–	Rebelo & Schettini (unpubl.)
*Scolelepis daphoinos* Zhou, Ji & Li, 2009	China	China	–	GU362676 (461)	Zhou et al. (2010)
*Scolelepis eltaninae* Blake, 1983	Ross Sea	Antarctica	KF713431 (333)	KF713470 (398)	Gallego et al. (2014)
*Scolelepis goodbodyi* (Jones, 1962)	Jamaica	Brazil	KU900477 (441)	–	Rebelo & Schettini (unpubl.)
*Scolelepis kudenovi* Hartmann-Schröder, 1981	Australia	Lizard Island, Australia	KP636517 (464)	–	Meißner and Götting (2015)
*Scolelepis laonicola* (Tzetlin, 1985) (as *Asetocalamyzas laonicola*)	White Sea, Russia	White Sea, Russia	EF569206 (1323)	–	Vortsepneva et al. (2008)
*Scolelepis squamata* (Müller, 1806)	Denmark	Sylt, Germany	AF448164 (1848)	–	Bleidorn et al. (2003)
*Scolelepis* sp. sco206	–	Eastern Arabian Sea, India	KT900310 (1759)	–	Rengaiyan and Ingole (2018)
*Scolelepis* sp. sco207	–	Eastern Arabian Sea, India	KT900311 (1759)	–	Rengaiyan and Ingole (2018)
*Spiophanes* Grube, 1860					
*Spiophanes berkeleyorum* Pettibone, 1962	Vancouver Island, Canada	California, USA	MN186816 (1724)	–	Radashevsky et al. (2020a)
*Spiophanes bombyx* (Claparède, 1870)	Gulf of Naples, Italy	Adriatic Sea, Italy	–	MG878899 (484)	Radashevsky et al. (2020a)
Spiophanes cf. convexus Delgado-Blas, Díaz-Díaz & Viéitez, 2019	Ria de Vigo, Spain	Brittany, France	MG913229 (1742)	MG878902 (505)	Radashevsky et al. (2020a)
*Spiophanes duplex* (Chamberlin, 1919) (as *Spiophanes berkeleyorum* isolate 20548.2)	California,USA	California,USA	MN186817 (1682)	–	Radashevsky et al. (2020a)
*Spiophanes hakaiensis* Radashevsky & Pankova in Radashevsky et al. 2020	BritishColumbia, Canada	California, USA	MG913241 (1746)	MG878914 (369)	Radashevsky et al. (2020a)
Spiophanes cf. kroyeri Grube, 1860	Greenland Sea, NW Atlantic	BarentsSea,Norway	MG913238 (1738)	MG878907 (340)	Radashevsky et al. (2020a)
Spiophanes aff. kroyeri Grube, 1860 (as *Spiophanes kroeyeri*)	Greenland Sea, NW Atlantic		EU340094 (1769)	EU340080 (544)	Mincks et al. (2009)
*Spiophanes norrisi* Meißner & Blank, 2009	Mexico	USA	GQ202716 (535)	–	Meißner and Blank (2009)
*Spiophanes pisinnus* Meißner & Hutchings, 2003	New South Wales, Australia	Australia	GQ202721 (534)	–	Meißner and Blank (2009)
*Spiophanes soederstromi* Hartman, 1953	off Rio Grande do Sul, Brazil	Paraná, Brazil	MG913232 (1735)	MG878905 (340)	Radashevsky et al. (2020a)
*Spiophanes uschakowi* Zachs, 1933	northern Sea of Japan, Russia	Russia	KM998760 (1747)	MG878915 (342)	Radashevsky et al. (2020a)
*Spiophanes viriosus* Meißner & Hutchings, 2003	Australia	Lizard Island, Australia	KP636519 (451)	–	Meißner and Götting (2015)
*Spiophanes* sp. A	–	East China Sea, South Korea	MG913244 (1732)	MG878920 (417)	Radashevsky et al. (2020a)
*Spiophanes* sp. RG-2014	–	Antarctica	KF713435 (318)	KF713474 (372)	Gallego et al. (2014)
*Streblospio* Webster, 1879					
*Streblospio benedicti* Webster, 1879	New Jersey, USA	Netherlands	KC686673 (411)	–	van Pelt-Heerschap (unpubl.)
*Streblospio* sp.	–	India	KY704336 (578)	KY704328(523)	Vijapure et al. (unpubl.)
*Trochochaeta* Levinsen, 1884					
*Trochochaeta multisetosa* (Örsted, 1844)	Danmark	Askeröfjord, Sweden/North Sea, Norway	MN296517 (1728)	MN193552 (341)	Radashevsky et al. (2020a)
Spioninae Söderström, 1920					
*Boccardia* Carazzi, 1893					
*Boccardia perata* (Chlebovitsch, 1959)	Kurile Islands	Sea of Japan, Russia	–	MH493047 (473)	Radashevsky et al. (2019)
*Boccardia polybranchia* (Haswell, 1885)	New South Wales, Australia	South Africa	KY677891 (1714)	–	Williams et al. (2017)
*Boccardia proboscidea* Hartman, 1940	CA, USA	CA, USA	KJ546254 (1763)	MH493027 (435)	Radashevsky et al. (2014, 2019)
*Boccardia pseudonatrix* Day, 1961	Knysna Estuary, South Africa	South Africa	KY677895 (1719)	–	Williams et al. (2017)
*Boccardiella* Blake & Kudenov, 1978					
*Boccardiella hamata* (Webster, 1879)	USA	Incheon, South Korea	MT482710 (1741)	–	Lee et al. (2020)
*Dipolydora* Verrill, 1881					
*Dipolydora bidentata* (Zachs, 1933)	northern Sea of Japan, Russia	Peter the Great Bay, Russia	JX228065 (900)	JX228103 (475)	Radashevsky and Pankova (2013)
*Dipolydora capensis* (Day, 1955)	South Africa	South Africa	KY677896 (1714)	–	Williams et al. (2017)
		South Africa	KY677897 (1714)	–	Williams et al. (2017)
*Dipolydora cardalia* (E. Berkeley, 1927)	British Columbia, Canada	Peter the Great Bay, Sea of Japan	JX228073 (900)	JX228113 (475)	Radashevsky and Pankova (2013)
*Dipolydora carunculata* (Radashevsky, 1993)	Vostok Bay, Russia	Peter the Great Bay, Sea of Japan	JN048711 (942)	JN048698 (475)	Radashevsky and Pankova (2013)
*Dipolydora quadrilobata* (Jacobi, 1883)	Kiel Canal, Germany	Russia	–	MH493041 (309)	Radashevsky et al. (unpubl.)
Dipolydora cf. socialis (Schmarda, 1861)	Chile	South Africa	KY677899 (1715)	–	Williams et al. (2017)
*Microspio* Mesnil, 1896					
*Microspio granulata* Blake & Kudenov, 1978	Australia	Lizard Island, Australia	KP636515 (457)	KP636514 (362)	Meißner and Götting (2015)
*Polydora* Bosc, 1802					
*Polydora brevipalpa* Zachs, 1933	northern Sea of Japan	China	KP231289 (1725)	–	Ye et al. (2019)
*Polydora cornuta* Bosc, 1802	South Carolina	Netherlands	KC686637 (421)	–	van Pelt-Heerschap (unpubl.)
*Polydora neocaeca* Williams & Radashevsky, 1999 (as *Polydora haswelli*)	Rhode Island	China	KF562242 (1792)	KF562235 (511)	Ye et al. (2019)
*Polydora lingshuiensis* Ye, Tang, Wu, Su, Wang, Yu & Wang, 2015	China	China	KF562240 (1791)	KF562233 (462)	Ye et al. (2015)
Polydora cf. nuchalis Woodwick, 1953	California, USA	South Africa	KY677903 (1715)	–	Williams et al. (2017)
*Polydora triglanda* Radashevsky & Hsieh, 2000	Taiwan	Taiwan	JN048718 (941)	JN048705 (475)	Radashevsky and Pankova (2013)
Polydora cf. websteri Hartman in Loosanoff & Engle, 1943	Milford Harbor, USA	South Africa	KY677904 (1716)	–	Williams et al. (2017)
*Polydorella* Augener, 1914					
*Polydorella dawydoffi* Radashevsky, 1996	South China Sea	Nha Trang Bay, Vietnam	–	MG460900 (308)	Radashevsky et al. (2020b)
*Pseudopolydora* Czerniavsky, 1881					
*Pseudopolydora achaeta* Radashevsky & Hsieh, 2000	Taiwan	Erhjen River, Tainan, Taiwan	–	MG460903 (304)	Radashevsky et al. (2020b)
*Pseudopolydora bassarginensis* (Zachs, 1933)	northern Sea of Japan	Vostok Bay, Sea of Japan, Russia	–	MG460894 (306)	Radashevsky et al. (2020b)
*Pseudopolydora dayii* Simon, 2009	South Africa	South Africa	KY677907 (1716)		Williams et al. (2017)
*Pseudopolydora diopatra* Hsieh, 1992	Taiwan	Hsinchu, Taiwan	–	MG460906 (308)	Radashevsky et al. (2020b)
*Pseudopolydora eriyali* Simon, Sato-Okoshi & Abe, 2017	South Africa	South Africa	AB973933 (1713)	LC107863 (471)	Simon et al. (2019)
*Pseudopolydora kempi japonica* Imajima & Hartman, 1964	Japan	Vostok Bay, Sea of Japan, Russia	–	MG460897 (306)	Radashevsky et al. (2020b)
*Pseudopolydora paucibranchiata* (Okuda, 1937)	Japan	Gulf of Naples, Italy	–	MG460937 (455)	Radashevsky et al. (2020b)
*Pseudopolydora pulchra* (Carazzi, 1893)	Gulf of Naples, Mediterranean	Bay of Morlaix, Brittany, France	–	MG460932 (471)	Radashevsky et al. (2020b)
*Pseudopolydora uphondo* Simon, Sato-Okoshi & Abe, 2017	South Africa	South Africa	LC107848 (1711)	LC107866 (472)	Simon et al. (2019)
*Pseudopolydora vexillosa* Radashevsky & Hsieh, 2000 (as *Pseudopolydora* sp. B)	Taiwan	Mung Is., Nha Trang Bay, Vietnam	–	MG460890 (295)	Radashevsky et al. (2020b)
*Pseudopolydora* sp. A	–	Northern Territory, Australia	–	MG460921 (296)	Radashevsky et al. (2020b)
*Pseudopolydora* sp. B (as *Pseudopolydora* sp. C)	–	Arabian Gulf, Kuwait	–	MG460957 (295)	Radashevsky et al. (2020b)
*Pseudopolydora* sp. C (as *Pseudopolydora* sp. D)	–	Arabian Gulf, Kuwait	–	MG460941 (309)	Radashevsky et al. (2020b)
*Pseudopolydora* sp. D (as *Pseudopolydora* sp. E)	–	Raunefjord, North Sea, Norway	–	MG460960 (305)	Radashevsky et al. (2020b)
*Pseudopolydora* sp. Sodwana 32-4	–	South Africa	LC107849 (1724)	LC107867 (473)	Simon et al. (2019)
*Spio* Fabricius, 1785					
*Spio arndti* Meißner, Bick & Bastrop, 2011 (as *Spio* sp. LK-2011-2)	Baltic Sea	Baltic Sea	FR823434 (1765)	FR823439 (453)	Meißner et al. (2011)
*Spio blakei* Maciolek, 1990	Botany Bay, New South Wales, Australia	Lizard Island, Australia	KP636507 (458)	KP636502 (348)	Meißner and Götting (2015)
*Spio filicornis* (O. F. Müller, 1776)	Iluilârssuk, Greenland	Iluilârssuk, Greenland	FR823431 (1765)	FR823436 (454)	Meißner et al. (2011)
*Spio symphyta* Meißner, Bick & Bastrop, 2011 (as *Spio* sp. LK-2011-1)	North Sea	North Sea	FR823433 (1766)	FR823438 (453)	Meißner et al. (2011)
*Spio* sp. 2573	–	Koni Peninsula, Sea of Okhotsk, Russia	KT200135 (1688)	KT200126 (310)	Radashevsky et al. (2016b)
Outgroup					
Sabellidae Latreille, 1825					
*Laonome* Malmgren, 1866					
*Laonome* sp.	–	Pärnu Bay, Baltic Sea	KP793139 (1813)	KP793138 (450)	Kotta et al. (2015)
*Sabella* Linnaeus, 1767					
*Sabella pavonina* Savigny, 1822	Plymouth	–/Brittany, France	U67144 (1726)	AY340482 (476)	Nadot & Grant (unpubl.), Rousset et al. (2007)

## Results

### The 18S and 16S rRNA gene analyses of larval and adult spionids

Nuclear 18S and mitochondrial 16S rRNA gene sequences of adult spionid polychaetes were successfully obtained from 48 species belonging to 14 genera (Table 1).

In the phylogenetic analysis using only the sequences obtained in the present study (i.e., without DBJ/ENA/GenBank sequences), species from the genera *Paraprionospio*, *Pseudopolydora*, *Scolelepis*, *Spio*, and *Spiophanes* were recovered as monophyletic groups with robust statistical supports (i.e., SH-aLRT ≥ 80%, aBayes ≥ 0.95, and UFBoot ≥ 95%, Fig. 2). Species belonging to the genus *Boccardia* except for *B. pseudonatrix* and those from the genus *Dipolydora* except for *D. armata* and D. cf. commensalis were recovered as monophyletic groups with robust statistical supports. The tribe Polydorini and subfamily Spioninae were also recovered as monophyletic although UFBoot of monophyly of the subfamily Spioninae was with low support (≤ 95%).

In the phylogenetic analysis with the sequences obtained in the present study and from DDBJ/ENA/GenBank databases, species belonging to the genera *Poecilochaetus*, *Laonice*, *Marenzelleria*, *Pseudopolydora*, *Pygospio*, *Rhynchospio*, *Scolelepis*, *Spio* + *Microspio*, and *Spiophanes*, were recovered as monophyletic groups with robust statistical supports (Fig. 3). Species belonging to the genus *Polydora* were recovered as a monophyletic group but with low UFBoot support. Tribe Polydorini + *Pygospio*, that plus *Glandulospio*, and *Spiophanes* + *Trochochaeta* were also recovered as monophyletic groups with robust statistical supports. The genera *Poecilochaetus* and *Trochochaeta*, which were previously considered as belonging to the family Poecilochaetidae and Trochochaetidae, respectively, were recovered as ingroup taxa of the family Spionidae with robust statistical supports (Fig. 3).

**Figure 31.**
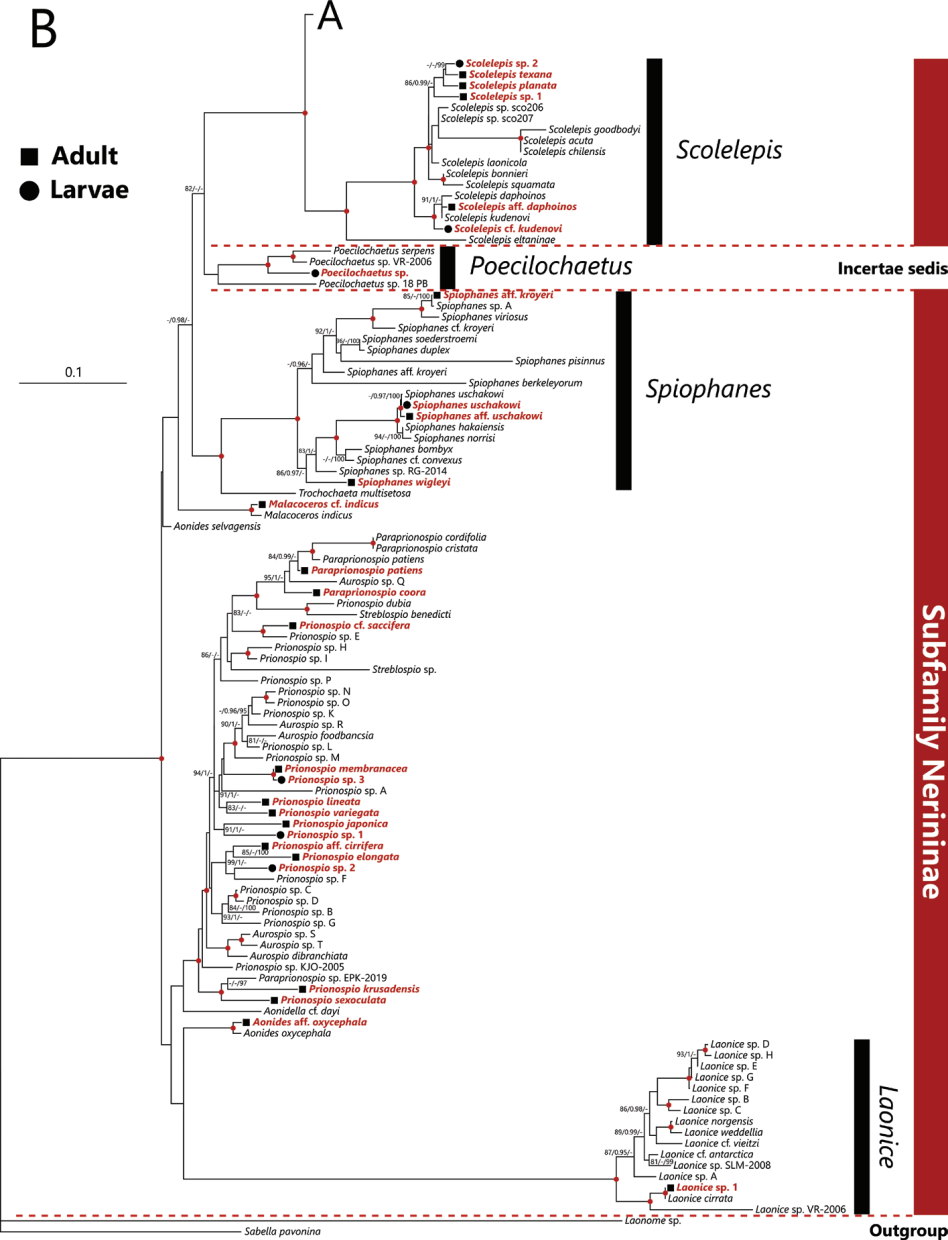
Continued.

In total, 41 species belonging to 14 genera of planktonic spionid larvae were identified (Table 1; Fig. 2), 27 of which were identified by the 100% or nearly 100% match between the sequences obtained from adult and larvae (Fig. 2). The other 14 species of spionid larvae were identified to species or genus level based on their phylogenetic position and/or larval morphology. Tentative larval diagnosis for each genus and larval identification keys to species of each genus based on the morphology of late spionid larvae from northeastern Japan are provided at the beginning of the larval morphological description section of each genus. An identification key to genera is provided below.

### Identification key to genera of the spionid larvae in northeastern Japan

**Table:** 

1	Two pairs of red or dark red eyes present; distinct black pigmentation or melanophore (branching black chromatophores) absent; gastrotrochs from chaetiger II or III onwards in all following chaetigers (except *Rhynchospio*: not in all following chaetigers)	[**subfamily Nerininae] 2**
–	Three pairs of black eyes present (most lateral two pairs of eyes often double-eyes: see Hacker, 1896; Hannerz 1956); distinct black pigmentation present; gastrotrochs from chaetiger III, V, or VII onwards not in all following chaetigers	[**subfamily Spioninae] 8**
–	Two pairs of dark red eyes present; small black pigmentation present laterally between parapodia on every chaetigers; gastrotrochs from chaetiger I onwards in all following chaetigers	**Genus *Poecilochaetus***
2	Lateral parts of peristomium well developed and distinctly demarcated from prostomium	**3**
–	Lateral parts of peristomium less developed and less demarcated from prostomium	**5**
3	Prostomium not pointed anteriorly, more or less stumpy; lateral parts of peristomium not clearly demarcated	**4**
–	Prostomium pointed anteriorly and tip of prostomium terminates in a tapered tip; lateral parts of peristomium clearly demarcated as large peristomial umbrella	**Genus *Scolelepis***
4	Parapodia well differentiated; long larval chaetae only in notopodia	**Genus *Rhynchospio***
–	Parapodia less differentiated; serrated larval chaetae occur in both noto‐ and neuropodia	**Genus *Laonice***
5	Slender, moderately long in overall shape; body not transparent; some pigmentation of various colors on pharynx, proctodaeum, prostomium, peristomium, pygidium, and/or various locations of the body in late larvae	**6**
–	Slender, fairly long in overall shape with numerous chaetigers; body nearly transparent; pigmentation almost completely absent except red or green pigmentation on pharynx and/or pygidium	**7**
6	Body not rich in yolk; larval chaetae on first chaetiger medium length; pharynx not colored in black; prostomium rounded anteriorly	**Genus *Spiophanes***
–	Body rich in yolk; larval chaetae on first chaetiger fairly long especially in early larvae; pharynx colored in black; prostomium rectangular anteriorly (prostomium extended and tapered anteriorly in juvenile)	**Genus *Aonides***
7	Prostomium anteriorly rounded; lateral parts of peristomium relatively demarcated from prostomium; quite large and long larvae with well‐developed branchiae in late larvae	**Genus *Paraprionospio***
–	Prostomium anteriorly rounded; lateral parts of peristomium less demarcated from prostomium; long and slender larvae with no or less developed branchiae	**Genus *Prionospio***
8	Overall body shape long and slender	**9**
–	Overall body shape thick/slender and fusiform	**11**
9	Modified chaetae in chaetiger V present in late larvae; larval chaetae on first chaetiger medium length	**10**
–	Modified chaetae in chaetiger V absent; larval chaetae on first chaetiger fairly long	**Genus *Spio***
10	Pairs of large branching dorsal melanophores present	**Genus *Polydora***
–	Pairs of large branching dorsal melanophores absent	**Genus *Dipolydora***
11	Lateral prostomium expansion moderate (except for *Boccardia* sp. 2); mid‐dorsal melanophores arranged in a single row (except *B. pseudonatrix*); vestibule or pharynx pigmented with black or brown	**Genus *Boccardia***
–	Lateral prostomium expand greatly; arrangement of dorsal melanophores not arranged in a single row; vestibule or pharynx not pigmented with black or brown	**12**
12	A mid‐dorsal branching melanophore on first chaetiger absent; more than two pairs of dorsal black pigmentation spots/bands on each chaetiger	**Genus *Boccardiella***
–	A mid‐dorsal branching melanophore on first chaetiger present (except for Pseudopolydora cf. kempi: mid‐dorsal melanophore on first chaetiger usually absent); one or two pairs of dorsal melanophores on each chaetiger	**Genus *Pseudopolydora***

### Description of larval morphology

#### Family SPIONIDAE Grube, 1850

##### Subfamily NERININAE Söderström, 1920

###### Genus Aonides

Taxon classificationAnimaliaSpionidaSpionidae

Claparède, 1864

3D1135AF-0CBE-52D3-AD30-5DDD51523AFD

####### Larval diagnosis.

The overall shape slender. Prostomium rounded or rectangular anteriorly. The lateral parts of the peristomium more or less demarcated from prostomium. Two pairs of red eyes present. Melanophore absent, some brown or dark pigmentation may be present in pharynx and pygidium. Larval chaetae coarsely or slightly serrated. Larval chaetae in first chaetiger very long, extend beyond pygidium in late trochophore and early nectochaete stages. Nototrochs develop in late larval stages. Gastrotrochs occur in all chaetigers from chaetiger II onwards. Two pairs of pygidial cirri develop in late larval stage. The body of early larvae covered by egg envelope, yellowish opaque appearance with abundant yolk. Two parallel rows of encircling vesicles of egg envelope present in pretrochophore and trochophore stages. Holopelagic lecithotrophic development unique among spionids (Hannerz 1956; Blake and Arnofsky 1999, as *Dispio uncinata* Hartman, 1951: see Radashevsky et al. 2011; Blake 2006, as *D. uncinata*).

###### Aonides aff. oxycephala

Taxon classificationAnimaliaSpionidaSpionidae

(Sars, 1862)

92F20D72-D38F-545F-9E2E-EE5A7BF1EDD1

Fig. 4A–C

####### Larval morphology.

Remnants of egg envelope apparent in early trochophore (Fig. 4A). In ten‐chaetiger larvae, egg envelope becomes incorporated into larval cuticle, two pairs of red eyes arranged in an approximately straight line (Fig. 4B). Larval chaetae on first chaetiger long especially in early larvae (Fig. 4A, B). Late larvae long and slender in shape (Fig. 4C). Prostomium rectangular anteriorly in larval stages, considerably extended and tapered in juvenile stage. Lateral parts of peristomium moderately demarcated from prostomium. Black pigment in pharynx. Pigmentation absent except in the eyes and pharynx. Pygidium acquires two pairs of dorsal cirri in late larvae (Fig. 4C).

####### Remarks.

Adult individuals of this species were collected from muddy bottom sediment at 22 m depth in Onagawa Bay in January 2011 and 2012 using a Smith‐McIntyre grab sampler. Adult morphology agrees with the description of *A. oxycephala* by Imajima (1989). *Aonides oxycephala* originally described from Norway has been reported worldwide and is considered cosmopolitan. However, these reports may comprise a series of similar or sibling species, as pointed out by Radashevsky (2015). The gene sequences obtained in the present study were 100% match in 18S rRNA but 8.6% (29/337 bp) different in 16S rRNA from that of *A. oxycephala* from France (MG913226 and MG878895). Therefore, the species collected in the present study was referred to A. aff. oxycephala. The larvae and adults were confirmed to match (18S: 1753/1753, 16S: 447/448 bp) using molecular data (Fig. 2).

Planktonic larvae were found in Onagawa Bay from October to December. In early larval stages, the larvae of this species are similar to those of *Laonice* sp. (Fig. 4D); but larval chaetae are longer, and the body is yolkier and opaquer in this species. The larval morphology of A. aff. oxycephala is similar to that of *A. oxycephala* described by Hannerz (1956). However, the peristomium of the former species is more developed and demarcated from the prostomium compared to the latter. Black pigmentation of the pharynx in late larval stages was not reported by Hannerz (1956).

###### Genus Laonice

Taxon classificationAnimaliaSpionidaSpionidae

Malmgren, 1867

CA1C08E9-BDC0-5129-95A9-6869F7EB3E5F

####### Larval diagnosis.

Overall shape short, thick, and fusiform. Prostomium stumpy, rectangular, notched anteriorly. Lateral parts of peristomium clearly demarcated from prostomium. The short palps attached to outer end of lateral parts of peristomium. Two pairs of red eyes present. Melanophores and pigmentation absent except eyes. Nototrochs absent. Gastrotrochs occur in all chaetigers from chaetiger III onwards. Well‐developed serrated larval chaetae occur both in noto‐ and neuropodia, notochaetae characteristically introverted toward medial line of dorsal side. Early larvae covered by egg envelope (Hannerz 1956; Plate and Husemann 1994).

###### Laonice

Taxon classificationAnimaliaSpionidaSpionidae

sp. 1

AF1F8A13-111E-5B74-86C3-A97E6299C9A3

Fig. 4D

####### Larval morphology.

Remnants of egg envelope apparent in early trochophore (Fig. 4D). Two pairs of red eyes located in approximately a straight line, lateralmost pair larger in early larvae. Parapodia weakly differentiated; serrated larval chaetae introverted toward medial line of dorsal side. The body opaque yellowish with abundant yolk internally. Pigmentation absent except in the eyes.

####### Remarks.

Adult individuals of this species were collected from bottom sediments at 22 m depth in Onagawa Bay in December 2011 using a Smith‐McIntyre grab sampler. To date, two *Laonice* species, *L. cirrata* (Sars, 1851) and *L. japonica* (Moore, 1907) have been recorded from Japan. Sikorski (2002) indicated that *L. cirrata*, a previously presumed widespread species, is probably limited to Norway and adjacent regions. This was supported by a molecular study that suggested previously unrecognized diversity within this species (Bogantes et al. 2018). *Laonice japonica*, originally described as *Spionides japonicus* from Japan and later considered as synonymous with *L. cirrata* (e.g., Söderström 1920; Berkeley and Berkeley 1936; Okuda 1937; Imajima and Hartman 1964; Foster 1971), was reexamined and considered a valid species by Maciolek (2000) and Sikorski (2011). However, even after that, since *L. cirrata* has been recorded from Japan (e.g., Imajima 2006, 2009, 2011), the validity of these records is ambiguous and might represent different species. In addition to these two species, unidentified *Laonice* sp. was also reported from Japan by Imajima (1990c) but it is unclear whether the species is identical to the species reported here. Although the 18S rRNA gene sequences obtained in the present study match (1731/1731 bp, except for gaps) with *Laonice cirrata* sequences from Russia in DDBJ/EMBL/GenBank (KM998754), because taxonomic knowledge on this genus in Japan is still limited and the 18S rRNA gene is relatively conservative, this species was referred to *Laonice* sp. The larvae and adults were confirmed to match (18S: 1754/1754, 16S: 500/504 bp) using molecular data (Fig. 3).

Larvae of this species were rare in the planktonic community found in Onagawa Bay in September 2011 and October 2012. Although two parallel rows of encircling vesicles of egg envelope, similar to those of *Aonides* pretrochophore and trochophore stages, were reported in oocytes of *Laonice* species (Radashevsky and Lana 2009), this characteristic was not observed in early larval stages with egg envelope in the present study (Fig. 4D). Blake (2006) described the larval development of *Laonice* sp. from California; however, the identification of these larvae is doubtful because they seem to lack serrated larval notochaetae introverted toward the medial line of the dorsal side, which are characteristic of *Laonice* larvae.

###### Laonice

Taxon classificationAnimaliaSpionidaSpionidae

sp. 2

811E2BA0-B796-569C-B73C-F780E898D909

Fig. 4E

####### Larval morphology.

Late larvae thick and stumpy in shape (Fig. 4E). Prostomium stumpy, somewhat notched at tip. Lateral parts of peristomium well demarcated from prostomium. In late larvae, two pairs of red eyes are arranged in a trapezoidal shape, the medial pair bigger and situated anteriorly. Short palps developed in late larvae, attached to outer end of lateral parts of peristomium. Parapodia weakly differentiated; serrated larval chaetae in both noto‐ and neuropodia; notochaetae characteristically introverted toward medial line of dorsal side. Gut dark green in color internally. Pigmentation absent except in the eyes.

####### Remarks.

Adult individuals of this species were not collected in the present study. Only one individual of larva of this species were collected in Habu Port in June 2016. Even though the 18S and 16S rRNA gene sequences were not obtained, the larvae were identified as belonging to *Laonice* because the larval morphology of this species agrees with that of *L. cirrata* described by Hannerz (1956) and L. cf. cirrata described by Plate and Husemann (1994). However, the hooded hooks in neuropodia described by Hannerz (1956) were not observed in the specimens of the present study (nor in those reported by Plate and Husemann [1994]); this may be because the larvae collected here were less developed and the hooks were reported only from chaetiger XIV onwards.

###### Genus Paraprionospio

Taxon classificationAnimaliaSpionidaSpionidae

Caullery, 1914

B103F43B-3C03-5595-9F03-849199C8F6A1

####### Larval diagnosis.

Overall shape long and slender, large in size (> 4 mm) and number of chaetigers (> 35 chaetigers) at metamorphosis. Prostomium rounded. Lateral parts of peristomium moderately demarcated from prostomium. Two pairs of red or dark red eyes present. Pigmentation absent except eyes and some reddish pigmentation on pygidium. Nototrochs absent. Gastrotrochs occur in all chaetigers from chaetiger II onwards. Branchiae well developed and elongated in late larvae. Long larval chaetae may be absent in chaetiger II (Berkeley and Berkeley 1961, as *Prionospio*; Carrasco 1976; Yokoyama 1981, 1996; Blake and Arnofsky 1999).

###### Paraprionospio coora

Taxon classificationAnimaliaSpionidaSpionidae

Wilson, 1990

2F96B76A-E586-5439-AE5E-CDAB73AC1DBA

Fig. 5A, B

####### Larval morphology.

**Figure 5.**
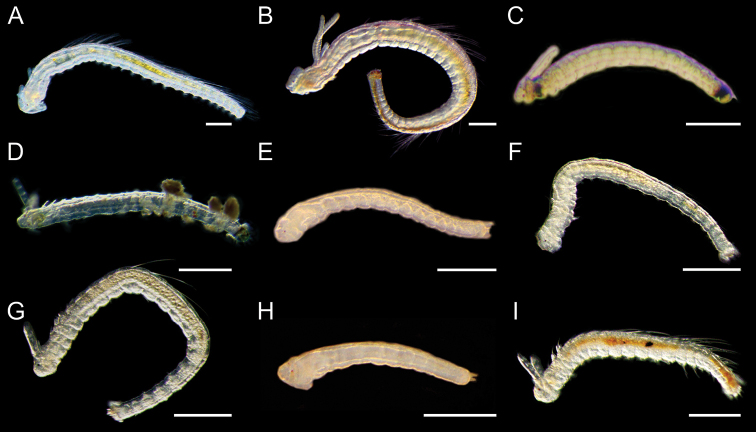
Light micrographs showing the morphologies of living spionid larvae of genera *Paraprionospio* and *Prionospio***A, B***Paraprionospio coora*, lateral view of 25‐chaetiger (**A**) and 33‐chaetiger larvae (**B**) **C, D***Prionospio krusadensis*, lateral view of 17‐chaetiger larvae **E–G***Prionospio membranacea*, lateral view of 15‐chaetiger (**E**), 20‐chaetiger (**F**), and 24‐chaetiger larvae (**G**) **H**, *Prionospio* sp. 1, lateral view of 11‐chaetiger larva **I***Prionospio* sp. 2, lateral view of 19‐chaetiger larva. Scale bars: 300 μm.

Long and thin in shape, quite large and long body with numerous chaetigers. Prostomium anteriorly rounded, lateral lips elevated from the ventrolateral side of prostomium (Fig. 5A). Late larvae acquire caruncle extending posteriorly from posterior part of prostomium (Fig. 5B). Peristomium fuses with the first larval segment at late larval stage (Fig. 5B). First pair of branchiae well developed, branchial pinnation still absent. Two pairs of red eyes arranged in somewhat trapezoidal shape, lateral pair kidney‐shaped, situated anteriorly. Posterior part of pygidium pigmented reddish brown, anal cirri develop in late larvae.

####### Remarks.

Adult individuals of this species were collected from muddy bottom sediments at 22 m depth in Onagawa Bay in December 2011 by using a Smith‐McIntyre grab sampler. Adult morphology agrees with the description of *P. coora* by Yokoyama (2007), and therefore this species was referred to *P. coora*. The larvae and adults were confirmed to match (18S: 1754/1754, 16S: 500/500 bp) using molecular data (Fig. 2).

Only three planktonic larvae of this species were found in Onagawa Bay in November 2011 and Sasuhama in January 2013. The morphological characteristics and size of these larvae are similar to those in previous descriptions of the species from the same genus (Yokoyama 1981, 1996). However, the larvae of *P. coora* lack red pigmentation on the dorsolateral side of the lateral lips, which characterizes the larvae of *Paraprionospio patiens* Yokoyama, 2007 (Yokoyama 1981; as *P. pinnata*: see Yokoyama 2007). Additionally, lamellae of the first pair of branchiae in *P. coora* are less developed in late larvae with more than 30 chaetigers (Fig. 5B) compared with the larvae of *P. patiens* (Yokoyama 1981) and *Paraprionospio cordifolia* Yokoyama, 2007 (Yokoyama 1996, as *Paraprionospio* sp. form B: see Yokoyama 2007). Yokoyama (1981) suggested that the larvae of *Paraprionospio* are the largest in size and number of chaetigers at metamorphosis among the spionid larvae. However, late larvae of *Poecilochaetus* exceeding 5 mm (Magalhães et al. 2015) and with more than 40 chaetigers are often reported (Hannerz 1956; Plate and Husemann 1994).

###### Genus Poecilochaetus

Taxon classificationAnimaliaSpionidaSpionidae

Claparède in Ehlers, 1875

6CB9353B-0B16-5291-AFB7-583DCE98C81E

####### Larval diagnosis.

Overall shape long and slender, large in size (> 5 mm) and number of chaetigers (> 30 chaetigers) at metamorphosis. Body transparent, characterized by total absence of pigmentation except pairs of small pigment spot between parapodia or ventro‐lateral side of each chaetiger. Two pairs of red or dark red eyes present. Gastrotrochs from chaetiger I onwards in all following chaetigers, gastrotrochs in first and second chaetigers represented by solitary lateral patches of cilia and complete gastrotrochs occur from third chaetiger onwards. Nototrochs absent. Larvae prior to ca. 30–40 chaetiger stages remain in metatrochophore stage, characterized by absence of functional parapodia for swimming and presence of well‐developed proto‐, telo‐, and gastrotrochs for swimming. Metatrochophore have broadened trapezoidal prostomium with tactile cilia in anterior part, broad and low caruncle, provisional larval chaetae, pygidium without anal cirri. Larval stage after metatrochophore stage (often called nectosoma) characterized by reduced trochs, the presence of functional parapodia, and rapid serpentine swimming behavior. In nectosoma stage, caruncle, nuchal lobes, a pair of palps, parapodia, cirriform or digitiform dorsal and ventral postchaetal lobes, and two pairs of anal cirri on pygidium develop gradually (Thorson 1946; Hannerz 1956; Berkeley and Berkeley 1961; Reddy and Mohan 1982; Plate and Husemann 1994; Magalhães et al. 2015).

###### Poecilochaetus

Taxon classificationAnimaliaSpionidaSpionidae

sp.

C7ECC31A-EDE0-5ED7-AC1B-7D1713190445

Fig. 4F

####### Larval morphology.

Overall shape long and slender. Two pairs of dark red eyes present. Metatrochophore larvae with 17 chaetigers have broadened trapezoidal prostomium with tactile cilia in anterior part, broad and low caruncle, provisional larval chaetae, and well developed prototrochs (Fig. 4F). Body of metatrochophore larvae transparent, characterized by small pigment spot between parapodia from chaetiger II onwards. Pygidium without anal cirri. Incomplete anterior fragment of nectosoma larvae characterized by extremely long body, rounded prostomium, broad and low caruncle, reduced prototrochs, parapodia with digitiform dorsal postchaetal lobes, and rapid serpentine swimming behavior. The body of nectosoma larvae transparent and small pigment spot laterally on each side of chaetigers. Occipital antenna and pair of palps not observed.

####### Remarks.

Adult individuals of this species were not collected in the present study. Even though the 18S and 16S rRNA gene sequences obtained from larvae in the present study did not match any of the *Poecilochaetus* sequences from DDBJ/EMBL/GenBank, this species was referred to *Poecilochaetus* sp. because specimens formed a monophyletic clade with the other *Poecilochaetus* species with robust statistical support (Fig. 3).

Planktonic larvae of this species were collected in Onagawa Bay in August 2010 and January 2013. The larval morphology of this species is similar to that of *Poecilochaetus serpens* Allen, 1904 described by Hannerz (1956) and Plate and Husemann (1994). However, the former species differs from the latter by not having yellow chromatophores on the “head” and pygidium as described by Plate and Husemann (1994). The pigmentation pattern of *Poecilochaetus* sp. larvae also differs from that of *Poecilochaetus anterospinus*, which has a pair of ventral green melanophores on the lateral side of each segment, beginning from chaetiger IV throughout (Magalhães et al. 2015).

###### Genus Prionospio

Taxon classificationAnimaliaSpionidaSpionidae

Malmgren, 1867

7D568C5B-54FA-57A7-8F92-336DF8773DFC

####### Larval diagnosis.

Overall shape long and slender. Prostomium rounded anteriorly. Lateral parts of peristomium not demarcated from the prostomium. Two pairs of red or dark red eyes present. Pigmentation usually absent except for eyes and on pygidium. Some species (e.g., *P. steenstrupi* and *P. krusadensis*) have red or green pigmentation on pharynx, dorsal side, and/or pygidium. Nototrochs absent or occur in branchial chaetigers in late larvae. Gastrotrochs occur in all chaetigers from chaetiger II–IV onwards (Thorson 1946, as *Disoma*; Hannerz 1956; Plate and Husemann 1994; Radashevsky et al. 2006).

#### Identification key to species of the larvae belonging to the genus *Prionospio* in northeastern Japan

**Table:** 

1	Green pigmentation on pharynx and pygidium present	***Prionospio krusadensis***
–	Green pigmentation on pharynx and pygidium absent	***Prionospio membranacea* or *Prionospio* spp. 1 and 2**

##### Prionospio krusadensis

Taxon classificationAnimaliaSpionidaSpionidae

Fauvel, 1929

2D3D9DC9-D877-5508-B514-2AC40513FF66

Fig. 5C, D

###### Larval morphology.

Long and thin in shape. Prostomium rounded anteriorly, lateral parts of the peristomium not especially well demarcated. Two pairs of red eyes arranged somewhat in trapezoidal shape, lateral pair kidney‐shaped and situated anteriorly. Body extremely transparent (Fig. 5D). Green pigmentation on pharynx and pygidium (Fig. 5C, D). Caruncle develop in late larvae, extends posteriorly from posterior part of prostomium (Fig. 5C). In late larvae, branchial anlages occur from chaetiger II, pygidium acquires anal cirri.

###### Remarks.

Adult individuals of this species were collected from shallow subtidal muddy bottom sediments in Sasuhama in August 2011 by using a hand‐scoop. The species was referred to *P. krusadensis* as the adult morphology agrees with the descriptions of this species by Imajima (1990a). The larvae and adults were confirmed to match (18S: 1749/1749, 16S: 506/507 bp) using molecular data (Fig. 2).

Planktonic larvae of this species were found in Sasuhama and Onagawa Bay in July and August, but they were rare in the plankton samples. Green pigmentation on the pharynx and pygidium is characteristic of the larvae of this species.

##### Prionospio membranacea

Taxon classificationAnimaliaSpionidaSpionidae

Imajima, 1990

EBD5ACCC-440D-5B40-850C-F2A1A775CBA1

Fig. 5E–G

###### Larval morphology.

Long and thin in shape. Prostomium rounded anteriorly, lateral parts of peristomium not especially well demarcated. Two pairs of red or dark red eyes arranged somewhat in trapezoidal shape, lateral pair kidney‐shaped and situated anteriorly. Small caruncle develop in late larvae, extends posteriorly from posterior part of prostomium (Fig. 5G). In late larvae branchial anlages occur from chaetiger II, pygidium acquires anal cirri (Fig. 5G). Palps not yet developed in 15- and 20‐chaetiger larva (Fig. 5E, F) but developed in 24‐chaetiger larva (Fig. 5G). Pigmentation absent except eyes.

###### Remarks.

Adult individuals of this species were collected from muddy bottom sediments at 22 m depth in Onagawa Bay in December 2011 by using a Smith‐McIntyre grab sampler. The species was referred to *P. membranacea* as the adult morphology agrees with the descriptions of this species by Imajima (1990b). The larvae and adults were confirmed to match (18S: 1752/1752 and 1747/1750 except for gaps, 16S: 502/505 bp) using molecular data (Fig. 2). Planktonic larvae of this species were found in Onagawa Bay during August to October.

##### Prionospio

Taxon classificationAnimaliaSpionidaSpionidae

spp. 1 and 2

32472EAF-760E-5157-9E91-C5DDCB61A440

Fig. 5H, I

###### Larval morphology.

Long and thin in shape. Prostomium rounded anteriorly, lateral parts of the peristomium not especially well demarcated. Two pairs of red or dark red eyes arranged somewhat in trapezoidal shape, lateral pair kidney‐shaped and situated anteriorly. Small caruncle develop in late larvae, extends posteriorly from posterior part of the prostomium. In *Prionospio* sp. 2, branchial anlages occur from chaetiger II, pygidium acquires anal cirri (Fig. 5I). Pigmentation absent except eyes in *Prionospio* sp. 1. Gut pigmented in orange in *Prionospio* sp. 2. Palps developed in 19‐chaetiger larva of *Prionospio* sp. 2.

###### Remarks.

Two unidentified species of planktonic larvae of the genus *Prionospio* other than *P. krusadensis* and *P. membranacea* were collected from Onagawa Bay. The adult individuals of these species were not collected in the present study. Even though the 18S and 16S rRNA gene sequences obtained from these larvae in the present study did not match any *Prionospio* sequences obtained in the present study nor with those registered in DDBJ/EMBL/GenBank, these species were referred to *Prionospio* sp. 1 and 2 as the larvae were similar to the other *Prionospio* species in their morphology and gene sequences (Figs 2, 3). The larvae of *Prionospio* sp. 1 and 2 are similar to each other and to that of *P. membranacea*, and it is difficult to distinguish among them based only on their morphology.

##### Genus Rhynchospio

Taxon classificationAnimaliaSpionidaSpionidae

Hartman, 1936

6DB97073-12E2-5051-A8E4-90E5707D79F8

###### Larval diagnosis.

Overall body shape short and thick. Prostomium broad and straight or slightly notched anteriorly. Lateral parts of peristomium clearly demarcated from prostomium. Palps attached to outer end of lateral parts of peristomium. Two pairs of red or black eyes present. Faint yellow pigment may be present in anterior part of prostomium and posterior part of pygidium. In late larvae, pair of prominent antero‐lateral processes on prostomium developed. Melanophore absent, black or yellowish pigmentation occur in some species. Nototrochs weakly developed, occur in all chaetigers except first chaetiger. Gastrotrochs occur regularly in every other chaetiger from chaetiger III onwards (Carrasco 1976; Radashevsky 2007).

##### Rhynchospio aff. asiatica

Taxon classificationAnimaliaSpionidaSpionidae

sensu Radashevsky et al., 2014

C8C4A764-9BB2-5708-BA21-76427956243D

Figs 4G, H , 6

###### Larval morphology.

Overall body shape short and thick in relation to length. Prostomium broad, stumpy, somewhat notched anteriorly. Peristomium well developed, forming wide collar on sides of prostomium. Two pairs of red or dark red eyes arranged in straight line, lateral pair in kidney‐shape. Parapodia strongly differentiated in late larvae. Larval chaetae occur only in notopodia. Pygidium large and round, acquires dorsal cirri in late larvae. Late larvae have two antero‐lateral processes on prostomium (Fig. 4H). Pigmentation usually absent, some individuals have brownish pigmentation on peristomium, dorsum, and/or pygidium, and/or two medial black pigmentation ventrally on approximately chaetiger VI and anterior margin of the pygidium.

###### Remarks.

Adult individuals of this species were collected from intertidal and shallow subtidal sandy or muddy bottom sediments in Gamo Lagoon in January 2011 and Sasuhama in September 2011 by using a hand‐scoop. To date, three *Rhynchospio* species, *R. foliosa* Imajima, 1991, *R. gutaea* (Ehlers, 1987), and *R. tuberculata* Imajima, 1991, have been recorded from Japan (Imajima 1991a). However, extensive morphological and molecular studies revealed the absence of records of *R. glutaea* from the northern Pacific Ocean (Radashevsky 2007; Radashevsky et al. 2014, 2016a). Radashevsky et al. (2014) also referred to *R. arenicola* Hartman, 1936, *R. asiatica* Chlebovisch, 1959, R. aff. asiatica, and *R. glutaea* as members of the *R. glutaea* complex because they resembled each other so closely. Adult morphology and 18S and 16S rRNA gene sequences of *Rhynchospio* specimens obtained in the present study agree (18S: 1716/1716, 16S: 486/492 bp) with those of R. aff. asiatica (Fig. 3) from South Korea (KJ546296) reported by Radashevsky et al. (2014); therefore, this species was referred to R. aff. asiatica sensu Radashevsky et al. (2014). The larvae and adults were confirmed to match (18S: 1783/1783, 16S: 471/477 bp) using molecular data (Fig. 2).

**Figure 6.**
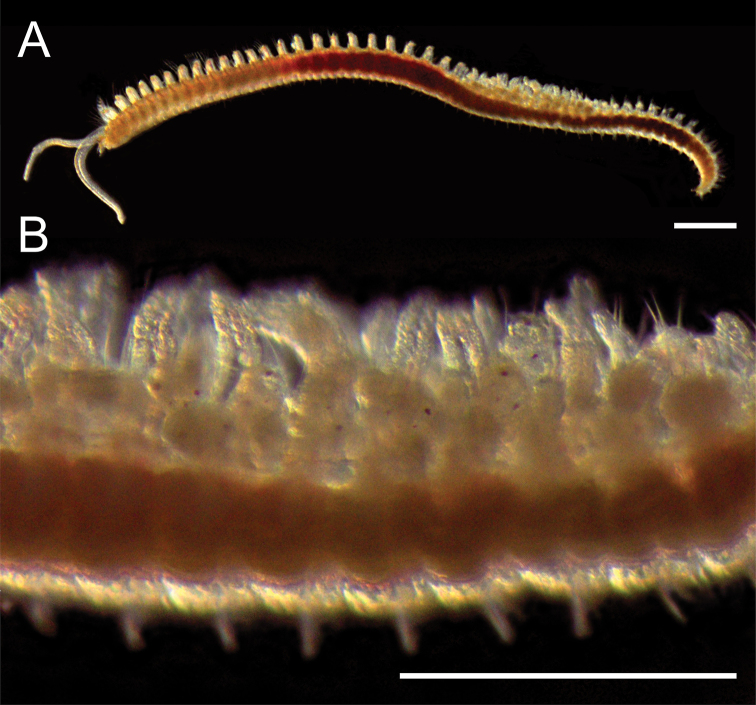
Light micrographs showing dorsal brooding of Rhynchospio aff. asiatica. Hermaphroditic individual broods their larvae between dorsal branchiae on posterior chaetigers. Scale bars: 1 mm.

This species is recorded from Japan for the first time in the present study. The brooding of larvae beneath dorsal branchiae in this species was observed in September 2011 (Fig. 6). The larvae adhere to their parents and are enclosed by branchiae present on the posterior chaetigers (26^th^–39^th^ chaetigers). The larvae are retained on the parents’ dorsum even when the parent individuals leave their tube, unless the parent is disturbed. Larvae seemed to be released at around the 3‐chaetiger stage; the fact that planktonic larvae with more than three chaetigers were commonly collected from plankton supports this observation. Similar dorsal larval brooding was reported in other *Rhynchospio* species (Levin 1982; Radashevsky 2007; Radashevsky et al. 2014) and in *Streblospio benedicti* Webster, 1879 (Levin 1982, 1984).

Planktonic larvae were found in Onagawa Bay, Gamo Lagoon, and Sasuhama in almost every season of the study period, but few were found in winter season (November to March). Larval morphology of this species resembles that of *R. glutaea* and *R. nhatrangi* Radashevsky, 2007 described by Carrasco (1976) and Radashevsky (2007), respectively. The overall larval morphology of *Rhynchospio* species is quite similar to that of the genus *Malacoceros* described in Hannerz (1956, as *Scolelepis*), but it differs in the latter having three pairs of black eyes, the most lateral pairs with double-eyes.

##### Genus Scolelepis

Taxon classificationAnimaliaSpionidaSpionidae

Blainville, 1828

CD49C972-C018-59F4-9C05-F020ED5EB12E

###### Larval diagnosis.

Overall shape thick and fusiform. Prostomium pointed anteriorly, terminates in retractile, muscular tip. Lateral parts of peristomium clearly demarcated from prostomium, forming large peristomial umbrella. Short palps on lateral-most parts of peristomium. Two pairs of red eyes present. Melanophore absent, black, brown, orange, red, or green pigmentation patches often present in body surface, pharynx, gut, and/or proctodaeum. Nototrochs present or absent. Gastrotrochs occur in all chaetigers from chaetiger II or III onwards. Pygidium large, inflated, and surrounded by thick telotroch (Okuda 1946, as *Spio filicornis*; Hartman 1941, as *Nerinides*; Thorson 1946, as *Nerine* in part see Hannerz 1956; Hannerz 1956, as *Nerine* and *Nerinides*; Imajima 1959, as *Nerinides*; Dean and Hatfield 1963, as *Nerinides*; Carrasco 1976, as *Nerine* and *Nerinides*; Plate and Husemann 1994; Scheltema et al. 1997; Blake and Arnofsky 1999; Blake 2006).

#### Identification key to species of the larvae belonging to the genus *Scolelepis* in northeastern Japan

**Table:** 

1	Pharynx and pygidium colored green; pygidium very broad and horseshoe‐shaped	**Scolelepis cf. kudenovi**
–	Pharynx pigmented orange and gut pigmented brown; Pygidium broad and spherical shaped	**2**
2	Prostomium sharply tapered anteriorly; gut diverticula not strongly segmented	***Scolelepis* sp. 1**
–	Prostomium bluntly tapered anteriorly; gut diverticula strongly segmented	***Scolelepis* sp. 2**

##### Scolelepis cf. kudenovi

Taxon classificationAnimaliaSpionidaSpionidae

Hartmann‐Schröder, 1981

78E74909-3657-53F7-9129-2CCA6F21A228

Fig. 4I

###### Larval morphology.

Thick and fusiform in shape. Prostomium pointed anteriorly, terminates in retractile, muscular tip. Lateral parts of peristomium clearly demarcated from prostomium, forming peristomial umbrella. Peristomial umbrella carrying well‐developed prototroch. Two pairs of red eyes arranged in somewhat trapezoidal shape, medial pair situated anteriorly. Greenish pigment in pharynx and proctodaeum. Pygidium very broad and horseshoe‐shaped.

###### Remarks.

Only three individuals of early larvae of this species were collected from Sasuhama in January 2012. The 18S rRNA gene sequences obtained in the present study for these specimens match (464/464 bp) that of *S. kudenovi* from Lizard Island, Australia (KP636517: Meißner and Götting 2015). Since the species identification is unreliable because of the short reference sequence, this species was referred to S. cf. kudenovi.

The sequence of an adult individual, which collected from the surf zone of the sandy beach in Rishiri Island and previously identifies as *Scolelepis kudenovi* (Abe et al. 2019c) as the morphology agrees with the descriptions of *S. kudenovi* by Imajima (1992) and Meißner and Götting (2015), 100% matched with that of the larvae of Scolelepis cf. kudenovi in 18S rRNA gene (1819/1819 bp) but largely differed in 16S rRNA gene (462/505 bp). Because the 16S rRNA gene of the adult individual was rather closer to *S. daphoinos* (430/455 bp) from China (GU362676, Zhou et al. 2010), it is referred to S. aff. daphoinos in the present study (Table 1, Figs 2, 3).

##### Scolelepis

Taxon classificationAnimaliaSpionidaSpionidae

sp. 1

64D55BCF-34D9-54D7-AC84-CA00FEE2161A

Fig. 4J, K

###### Larval morphology.

Thick and fusiform in overall shape. Prostomium pointed anteriorly as a small process, tip of prostomium terminates in retractile, muscular tip. Lateral parts of peristomium clearly demarcated from prostomium, forming large peristomial umbrella. Peristomial umbrella carrying well‐developed prototroch. Short palps developed in late larvae, attached on lateral-most parts of peristomium. Two pairs of red eyes arranged in an approximately straight line. Pharynx pigmented orange and the gut pigmented brown. Pygidium broad and spherical.

###### Remarks.

Adult individuals of this species were collected from muddy sediments at 22 m depth in Onagawa Bay in December 2011 by using a Smith‐McIntyre grab sampler. These adults were morphologically identified as *Scolelepis*, but they were not identified to species level as these specimens were all incomplete and in poor condition. As the 18S and 16S rRNA gene sequences obtained in the present study did not match any available *Scolelepis* sequences (Figs 2, 3), this species was referred to *Scolelepis* sp. 1. Planktonic larvae of this species were collected in Onagawa Bay in October during the study period. The larvae and adults were confirmed to match using molecular data (Fig. 3).

##### Scolelepis

Taxon classificationAnimaliaSpionidaSpionidae

sp. 2

9F2CC83E-5E96-5B94-BD57-896CEA99982F

Fig. 4L

###### Larval morphology.

Thick and fusiform in overall shape. Prostomium bluntly pointed anteriorly, terminates in retractile, muscular tip. Lateral parts of peristomium clearly demarcated from prostomium, forming large peristomial umbrella. Peristomial umbrella carrying well‐developed prototroch. Short palps developed in late larvae, attached on lateral-most parts of peristomium. Two pairs of red eyes arranged in an approximately straight line. Pharynx widely pigmented orange and the gut pigmented brown. Pygidium broad and spherical shaped.

###### Remarks.

No adult individuals of this species were collected in the present study. Even though the 18S and 16S rRNA gene sequences obtained from larvae in the present study did not match any available *Scolelepis* sequences, this species was referred to *Scolelepis* sp. 2 as the larvae constitute a monophyletic clade with the other *Scolelepis* species with robust statistical support (Figs 2, 3).

Planktonic larvae of this species were found in Onagawa Bay in September. The larvae of this species are quite similar to those of *Scolelepis* sp. 1; however, the prostomium is more broadly pointed anteriorly and the gut diverticula are more strongly segmented in the former species.

##### Genus Spiophanes

Taxon classificationAnimaliaSpionidaSpionidae

Grube, 1860

314F2221-37AF-54EF-8BF1-D86815EBE8C3

###### Larval diagnosis.

Overall shape slender. Prostomium small or broad, rounded or slightly notched anteriorly. Lateral parts of the peristomium slightly or moderately demarcated from the prostomium, palps on lateral-most parts of peristomium. Two pairs of red eyes present. In late larvae, a pair of prominent or small antero‐lateral processes on prostomium are often developed. Melanophore absent, some pigmentation patches of various colors are present on pharynx, proctodaeum, prostomium, peristomium, pygidium, and/or various locations of the body in late larvae. Nototrochs occur in all chaetigers from chaetigers II–IV onwards. Gastrotrochs occur in all chaetigers from chaetiger II onwards (Thorson 1946; Hannerz 1956; Carrasco 1976; Plate and Husemann 1994; Blake 2006).

#### Identification key to species of the larvae belonging to the genus *Spiophanes* in northeastern Japan

**Table:** 

1	Prostomium small; pharynx and proctodaeum colored in brown; yellow or yellow‐brown pigments on prostomium, peristomium, and pygidium; a pair of small lateral processes on the prostomium developed in late larvae; small red pigment spots present on lateral part of posterior chaetigers	***Spiophanes uschakowi***
–	Prostomium broad; pharynx and proctodaeum colored in black; a pair of prominent lateral processes on the prostomium developed in late larvae; small red pigment spots absent	**Spiophanes aff. uschakowi**

##### Spiophanes uschakowi

Taxon classificationAnimaliaSpionidaSpionidae

Zachs, 1933

209C6A6E-A7E9-50F7-9C7B-D4C9FEACC11A

Fig. 4M, N

###### Larval morphology.

Overall shape slender. Prostomium small and rounded anteriorly. Two pairs of red or dark red eyes present, lateral pair situated anteriorly. Late larvae bear very small antero‐lateral processes on prostomium. Lateral parts of peristomium slightly demarcated from prostomium, palps attached on lateral-most parts of peristomium. Nototrochs occur from chaetiger IV onwards (Fig. 4N). Yellow pigments on the prostomium and pygidium, intense yellow-brown pigment on peristomium, inside of pharynx, and pygidium. Small red pigment spots present on lateral part of body (Fig. 4N). Black pigment in pharynx and proctodaeum absent.

###### Remarks.

No adult individuals of this species were collected in the present study. However, gene sequences obtained from larvae of this species were almost identical (18S: 1732/1732, 16S: 341/342 bp) to that of *S. uschakowi* (KM998760 and MG878915) from Russia (Radashevsky et al. 2020a); therefore, this species was referred to *S. uschakowi*. Imajima (1991b) recorded four *Spiophanes* species from Japan: *S. kroyeri* Grube, 1860 (as *S. kroeyeri*); *S. japonicum* Imajima, 1991; *S. bombyx* (Claparède, 1870); and *S. urceolata* Imajima, 1991. Then, Meißner and Hutchings (2003) synonymized *S. urceolata* with *S. wigleyi* Pettibone, 1962. Additionally, the specimens from Japan formerly identified as *S. bombyx* were morphologically reexamined and identified as S. cf. uschakowi by Meißner and Blank (2009). In the present study, the presence of *S. uschakowi* in Japan was further supported by molecular analysis.

Only a few larvae of this species were collected in Onagawa Bay in November 2011. The overall larval morphology of this species somewhat resembles that of *S. kroyeri* described by Hannerz (1956) in the following aspects: prostomium is relatively small and anteriorly rounded, the peristomium is not quite sharply demarcated from the prostomium, nototrochs occur from chaetiger IV onwards, and the brown pigmentation is present on the pygidium and inside the pharynx. However, *S. kroyeri* lacks small red pigment spots on the lateral part of the body and lateral processes on the prostomium even in 22‐chaetiger larvae.

##### Spiophanes aff. uschakowi

Taxon classificationAnimaliaSpionidaSpionidae

Zachs, 1933

47575CD0-108E-5172-96BE-A0A2071A912C

Fig. 4O, P

###### Larval morphology.

Overall shape slender. Prostomium broad and slightly notched anteriorly. In late larvae, a pair of prominent antero‐lateral processes on prostomium developed. Two pairs of red or dark red eyes present, lateral ones situated somewhat anteriorly. Lateral parts of peristomium moderately demarcated from prostomium, palps on lateral-most parts of peristomium. Nototrochs occur from chaetiger II onwards. Pharynx and proctodaeum black in color internally. Pygidium acquires dorsal cirri in late larvae. Some brownish, yellowish, or greenish pigmentation occurred on various locations of body in late larvae.

###### Remarks.

Adult individuals of this species were collected from muddy bottom sediments at 22 m depth in Onagawa Bay in April and May 2012 by using a Smith‐McIntyre grab sampler and from bottom sediments of the shallow subtidal zone in Sasuhama in February 2012. Adult morphology agrees with the description of S. cf. uschakowi by Meißner and Blank (2009) as well as with that of *S. bombyx* by Imajima (1991b). However, the 18S and 16S rRNA gene sequences of this species did not match those of *S. uschakowi* obtained from DDBJ/EMBL/GenBank (KM998760): there was a 0.29% (5/1750 bp) and 0.88% (3/342) difference, respectively between these two species. Therefore, this species was referred to S. aff. uschakowi. The larvae and adults were confirmed to 100% match using molecular data (Fig. 2).

Planktonic larvae of this species were collected in Onagawa Bay in November 2011 and in Sasuhama in February 2012. The larval morphology of S. aff. uschakowi was different from that of *S. uschakowi* in the following aspects: prostomium of the former is broad and slightly notched anteriorly, whereas that of the latter is relatively small and anteriorly rounded; the peristomium of the former is well demarcated from the prostomium, but that of the latter is relatively less demarcated; nototrochs of the former occur from chaetiger II onwards, whereas those of the latter occur from chaetiger IV onwards; pigmentation inside the pharynx is black in the former but brown in the latter; and black pigmentation in the proctodaeum is present in the former but absent in the latter. Black pigmentation in the pharynx and proctodaeum were also reported in the larvae of *S. bombyx* (Hannerz 1956), S. cf. bombyx (Blake 2006), and *S. duplex* (Chamberlin, 1919) (Blake 2006). However, the illustrations of S. cf. bombyx provided by Blake (2006: fig. 13.10C, D) are seemingly more similar to larvae of *Rhynchospio* than to those of *Spiophanes*.

#### Subfamily SPIONINAE Söderström, 1920

##### Genus Boccardia

Taxon classificationAnimaliaSpionidaSpionidae

Carazzi, 1893

0B4E7FFB-4030-5D72-8A97-E0617E5BC7EA

###### Larval diagnosis.

Overall shape thick or slender and fusiform. Prostomium small or broad and rounded anteriorly. Three pairs of black eyes present, most lateral often double-eyes. Dorsal pigment pattern consists of single row of branching melanophores in most species, some species lack distinct dorsal melanophore. Lateral pigments present or absent. Ventral pigments absent. Nototrochs occur in all chaetigers except first two chaetigers. Gastrotrochs occur in irregular pattern. Modified chaetae develop in chaetiger V in late larvae (Söderström 1920, as *Polydora natrix*; Hartman 1941; Carrasco 1976; Woodwick 1977; Blake and Kudenov 1981; Duchêne 1984, 1989; Guérin 1991; Gibson 1997; Blake and Arnofsky 1999; Gibson and Smith 2004; Blake 2006; Kamel et al. 2010; Oyarzun and Brante 2015; Blake 2017).

#### Identification key to species of the larvae belonging to the genus *Boccardia* in northeastern Japan

**Table:** 

1	Distinct dorsal melanophore absent; faint yellow coloration present on all over body	***Boccardia pseudonatrix***
–	Mid‐dorsal melanophores arranged in a single row	**2**
2	Dorso‐lateral spots of black pigment absent; overall body shape thick and fusiform; pharynx pigmented with black	***Boccardia* sp. 2**
–	Dorso‐lateral spots of black pigment present; overall body shape slender and fusiform; black pigment at pharynx present or absent	**3**
3	A prominent row of mid‐dorsal melanophores from chaetiger III; dorso‐lateral spots of black pigment present on chaetigers VII and VIII; black pigment in pharynx absent	***Boccardia proboscidea***
–	A prominent row of mid‐dorsal melanophores from chaetiger IV; dorso‐lateral spots of black pigment present from chaetiger V onwards; black pigment in pharynx present	***Boccardia* sp. 1**

##### Boccardia proboscidea

Taxon classificationAnimaliaSpionidaSpionidae

Hartman, 1940

D7D65997-7274-5363-9140-B47B5BE54569

Fig. 7A, B

###### Larval morphology.

**Figure 7.**
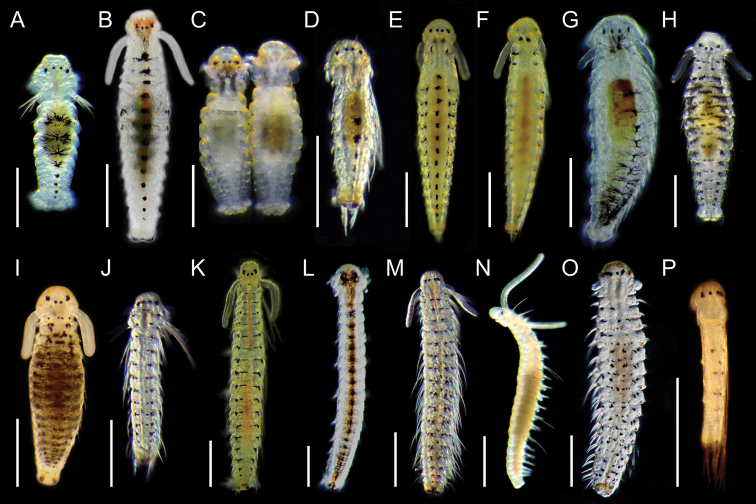
Light micrographs showing the morphologies of living spionid larvae of genera *Boccardia*, *Boccardiella*, and *Dipolydora***A, B***Boccardia proboscidea*, dorsal view of accidentally hatched 9‐chaetiger (**A**) and 15‐chaetiger larvae (**B**) **C***Boccardia pseudonatrix*, dorsal (left) and ventral (right) view of accidentally hatched 10‐chaetiger larvae **D–F***Boccardia* sp. 1, dorsal view of 9‐chaetiger (**D**) and dorsal (**E**) and ventral view (**F**) of 17‐chaetiger larvae **G***Boccardia* sp. 2, dorsal view of 15‐chaetiger larva **H, I***Boccardiella hamata*, dorsal view of 16‐chaetiger (**H**) and 18‐chaetiger larvae (**I**) **J, K***Dipolydora bidentata*, dorsal view of 13‐chaetiger (**J**) and 18‐chaetiger larvae (**K**) **L**Dipolydora cf. commensalis, dorsal view of 21‐chaetiger larva **M, N***Dipolydora giardi*, dorsal view of 19‐chaetiger (**M**) and lateral view of 21‐chaetiger larvae (**N**) **O**Dipolydora cf. socialis, dorsal view of 18‐chaetiger larva **P***Dipolydora* sp., dorsal view of 7‐chaetiger larva. Scale bars: 300 μm.

Slender and fusiform in overall shape, widest in middle of body. Prostomium rounded and slightly notched anteriorly. Three pairs of eyes present, most median pair rounded, lateral pairs double‐eyes. Body entirely faint green in color. A prominent row of dorsal melanophores occurs medially from chaetiger III, lateral black pigment spots present on chaetigers VII and VIII in late larvae (Fig. 7B). Pygidium has dorsal gap, pigmented with weak dark color. Internally, vestibule light brown, gut either yellow or brown. Gastrotrochs on chaetigers V and VII.

###### Remarks.

Adults of this species were non‐boring and collected from mud deposits in crevices of shells of living *Crassostrea gigas* (Thunberg, 1793) (recently assigned to *Magallana*: see Backeljau 2018) oysters in Sasuhama in May 2011 and February 2016. Adult morphology agrees with the description of *B. proboscidea* by Sato‐Okoshi (2000). The 18S and 16S rRNA gene sequences obtained in the present study match (18S: 1748/1748, 16S: 435/435 bp) that of *B. proboscidea* from USA (KJ546254) reported by Radashevsky et al. (2014) (Fig. 3). Therefore, this species was referred to *B. proboscidea*. The larvae and adults were confirmed to match (18S: 1768/1768, 16S: 472/472 bp) using molecular data (Fig. 2).

Planktonic larvae of this species were rare, and only one 15‐chaetiger larva (Fig. 7B) was collected in Sasuhama in May 2011. Another 9‐chaetiger larvae, which accidentally hatched from its egg capsule in an adult tube during the process of extraction of the adult specimens (Fig. 7A), was also collected on the same date. *Boccardia proboscidea* has been reported to have poecilogonous development (Gibson 1997; Oyarzun et al. 2011). However, Sato-Okoshi (2000) reported that Japanese populations only show lecithotrophic development, with no (or a very short) planktonic stage after hatching. The larval morphology of this species agrees with the description of that of *B. proboscidea* documented in Hartman (1941), Woodwick (1977), Blake and Kudenov (1981), Gibson (1997), Gibson and Smith (2004), Kamel et al. (2010), and Oyarzun and Brante (2015). The dorsal pigment pattern of these larvae resembles that of the larvae of *B. tricuspa* (Hartman, 1939) described by Carrasco (1976, as *B. proboscidea*; *fide*Blake and Kudenov 1978), *B. natrix* (Söderström, 1920) described by Söderström (1920, as *Polydora natrix*), and *B. columbiana* Berkeley, 1927 described by Blake and Arnofsky (1999) and Blake (2006) in having a single row of mid‐dorsal melanophores. However, the dorsal pigment pattern of the larvae of *B. tricuspa* differs from that of *B. proboscidea* in having branching dorsal melanophores on chaetiger I and in lacking small black lateral pigment spots on chaetigers VII and VIII. The larvae of *B. natrix* also lack small black lateral pigment spots on chaetigers VII and VIII. *Boccardia columbiana* has extensively branching mid‐dorsal melanophores from chaetiger II onward, whereas mid‐dorsal melanophores are less branching and start from chaetiger III in *B. proboscidea*.

##### Boccardia pseudonatrix

Taxon classificationAnimaliaSpionidaSpionidae

Day, 1961

71A0C086-2E27-5730-9CAF-1E77CC41396A

Fig. 7C

###### Larval morphology.

Slender and fusiform in overall body shape. Prostomium rounded with a slight anterior notch. Three pairs of eyes present, most median pair rounded and lateral pairs double‐eyes. Body entirely faint yellow. Dorsal melanophore absent, slight black pigmentation present (Fig. 7C, left). Pygidium with dorsal gap, pigmented with yellow. Internally, the vestibule and pharynx brown or black, gut green in color. Gastrotrochs on chaetigers V and VII.

###### Remarks.

Adults of this species were non‐boring and collected from mud deposits in crevices of shells of living *C. gigas* oysters in Tomiura. Adult morphology (see Abe et al. 2019b) agrees with the descriptions of *B. pseudonatrix* from South Africa (Day 1967; Simon et al. 2010) and Australia (Sato‐Okoshi et al. 2008, as *B. knoxi*; see Walker 2013). The 18S rRNA gene sequences obtained in the present study completely match (1714/1714 bp) that of *B. pseudonatrix* from South Africa (KY677895) reported by Williams et al. (2017) (Fig. 3). Therefore, this species was referred to *B. pseudonatrix*.

*Boccardia pseudonatrix* has been reported to have adelphophagic larvae with a short or absent planktonic phase (Sato‐Okoshi et al. 2008; Simon 2015). The larvae herein reported accidentally hatched from egg capsules in an adult tube during the process of extraction of the adult specimens.

##### Boccardia

Taxon classificationAnimaliaSpionidaSpionidae

sp. 1

3679DC66-1179-56B8-B596-7E98B1B54AF6

Fig. 7D–F

###### Larval morphology.

Slender and slightly fusiform in overall shape, widest in anterior part of body. Prostomium rounded anteriorly. Three pairs of black eyes present, most median pair rounded and lateral pairs double‐eyes. Body entirely yellowish in color. A prominent row of dorsal melanophores occurs medially from chaetiger IV, lateral black pigment spots present from chaetiger V onwards (Fig. 7E). Pygidium with dorsal gap, pigmented with black color. Vestibule black, gut orange in color internally. Larval chaetae on first chaetiger long especially in early larvae (Fig. 7D). Gastrotrochs on chaetigers III, V, VII, X, and XIII.

###### Remarks.

No adult individuals of this species were collected in the present study. The 18S and 16S rRNA gene sequences obtained from the larvae did not match any available *Boccardia* sequences, but this species is very similar to the other *Boccardia* species in larval morphology and gene sequences (Figs 2, 3). Therefore, this species was referred to *Boccardia* sp. 1.

Planktonic larvae of this species were collected from Onagawa Bay in April, May, November, and December 2011, and January, February, March, and May 2012. The overall body shape of these larvae is slender and slightly fusiform, similar to those of *B. proboscidea*. However, other larval morphological characteristics differ between these two species: overall body color is faint yellow in the former species and faint green in the latter; the larval chaetae are longer in the former species than in the latter, especially in early larvae (Fig. 7D); lateral black spots are present on chaetiger V onwards in the former species but only on chaetigers VII and VIII in the latter species.

##### Boccardia

Taxon classificationAnimaliaSpionidaSpionidae

sp. 2

91822F34-1389-5885-9F5A-054424F87587

Fig. 7G

###### Larval morphology.

Thick and fusiform in overall shape, widest at middle part of body. Prostomium extensively broad and anteriorly rounded. Three pairs of black eyes present, most median pair rounded and lateral pairs double‐eyes. Body entirely faint green in color in late larvae. A prominent row of dorsal ramified melanophores occurs medially from chaetiger IV onwards, lateral black pigment spots absent. Pygidium with dorsal gap, pigmented with weak dark color. Internally, vestibule black, gut orange in color. Gastrotrochs on chaetigers III, V, VII, X, and XIII.

###### Remarks.

No adult individuals of this species were collected in the present study. The 18S and 16S rRNA gene sequences herein obtained from the larvae did not match any of the available *Boccardia* sequences, but this species is similar to the other *Boccardia* species in larval morphology and gene sequences (Figs 2, 3); therefore, this species was referred to *Boccardia* sp. 2.

Planktonic larvae of this species were collected from Onagawa Bay in December 2010 and November and December 2011, from Sasuhama in January 2013, and from Sendai Port in December 2010. The larval morphology of this species differs from that of other *Boccardia* larvae in having a thick and fusiform body shape.

##### Genus Boccardiella

Taxon classificationAnimaliaSpionidaSpionidae

Blake & Kudenov, 1978

480568B0-4DFC-5197-83C3-CC6E28882016

###### Larval diagnosis.

Overall shape thick and fusiform. Prostomium extensively broad and rounded anteriorly. Three pairs of black eyes present, most lateral pairs usually double‐eyes. More than two pairs of dorsal melanophores from chaetiger III onwards. Lateral and ventral pigments present. Nototrochs occur in all chaetigers except first two. Gastrotrochs occur in irregular pattern. Modified chaetae develop in chaetiger V in late larvae (Rullier 1960, as *Polydora redeki*; Dean and Blake 1966, as *Boccardia*).

##### Boccardiella hamata

Taxon classificationAnimaliaSpionidaSpionidae

(Webster, 1879)

CF836F52-0098-5EA1-ACF8-638134F28A46

Fig. 7H, I

###### Larval morphology.

Thick and fusiform in overall shape, widest at middle part of body. Prostomium broad and anteriorly rounded, usually dusky brown anteriorly. Three pairs of black eyes present, most median pair rounded, lateral pairs usually double‐eyes, occasionally divided into respective eyes. Black pigmentation usually presents ventrally on each lateral lip, occasionally absent. Dorsal pigmentation basically consists of a pair of medial bands, lateral branching melanophores, and small pigment patch at the base of notopodia in each chaetiger from chaetiger III onwards (Fig. 7H). These melanophores undergo expansion and contraction, sometimes coalescing to cover almost the whole of the dorsal surface as ramified pigmentation (Fig. 7I). Four transverse lines of black pigmentation sometimes fused as a single transverse band in chaetiger I. One or two pairs of lateral black pigmentation on chaetiger II. Two rows of band‐shaped ventral pigmentation usually located on posterior edges of some chaetigers posterior to second chaetiger. A pair of black pigment patches on pygidium. Gastrotrochs on chaetigers III, V, VII, X, and XIII.

###### Remarks.

Adults of this species were non‐boring and collected from mud deposits in crevices of shells of living *C. gigas* oysters in Sasuhama in May 2011 and February 2016. Adult morphology agrees with the description of *B. hamata* by Sato‐Okoshi (2000). Therefore, this species was referred to *B. hamata*. The larvae and adults were confirmed to match (18S: 1772/1772, 16S: 480/481 bp) using molecular data (Fig. 2).

Planktonic larvae of this species were frequently collected from Onagawa Bay, Gobu‐ura, and Sasuhama in July and August. The larval morphology of this species agrees with that of *B. hamata* described by Dean and Blake (1966, as *Boccardia*).

##### Genus Dipolydora

Taxon classificationAnimaliaSpionidaSpionidae

Verrill, 1879

39451B26-B238-5A08-B833-DEB4A7328E25

###### Larval diagnosis.

Overall shape slender or slightly fusiform. Prostomium small rounded anteriorly. Three pairs of black eyes present, most lateral pairs often double‐eyes. Ramified melanophore between central and lateral pairs of eyes usually absent, but present in some species (e.g., D. cf. commensalis). Dorsal pigment pattern consists of two rows of band or spot shaped melanophores or a transverse row of small melanophores at each chaetiger in most species, while some species have single row of branching mid‐dorsal melanophores (e.g., D. cf. commensalis) or completely lack melanophores (e.g., *D. armata*). Lateral and ventral pigments are present or absent. Nototrochs occur in all chaetigers except the first two chaetigers. Gastrotrochs occur in irregular pattern. Modified chaetae develop in chaetiger V in late larvae (Andrews 1891, as *Polydora*; Hannerz 1956, as *Polydora*; Hatfield 1965, as *Polydora*; Blake 1969, as *Polydora*; Carrasco 1976, as *Polydora*; Day and Blake 1979, as *Polydora*; Radashevsky 1989, as *Polydora*; Plate and Husemann 1994, as *Polydora*; Lewis 1998; Blake 2006; Blake 2017).

#### Identification key to species of the larvae belonging to the genus *Dipolydora* in northeastern Japan

**Table:** 

1	Mid‐dorsal single row of distinct melanophores present	**Dipolydora cf. commensalis**
–	Arrangement of dorsal melanophore otherwise	**2**
2	Black pigmentation on lateral peristomium present; a pair of band‐shaped ventral black pigment present; notopodial lobes tipped with orange pigment in late larvae	**Dipolydora cf. socialis**
–	Black pigmentation on lateral peristomium absent; ventral black pigment absent; notopodial lobes not tipped with orange pigment	**3**
3	Some patchy black pigment between head and first chaetiger present	***Dipolydora bidentata***
–	Black pigment between head and first chaetiger absent	**4**
4	Two pairs of dorsal black pigment spots present; yellow‐brown pigment on anterior margin of prostomium absent	***Dipolydora giardi***
–	A pair of dorsal black pigment spots present; weak yellow‐brown pigment on anterior margin of prostomium present	***Dipolydora* sp.**

##### Dipolydora bidentata

Taxon classificationAnimaliaSpionidaSpionidae

(Zachs, 1933)

C2CBE223-E88C-5248-BD7B-BD487A8A8430

Fig. 7J, K

###### Larval morphology.

Overall shape elongated. Prostomium and pygidium small. Three pairs of black eyes present, most lateral pairs double‐eyes. Black pigmentation patches on lateral peristomium absent. Some patchy black pigment occurs between head and first chaetiger. Two dorsal black bands begin on chaetiger II and continue to posterior end. Dorso‐lateral pigment extend posteriorly along lateral side found on most chaetigers. Some black or brown pigment may occur on pygidium. Ventral pigment absent. Gastrotrochs on chaetigers V, VII, X, XIII, and XV.

###### Remarks.

Adults of this species are shell-borers and were collected from shells of wild *C. gigas* oysters in Sasuhama in July 2012. Adult morphology agrees with the description of *D. bidentata* by Sato‐Okoshi (1999). The 18S and 16S rRNA gene sequences obtained in the present study match (18S: 900/900, 16S: 473/475 bp) that of *D. bidentata* from Russia (JX228065) reported by Radashevsky and Pankova (2013) (Fig. 3). Planktonic larvae of this species were collected from Onagawa Bay in November 2011 and from Sasuhama in February 2012. The larvae and adults were confirmed to 100% match using molecular data (Fig. 2).

##### Dipolydora cf. commensalis

Taxon classificationAnimaliaSpionidaSpionidae

(Andrews, 1891)

5BEAEF98-C1CB-58FE-AFC0-035B88DB4FC8

Fig. 7L

###### Larval morphology.

Overall shape elongated and slender. Prostomium small but wider than body and rounded anteriorly. Three pairs of eyes present, most lateral pairs double‐eyes of kidney‐shaped appearance. Ramified melanophores present around eyes. Black pigmentation on lateral peristomium absent. Median row of ramified melanophores from chaetiger I onwards. Lateral and ventral pigments absent. A central black pigment spot and a pair of dark brown pigments on pygidium. Pygidium has a dorsal notch and lacks appendages. Gastrotrochs on chaetigers III, V, VII, X, XIII, XV, XVII, XIX, XXI, and XXIII. Modified chaetae develop in chaetiger V in late larvae.

###### Remarks.

No adults of this species were collected in the present study. The 18S and 16S rRNA gene sequences obtained from the larvae of this species neither match nor constitute a monophyletic clade with any of the other available spionid sequences (Figs 2, 3). However, this species was tentatively identified as D. cf. commensalis based on its larval morphology, as it includes the characteristic dorsal pigment pattern of larvae of *D. commensalis* as described by Andrews (1891), Hatfield (1965), Blake (1969), and Radashevsky (1989) (described as *Polydora commensalis* by all of these authors). The combination of a slender body and a single dorsal median row of distinct melanophores from chaetiger I to the end of the body is distinctive among spionid larvae and has not been reported for any other spionid species. Currently, there are no records of *D. commensalis* from Japan; however, the presence of this species in Japan is expected as it has been reported from the Asian continental coast of the Sea of Japan and the Kurile Islands (Radashevsky 1993). This species is an obligate symbiont of hermit crabs (Blake 1996; Williams and McDermott 1997), but little effort was devoted to collecting hermit crab shells in the present study.

Notably, the results of the phylogenetic analysis in the present study showed that D. cf. commensalis deviates from the monophyletic clade constituted by many other *Dipolydora* species. This result supports the suggestion by Blake (1971) that *D. commensalis* may represent a distinct genus as its morphology deviates widely from other species of the genus *Polydora* and *Dipolydora*.

Only three individuals of planktonic larvae of this species were collected in Sasuhama in January 2013. A small patch of lateral black pigments on the anterior margin of each chaetiger in late larvae was described in Hatfield (1965) and Blake (1969). However, these pigments were not observed in the present study, as in Andrews (1891) and Radashevsky (1989). Although Hatfield (1965) and Blake (1966) noted the high similarity between the larval morphologies of *D. commensalis* and *Polydora hermaphroditica* Hannerz, 1956, the adult morphologies of these two species were reported to be completely different (Bhaud 1966). The dorsal pigment pattern of *P. hermaphroditica* larvae reported by Hannerz (1956) rather resembles those of *Polydora glycymerica* and Polydora cf. glymymerica larvae reported by Radashevsky (1989) and in the present study, respectively.

##### Dipolydora giardi

Taxon classificationAnimaliaSpionidaSpionidae

(Mesnil, 1896)

99D40B8E-097A-5D17-8BEA-769F3B90386C

Fig. 7M, N

###### Larval morphology.

Overall body shape elongated and slender. Prostomium small and rounded anteriorly. Three pairs of black eyes arranged in transverse row, most lateral pairs double‐eyes. Black pigmentation on lateral peristomium absent. Two pairs of dorsal black spots begin on chaetiger III onwards and continue to posterior end, sometime medial pair in first 2–4 chaetigers band‐shaped. Small medial spot of black pigment on posterior margin of each chaetiger usually from chaetigers III, rarely from V or VI, in late larvae. Two small spots of black pigmentation occur lateral to the medial black pigmentation from approximately chaetiger VI or VII. A small black pigment spot, not visible dorsally, present on antero‐lateral edges from chaetiger II onwards. Black pigment occurs on pygidium. Rust‐colored pigment occurs in pharynx. Ventral pigment absent. Some metamorphosing larvae reduce pigmentation over the entire body and present whitish appearance with eyes fused and appears as one pair (Fig. 7N). Gastrotrochs on chaetigers III, V, VII, X, XIII, and XV.

###### Remarks.

Adults of this species are shell-borers and were collected from shells of cultured *Mizuhopecten yessoensis* (Jay, 1857) (formerly as *Patinopecten yessoensis*) scallops suspended in Onagawa Bay in December 2010. Adult morphology agrees with the description of *D. giardi* by Sato‐Okoshi (1999). Therefore, this species was referred to *D. giardi*. The larvae and adults were confirmed to match using molecular data (Fig. 3).

The 18S rRNA gene sequences of this species are very similar to that of *D. capensis* 1PE from South Africa (KY677896) reported by Williams et al. (2017), but there is a slight difference between their sequences (0.12% difference: 2/1714 bp). It is unclear whether this difference indicates that these two are the same or different species because two different 18S rRNA gene sequences have been reported from South Africa and are currently under the same species name (*D. capensis*) (Table 2, Fig. 3). No gene sequences of D. cf. giardi previously recorded from South Africa (Simon 2011) are available.

Planktonic larvae of this species were collected from Onagawa Bay in December 2010, June, July, October, November, and December 2011, and December 2012, and from Sasuhama in January 2013. The larval morphology of this species was previously described from California by Day and Blake (1979, as *Polydora giardi*). The morphology and dorsal pigment pattern of late larvae described by these authors resembles that reported here, but there are slight differences: two golden pigment spots present on either side of chaetiger I in the former description but absent in the latter; two small lateral melanophores present on chaetigers I and II in the former description but absent in the latter; a medial black pigmentation beginning from chaetiger II onwards in the former description but from chaetiger III onwards in the latter description; and two small spots of black pigmentation lateral to the medial black pigmentation starting from chaetiger III in the former description but from more posteriorly in the latter description. These differences between specimens from Japan and California may indicate that they are different species, or that intraspecific variation occurs in larval dorsal pigmentation. Day and Blake (1979) pointed out differences in reproductive traits between the Californian and French populations and suggested the existence of two different species. Therefore, more than one species may be included under the name of *D. giardi*, which currently is reported with a worldwide distribution (Radashevsky and Petersen 2005).

##### Dipolydora cf. socialis

Taxon classificationAnimaliaSpionidaSpionidae

(Schmarda, 1861)

2A89B6F1-2882-5E15-B9B9-095DA6895234

Fig. 7O

###### Larval morphology.

Late larvae usually thick and slightly fusiform in shape, although not as much as the larvae of *Boccardia* sp. 2 (Fig. 7G), *Boccardiella hamata* (Fig. 7H, I), and *Pseudopolydora* species (Fig. 9A–I). Anterior margin of prostomium has yellow‐brown pigment. Three pairs of black eyes arranged in transverse row, most lateral pairs double‐eyes. Band of black pigment on each lateral part of the peristomium. First dorsal black melanophores occur as paired bands on chaetiger III and continue through to chaetiger V. From chaetiger VI, two pairs of dorsal black spots or bands occur and continue to posterior end of body. From chaetiger IV or V and continuing posteriorly, clusters of small black pigmented cells present in transverse row on dorsal posterior half of chaetigers. Lateral pigment found on late larvae on chaetiger II. Each notopodial lobe tipped with orange pigment, small patch of black pigment at the base of notopodial lobes. Ventral pigment consists of paired bars on posterior border of chaetigers, commencing with chaetiger II. Some black or brown pigment may occur on pygidium. Gastrotrochs occur on chaetigers III, V, VII, X, XIII, XV and XVII.

###### Remarks.

Adults of this species were non‐boring and collected from muddy bottom sediment at 22 m depth in Onagawa Bay in December 2010 by using a Smith‐McIntyre grab sampler and from bottom sediments of shallow subtidal zone in Sasuhama in April 2013. Adult morphology agrees with the description of *D. socialis* by Sato‐Okoshi (2000). The 18S rRNA gene sequence obtained in the present study showed a 0.35% (6/1715 bp) difference with that of D. cf. socialis from South Africa (KY677899) reported by Williams et al. (2017), which may indicate that these two are different species. The 18S rRNA gene sequence obtained in the present study rather closer to that of *D. carunculata* (940/942 bp match) reported by Radashevsky and Pankova (2013), but the 16S gene sequence showed a 2.3% (11/475 bp) difference with that of *D. carunculata*. As described above, since the taxonomic status of the species reported here is uncertain, we tentatively referred to it as D. cf. socialis.

Planktonic larvae of this species were collected from Onagawa Bay in November 2010 and 2011, and in October 2012. The larvae and adults were confirmed to match (18S: 1770/1770, 16S: 473/475 bp) using molecular data (Fig. 2). The larval morphology of this species agrees with that of *D. socialis* described as *Polydora socialis* by Blake (1969) and Carrasco (1976).

##### Dipolydora

Taxon classificationAnimaliaSpionidaSpionidae

sp.

3F43D586-7E08-56D8-A089-CBE49E1A084E

Fig. 7P

###### Larval morphology.

Overall body shape slender. Prostomium small and rounded anteriorly. Anterior margin of prostomium has weak yellow‐brown pigment. Three pairs of black eyes present in transverse row, most lateral pairs double‐eyes. Black pigmentation on lateral peristomium absent. A pair of dorsal black spots present on chaetiger III onwards. A small medial spot of black pigment on posterior margin of chaetiger III. Some black pigment occurs on pygidium. Ventral pigment absent. Gastrotrochs occur on chaetigers III and V.

###### Remarks.

No adult individuals of this species were collected in the present study. The 18S and 16S rRNA gene sequences obtained from larvae in the present study did not match any available *Dipolydora* sequences. As the larvae specimens formed a monophyletic clade with the other *Dipolydora* species (excluding *D. armata*, *D. capensis* 1GG, D. cf. commensalis, and *D. quadrilobata*) with robust statistical supports (Figs 2, 3), this species was referred to *Dipolydora* sp.

##### Genus Polydora

Taxon classificationAnimaliaSpionidaSpionidae

Bosc, 1802

0E42E1C1-4A0A-5058-816D-E65E604D4250

###### Larval diagnosis.

Overall shape slender or slightly fusiform. Prostomium broad or small and rounded anteriorly. Three pairs of black eyes present, most lateral pairs often double‐eyes. Some species have ramified melanophore between central and lateral pairs of eyes. Dorsal pigmentation usually consists of two rows of bands, spots, or branching melanophores in most species, while some species have a single row of mid‐dorsal melanophores (e.g., Polydora cf. glycymerica). Lateral and ventral pigments present or absent. Nototrochs occur in all chaetigers except first two. Gastrotrochs occur in irregular pattern. Modified chaetae develop on chaetiger V in late larvae (Wilson 1928; Thorson 1946; Hannerz 1956; Hopkins 1958; Woodwick 1960; Blake 1969; Carrasco 1976; Radashevsky 1986, 1988, 1989; 1994, 2005; Plate and Husemann 1994; Sato‐Okoshi 1994; Williams 2001; Radashevsky and Cárdenas 2004; Blake 2006; Radashevsky et al. 2006; Zhang et al. 2009; Gao et al. 2011; David et al. 2014; Barros et al. 2017; Blake 2017; Radashevsky and Migotto 2017; Ye et al. 2017).

#### Identification key to species of the larvae belonging to the genus *Polydora* in northeastern Japan

**Table:** 

1	Mid‐dorsal single row of branching melanophores present	**Polydora cf. glycymerica**
–	Mid‐dorsal single row of branching melanophores absent	**2**
2	Vestibule and pharynx with black pigmentation	***Polydora brevipalpa***
–	Vestibule and pharynx not pigmented with black	**3**
3	Dorsal melanophores on each chaetiger faint	***Polydora* sp. 2**
–	Dorsal melanophores on each chaetiger distinct	**4**
4	Black or brown pigmentation on lateral part of peristomium present	**5**
–	Black or brown pigmentation on lateral part of peristomium absent	**8**
5	Distinct ventral pigment spot (yellow‐green, brown, or black) present	***Polydora cornuta***
–	Distinct ventral pigment spot absent	**6**
6	Black pigmentation on lateral part of lateral peristomium present	***Polydora* sp. 3**
–	Brown pigmentation on lateral part of lateral peristomium present	**7**
7	Two rows of dorsal melanophores from chaetigers III–VI or VII band‐shaped, followed by large branching melanophores in posterior chaetiger	***Polydora onagawaensis***
–	Two rows of dorsal melanophores on anterior chaetiger dot‐like or short band‐shaped, followed by dot‐like not branching melanophores in posterior chaetiger	***Polydora* sp. 1**
8	Two rows of dorsal melanophores mostly band‐shaped with some of them slightly branching	**Polydora cf. spongicola**
–	Two rows of dorsal melanophores band‐shaped in anterior chaetigers, followed by branching melanophores in posterior chaetiger	**9**
9	Two rows of dorsal melanophores from chaetigers III–VII band‐shaped, followed by pairs of large branching melanophores	***Polydora hoplura***
–	Two rows of faint dorsal melanophores from chaetigers II–V or VII band‐shaped, followed by pairs of branching melanophores in posterior chaetigers or whole of dorsal surface covered by finely ramified black pigmentation	***Polydora* sp. 2**

##### Polydora brevipalpa

Taxon classificationAnimaliaSpionidaSpionidae

Zachs, 1933

538BB56E-FE4F-5441-8877-463B01FBB22D

Fig. 8A, B

###### Larval morphology.

**Figure 8.**
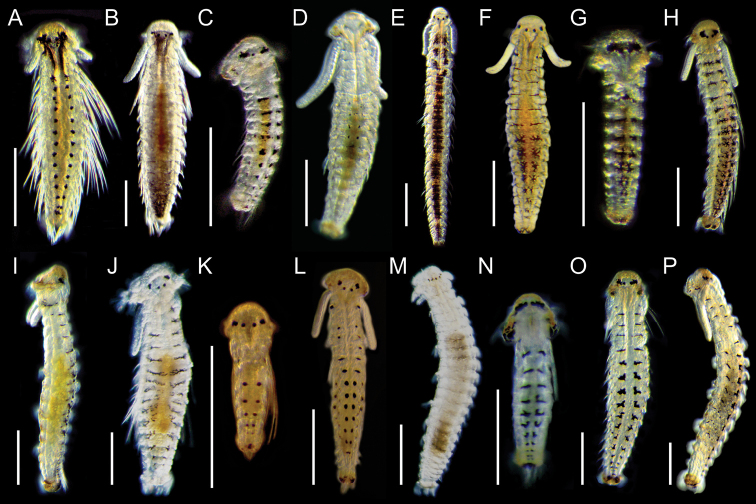
Light micrographs showing the morphologies of living spionid larvae of the genus *Polydora***A, B***Polydora brevipalpa*, dorsal view of 15‐chaetiger (**A**) and 17‐chaetiger larvae (**B**) **C, D***Polydora cornuta*, dorsolateral view of 11‐chaetiger larva (**C**) and ventral view of 17‐chaetiger larva (**D**) **E**Polydora cf. glycymerica, dorsal view of 25‐chaetiger larva **F***Polydora hoplura*, dorsal view of 15‐chaetiger larva **G–I***Polydora onagawaensis*, dorsal view of 10‐chaetiger (**G**) and 18‐chaetiger larvae (**H**), and lateral view of 16‐chaetiger larva (**I**) **J**Polydora cf. spongicola, dorsal view of 17‐chaetiger larva **K, L***Polydora* sp. 1, dorsal view of 7‐chaetiger (**K**) and 16‐chaetiger larvae (**L**) **M***Polydora* sp. 2, dorsal view of 23‐chaetiger larva **N–P***Polydora* sp. 3, dorsal view of 13‐chaetiger (**N**) and dorsal (**O**) and lateral view (**P**) of 18‐chaetiger larvae. Scale bars: 300 μm.

Overall shape slender and slightly fusiform. Prostomium broad and rounded anteriorly. Three pairs of black eyes present, innermost pair rounded, lateral pairs double‐eyes, ramified melanophore between innermost and lateral two pairs of eyes usually present. Black pigment on lateral peristomium absent. Dorsal pigmentation consists of two rows of melanophores from chaetiger III. Dorsal melanophores undergo expansion and contraction, may expand to branching melanophores or ramified appearance or covered almost whole of dorsal surface by very finely ramified black pigments (Fig. 8B), or they contract to dot‐like pigmentation patches (Fig. 8A). Lateral and ventral pigments absent. Vestibule and pharynx pigmented with black, gut pigmented with orange color. Modified chaetae develop on chaetiger V in late larvae. Gastrotrochs occur on chaetigers III, V, VII, X, XIII, XV, and XVII.

###### Remarks.

Adults of this species are boring and were collected from shells of cultured *M. yessoensis* scallops suspended in Onagawa Bay in February 2011 and Mutsu Bay in October 2011. This species was identified as *P. brevipalpa* as adult morphology agrees with the descriptions by Sato‐Okoshi (1999) and Sato‐Okoshi and Abe (2012). The larvae and adults were confirmed to 100% match using molecular data (Fig. 2).

Planktonic larvae of this species were collected from Onagawa Bay in April, May, and July 2011 and from Sasuhama in April 2011. The pair of large ramified or dot‐like melanophores from chaetiger III distinguishes larvae of this species from those of other *Polydora* species. Blake (2017) reported similar dorsal pigment patterns in the larvae of *Polydora spongicola* Berkeley & Berkeley, 1950. However, the larvae of *P. spongicola* in Blake (2017) differ from those of *P. brevipalpa* in having dorsal melanophores from chaetiger II instead of chaetiger III, dark green colored intestine instead of orange, and non‐pigmented pharynx instead of pigmented with black. Reproduction and life history of this species was reported in Sato‐Okoshi et al. (1990) and Sato‐Okoshi (1994) (both as *P. variegata*).

##### Polydora cornuta

Taxon classificationAnimaliaSpionidaSpionidae

Bosc, 1802

26251CE9-E6CE-56B1-A758-B2354C270278

Fig. 8C, D

###### Larval morphology.

Overall shape slender. Prostomium broad and rounded anteriorly. Three pairs of black eyes present, median pair rounded, most lateral pairs double‐eyes, ramified melanophores between first median and the second lateral pair of eyes usually present. In late larval stage, anterior part of prostomium and lateral lips of peristomium pigmented yellow or brown. Small spots of black pigments occur on lateral parts of peristomium. Dorsal pigmentation consists of two rows of melanophores from chaetiger III with those of anterior four chaetigers band‐shaped and then replaced by rounded or ramified melanophores from chaetiger VII onwards. Three rows of small faint dorsal spots of brown pigment present on posterior edge from chaetigers III or IV onwards in late larvae. Lateral pigment on chaetigers II, III, and often VI–XI extensive compared to that on other chaetigers. Large yellow or brown chromatophores occur ventrally from chaetigers V or VI onwards, usually three chromatophores arranged in transverse line except on gastrotroch‐bearing chaetigers where single midventral chromatophores present. Black pigment spots occur on ventral side of body (Fig. 8D) and mid‐dorsal part on pygidium (Fig. 8C). Gastrotrochs occur on chaetigers III, V, VII, X, XIII, XV and XVII.

###### Remarks.

Adults of this species were non‐boring and collected from mud deposits in crevices of shells of living *C. gigas* oysters in Sasuhama in June 2011 and from intertidal bottom sediment in Gamo Lagoon in August 2012. This species was identified as *P. cornuta* as adult morphology agrees with the description by Sato‐Okoshi (2000) and Radashevsky (2005). The larvae and adults were confirmed to match (18S: 1770/1770, 16S: 468/470 bp) using molecular data (Fig. 2).

Rice et al. (2008) suggested that at least three sibling species may be involved in North America under the name of *P. cornuta* by differences of mitochondrial COI sequences between California, Florida, and Maine populations. Takata et al. (2011) reported that the *P. cornuta* from Fukuyama in the Seto Inland Sea, western Japan is genetically close with the California/New Zealand lineage. It is unclear to which lineage the eastern Japan populations belong. The 18S rRNA gene sequence obtained in the present study showed a 1.9% (5/421 bp) difference with that of *P. cornuta* from Netherlands (KC686637).

Planktonic larvae of this species were collected from Gamo Lagoon in August 2012. The larval morphology of this species generally agrees with the descriptions of *P. cornuta* by Hannerz (1956, as *P. ligni*), Blake (1969, as *P. ligni*), Plate and Husemann (1994, as *P. ligni*), and Radashevsky (2005). Peristomial melanophores, which were reported by Hannerz (1956) and Blake (1969) but not by Radashevsky (2005), and middorsal vesiculate melanophores, which were reported by Radashevsky (2005) but not described by Hannerz (1956) and Blake (1969), were both present in specimens of the present study. Ventral pigmentation pattern was consistent with the description by Blake (1969) and Radashevsky (2005) instead of Hannerz’s (1956) description. The larval dorsal pigmentation pattern, similar to that of *P. cornuta*, is typically found in many other *Polydora* species. This species can, however, be distinguished by the characteristic ventral yellow pigmentation pattern as the yellow pigment on the ventral side of the other *Polydora* species is diffusely scattered and does not appear regularly arranged when present (Radashevsky 2005).

##### Polydora cf. glycymerica

Taxon classificationAnimaliaSpionidaSpionidae

Radashevsky, 1993

11AAF805-9059-5BDB-9291-68C8E930BDD2

Fig. 8E

###### Larval morphology.

Overall shape elongated and slender. Prostomium small and rounded anteriorly. Three pairs of black eyes present, most lateral pairs double‐eyes. Ramified melanophores between middle and lateral pair of eyes absent. Pigmentation on lateral peristomium absent. Two rows of ramified melanophores on chaetigers III–VI, and a median row of ramified melanophores from chaetiger VII onwards. Lateral and ventral pigments absent. A pair of black pigments occur on pygidium. Pygidium has a dorsal notch and lacks appendages. Gastrotrochs absent in 25‐chaetiger larvae, probably already lost. Modified chaetae develop on chaetiger V.

###### Remarks.

No adult individuals of this species were collected in the present study. The 18S and 16S rRNA gene sequences obtained from larvae in the present study did not match any of the available *Polydora* sequences. However, as the larvae formed a robustly supported clade with other *Polydora* species (Figs 2, 3), this species was referred to as the genus *Polydora*. Furthermore, the larval morphology including the characteristic dorsal pigment pattern of this larvae matches that of the larvae of *P. glycymerica* described by Radashevsky (1989). Therefore, this larva was tentatively identified as P. cf. glycymerica. However, there were slight differences between the present specimens and Radashevsky (1989) description: the two rows of ramified melanophores continued until chaetiger VI in the present description, whereas it continues to chaetigers VII–X according to Radashevsky (1989); ramified melanophores between the middle and lateral pair of eyes are present in the former description while absent in the latter; larvae of P. cf. glycymerica collected in the present study were 25‐chaetigers with > 2.0 mm long (Fig. 8E), whereas the largest larva observed by Radashevsky (1989) was a 20‐chaetiger specimen 1.8 mm long. Further studies should test whether these differences are attributable to individual or developmental variabilities or interspecific differences.

The dorsal median single row of ramified melanophores is distinct in the larvae of the genus *Polydora*. The larvae of *Polydora hermaphroditica* also have a dorsal median row of ramified melanophores such as that of the larvae of P. cf. glycymerica and *P. glycymerica* (Hannerz 1956; Plate and Husemann 1994). However, the first species differs from the other two by the absence of two rows of ramified melanophores on anterior chaetigers.

Only one individual of planktonic larva of P. cf. glycymerica was collected in Onagawa Bay in October 2011. *Polydora glycymerica* was previously recorded as a shell-borer of *Macridiscus aequilatera* (G. B. Sowerby I, 1825) from Oarai, Japan (Sato‐Okoshi 1999).

##### Polydora hoplura

Taxon classificationAnimaliaSpionidaSpionidae

Claparède, 1868

16E9ADF2-76BE-5E22-A402-AECF2ADE5A1A

Fig. 8F

###### Larval morphology.

Overall body shape slender or somewhat fusiform. Prostomium broad and rounded anteriorly. Three pairs of black eyes present, most lateral pairs double‐eyes. Ramified melanophores between first and second innermost pair of eyes absent. Black pigmentation patches on lateral peristomium absent. Dorsal pigmentation consists of two rows of melanophores from chaetiger III with those of first five pairs band‐shaped and then replaced by ramified melanophores in posterior chaetigers. Lateral pigments found on late larvae on chaetigers II–IV. Dorsolateral pigments at base of the parapodia start from chaetiger VII. A pair of black pigment occur on pygidium. Ventral pigment absent. Modified chaetae develop in chaetiger V in late larvae. Gastrotrochs occur on chaetigers III, V, VII, X, XIII, and XV.

###### Remarks.

This species is a shell-borer, and adult specimens were collected from the turban snail *Omphalius rusticus* (Gmelin, 1791) in Gobu‐ura and Onagawa Bay. This species was identified as *P. hoplura* as its adult morphology agrees with descriptions by Sato‐Okoshi and Abe (2012, as *P. uncinata*) and Sato‐Okoshi et al. (2017). The larvae and adults were confirmed to match (18S: 1769/1769, 16S: 464/475 bp) using molecular data (Fig. 2).

Only late larvae were found in July in Onagawa Bay. The larval morphology of this species agrees with descriptions by Wilson (1928) and Radashevsky and Migotto (2017). This species has adelphophagic and lecithotrophic larval development, in which larvae feed on nurse eggs in brood capsules, hatch at a very late stage, and have only a short pelagic life (Wilson 1928; Read 1975; Sato‐Okoshi et al. 2008, as *P. uncinata*; Radashevsky and Migotto 2017). The poecilogenous development of this species with planktotrophic and adelphophagic planktonic larvae was reported by David et al. (2014), David and Simon (2014), and Simon (2015).

##### Polydora onagawaensis

Taxon classificationAnimaliaSpionidaSpionidae

Teramoto, Sato‐Okoshi, Abe, Nishitani & Endo, 2013

DE344AA5-07ED-55FC-824B-1FABBB29E893

Fig. 8G–I

###### Larval morphology.

Overall body shape slender. Prostomium slightly broad and rounded anteriorly. Three pairs of black eyes present; median pair of eyes rounded, most lateral pairs double‐eyes. Ramified melanophore between middle and lateral pair of eyes usually present (Fig. 8G, H). Weak brown pigmentation located on lateral parts of peristomium, behind prototroch, occasionally much paler or absent. Dorsal pigmentation consists of two rows of melanophores from chaetiger III with those of first IV–VI band‐shaped and subsequently replaced by ramified melanophores. These melanophores undergo expansion and contraction. Lateral pigment found on chaetigers II and III in late larvae (Fig. 3G). Dorsolateral pigment at base of most parapodia, often appears to coalesce with dorsal pigment bands on anterior part of body (Fig. 8H). Pygidium has a dorsal notch and lacks appendages; a pair of black pigment patches occur on pygidium. Ventral brown pigment may be present on posterior part of late larvae (Fig. 8I). Telotroch well developed. Gastrotrochs on chaetigers III, V, VII, X, XIII, and XV; those of chaetigers III and V lost in late larvae (Fig. 8I). In late larvae, modified chaetae develop in chaetiger V.

###### Remarks.

This species is a shell-borer, and adult individuals were collected from shells of the wild turban snail *O. rusticus*, cultured scallop *M. yessoensis*, and wild and cultured *C. gigas* oysters in Onagawa Bay and Sasuhama, northeastern Japan. This species was identified as *P. onagawaensis* as adult morphology agrees with the description by Teramoto et al. (2013). The larvae and adults were confirmed to match (18S: 1771/1771, 16S: 472/473 bp) using molecular data (Fig. 2).

Planktonic larvae of this species were abundant from November to June in Onagawa Bay during the study period. The larval morphology of this species is similar to that of *Polydora* sp. 3 (see below). However, the former species has weak brown pigmentation on the lateral parts of the peristomium, whereas the latter species has large patches of black pigment on this region.

##### Polydora cf. spongicola

Taxon classificationAnimaliaSpionidaSpionidae

Berkley & Berkeley, 1950

D1B4DED1-354B-5D65-A806-AE070A1F6625

Fig. 8J

###### Larval morphology.

Overall body shape slender and slightly fusiform. Prostomium broad and rounded anteriorly. Three pairs of black eyes present; median eyes rounded, most lateral pairs double‐eyes. Ramified melanophores between middle and lateral pair of eyes absent. Black pigment on lateral peristomium absent. Dorsal pigmentation consists of two rows of band‐shaped melanophores from chaetiger II. These melanophores undergo expansion and contraction, expand to ramified melanophores or contract to non‐ramified band‐shaped melanophores. Lateral and ventral pigments absent. In late larvae modified chaetae develop in chaetiger V. Gastrotrochs on chaetigers III, V, VII, X, XIII, and XV.

###### Remarks.

Adults of this species were collected from mud tubes constructed on the sponge *Mycale* sp. in Moroiso Bay, Misaki Peninsula (Table 1). The morphology of its modified spines in chaetiger V and the sponge‐associated ecology of adults match the description of *P. spongicola* by Radashevsky (1993). However, this species was referred to P. cf. spongicola because the adult specimens were in poor condition, which hindered their morphology examination. The larvae and adults were confirmed to match (18S: 1770/1771, 16S: 474/475 bp) using molecular data (Fig. 2).

Only one planktonic larva of this species was collected in Sasuhama in January 2013. The larval morphology of P. cf. spongicola closely resembles that of *P. spongicola* described by Radashevsky (1988, as *Polydora uschakovi* Buzhinskaja, 1971) from Russia. *Polydora uschakovi* originally described from Russia was synonymized with *P. spongicola* (type locality: Canada) by Radashevsky (1993). Later, Blake (2017) described the larvae of *P. spongicola* from California and doubted this synonymization because, despite the similarities between the larvae from Russian and California, there are several morphological differences including the nature of the major spines of chaetiger V and the distribution of nototrochs and gastrotrichs. However, the larval dorsal pigment pattern of *P. spongicola* described by Blake (2017) greatly differs from those of P. cf. spongicola in the present study and of *P. spongicola* in Radashevsky (1988) but resembles that of *P. brevipalpa* in the present study. Conspecificity between *P. uschakovi* and *P. spongicola* should be verified in future studies.

##### Polydora

Taxon classificationAnimaliaSpionidaSpionidae

sp. 1

D62F9D9F-0771-52DA-A400-777FC21CD179

Fig. 8K, L

###### Larval morphology.

Overall body shape slender. Prostomium broad and rounded anteriorly. Three pairs of black eyes present; median eyes rounded and lateral pairs double‐eyes. Ramified melanophore between innermost and next to innermost pairs of eyes absent. Weak brown pigmentation on lateral parts of peristomium present or absent. Dorsal pigmentation consists of two rows of melanophores from chaetiger III, those of first five pairs band‐shaped and remaining pairs dot‐like in late larvae (Fig. 8L). These melanophores all dot‐like in early larvae (Fig. 8K). Lateral pigment found on chaetigers II, IX, X, and XI in late larvae. Dorsolateral pigment at base of parapodia on posterior chaetigers. A pair of black and brown pigment patches occur on pygidium. Ventral brown pigment present on posterior part of late larvae. Pygidium has a dorsal notch and lacks appendages. Telotroch well developed. In late larvae, modified chaetae develop in 5^th^ chaetiger.

###### Remarks.

Adults of this species are shell-borer and were collected from the shell of the turban snail *O. rusticus* in Sasuhama. The adults of this species have characteristic conspicuous black bars in their palps and are morphologically similar to *Polydora neocaeca* Williams & Radashevsky, 1999. *Polydora haswelli* previously recorded in Japan (Sato-Okoshi and Abe 2013) was reexamined as *P. neocaeca* by comparing morphology and molecular sequences with the specimens from near the type locality (Malan et al. 2020). As the 18S and 16S rRNA gene sequences of *Polydora* sp. 1 and *P. neocaeca* showed differences (18S: 8/1771, 16S: 40/476 bp), the specimens collected in the present study were referred to a different species. Only two individuals of planktonic larvae of this species were collected in Onagawa Bay in April and July 2011. The larvae and adults were confirmed to 100% match using molecular data (Fig. 2).

##### Polydora

Taxon classificationAnimaliaSpionidaSpionidae

sp. 2

09350C81-5DD7-56DF-9731-78DC5133EAA4

Fig. 8M

###### Larval morphology.

Overall body shape slender. Prostomium broad and rounded anteriorly. Three pairs of black eyes present, most lateral pairs double‐eyes. Ramified melanophores between first and second innermost pairs of eyes absent. Pigmentation on lateral peristomium weak brown or absent. Dorsal pigmentation consists of two rows of melanophores from chaetiger II, with those of first 4–6 chaetigers being band‐shaped and then replaced by ramified melanophores in posterior chaetigers. Dorsal pigments faint, undergo expansion and contraction, expand to cover almost whole of dorsal surface as finely ramified black pigmentation (Fig. 8M) or contract to band‐shaped or dot‐like black pigments without ramification. Faint lateral pigment found on late larvae on chaetigers VII onwards. Ventral pigments absent. A pair of brown pigments occur on the pygidium. Pygidium has a dorsal notch and lacks appendages. Gastrotrochs on chaetigers III, V, VII, X, XIII, XV, XVII, and XIX. Modified chaetae develop in chaetiger V in late larvae.

###### Remarks.

No benthic adult stages were collected in the present study. These larvae formed a robustly supported monophyletic clade with other *Polydora* species (Figs 2, 3). Nevertheless, this species was identified as a member of *Polydora*. As the 18S and 16S rRNA gene sequences obtained from the larvae did not match any other available *Polydora* sequences, this species was referred to *Polydora* sp. 2.

Only two individuals of planktonic larvae of this species were collected from Sasuhama and Gamo Lagoon in January 2013. The faint dorsal pigmentation of the larvae of this species is unique among the members of *Polydora* collected in the present study.

##### Polydora

Taxon classificationAnimaliaSpionidaSpionidae

sp. 3

E8C2D314-9FF1-52FC-B7A9-D1EA2183221A

Fig. 8N–P

###### Larval morphology.

Overall body shape slender. Prostomium broad and rounded anteriorly. Three pairs of black eyes present; median pair of eyes rounded, most lateral pairs double‐eyes, ramified melanophore between innermost and next to innermost pairs of eyes present. Large patches of black pigment located on lateral part of peristomium, behind prototroch. Dorsal pigment pattern consists of two rows of melanophores from chaetiger III with those of first four or five chaetigers being band‐shaped and then replaced by ramified branching melanophores (Fig. 8O). These melanophores undergo expansion and contraction. Lateral pigment found on chaetigers II–IV, resumes again from chaetiger VII in late larvae (Fig. 8P). A pair of black pigment patches occur on pygidium. Ventral brown and black pigment present on posterior part in late larvae ready to metamorphose. Pygidium has a dorsal notch and lacks appendages. Telotroch well developed. Gastrotrochs on chaetigers III, V, VII, IX, X, XIII, XV, and XVII, lost on chaetigers III and V in late larvae (Fig. 8P). In late larvae, modified chaetae develop in chaetiger V.

###### Remarks.

No benthic adult stages were collected in the present study. The 18S rRNA gene sequences obtained from the larvae did not match any available *Polydora* sequences. As the larvae formed a robustly supported monophyletic clade with other *Polydora* species (Figs 2, 3), this species was referred to *Polydora* sp. 3.

Planktonic larvae of this species were collected from December to June in Onagawa Bay every year during the study period. Planktonic larvae of this species were previously reported to be abundant in Onagawa Bay in the winter season from December to March (Abe et al. 2014, as *Polydora* sp.). Large patches of black pigment on the lateral peristomium are the main characteristic of this species and differentiate it from the other species of the genus observed in the present study, even at early planktonic stages (Fig. 8N).

##### Genus Pseudopolydora

Taxon classificationAnimaliaSpionidaSpionidae

Czerniavsky, 1881

4D1DED37-FA9A-5668-BDD3-C30FCDC38068

###### Larval diagnosis.

Overall body shape thick and fusiform. Prostomium broad and rounded or gently notched anteriorly. Three pairs of black eyes present, most lateral often double‐eyes. Mid‐dorsal melanophore on the first chaetiger present in many species, absent in some species. Dorsal pigmentation consists of one or two pairs of branching melanophores (except *P. rosebelae*: mid‐dorsal single row of melanophores present). Lateral and ventral pigments present or absent. Nototrochs occur in all chaetigers except first two chaetigers. Gastrotrochs occur in irregular pattern. Modified chaetae in chaetiger V and ventral hooded hooks from chaetiger VIII onwards develop in late larvae (Hannerz 1956, as *Polydora*; Rullier 1963, as *Polydora*; Rasmussen 1973; Blake and Woodwick 1975; Srikrishnadhas and Ramamoorthi 1977; Wu and Chen 1980; Radashevsky 1983, 1985; Plate and Husemann 1994, as *Polydora*; Hsieh 1994; Blake 2006; Radashevsky and Migotto 2009; Kondoh et al. 2017).

#### Identification key to species of the larvae belonging to the genus *Pseudopolydora* in northeastern Japan

**Table:** 

1	A pair of dorsal melanophores on each chaetigers	**2**
–	Two pairs of dorsal melanophores on each chaetigers.	**4**
2	A pair of dorsal melanophores lack ramification; three pairs of black eyes are arranging more or less a straight line	***Pseudopolydora paucibranchiata***
–	A pair of dorsal melanophores greatly ramified; lateral and anterior pairs of eyes link each other and form dumbbell‐shaped eyes	**3**
3	Ramification of dorsal melanophores covering most of dorsal side; a conspicuous large black pigment on pygidium	***Pseudopolydora tsubaki***
–	Ramification of dorsal melanophores not covering most of dorsal side; a conspicuous black pigment spot on pygidium	***Pseudopolydora* sp.**
4	A central pair of dorsal black pigment “tilted wheels” shaped in anterior chaetigers; a weak mid‐dorsal pigment present from chaetiger VI	**Pseudopolydora aff. achaeta**
–	A central pair of dorsal melanophore dot‐like or ramified; mid‐dorsal melanophores absent except the first chaetiger	**5**
5	Distinct ramified mid‐dorsal melanophore present on first chaetiger	**Pseudopolydora cf. reticulata**
–	Mid‐dorsal melanophore on first chaetiger absent or not distinct and not ramified	**Pseudopolydora cf. kempi**

##### Pseudopolydora aff. achaeta

Taxon classificationAnimaliaSpionidaSpionidae

Radashevsky & Hsieh, 2000

EA54BA8B-CB07-5708-868F-B9FD99EC11C7

Fig. 9A, B

###### Larval morphology.

Overall body shape fusiform, head region enlarged due to broad prostomium and expanded lateral lips of vestibule. Prostomium gently notched anteriorly. Three pairs of black eyes present in more or less a straight line, most lateral pairs double eyes. Mid‐dorsal melanophore on first chaetiger present. Dorsal pigmentation consists of two pairs of lateral and central rows of melanophores. Lateral ones dot‐like, beginning on chaetiger II. Central ones shaped like “tilted‐wheels” (inverted v-shape) begin on chaetiger III. A central pair of dorsal pigment patches gradually become dot‐like on posterior chaetiger. Weak mid‐dorsal pigments occur from chaetiger VI. Two medial black pigmentation areas occasionally present ventrally, on approximately chaetiger VI and anterior margin of pygidium. Anterior and posterior margin of prostomium have considerable brown pigment. Black pigment spots occur on sides of prostomium and peristomium. Pygidium has a central black pigment spot. Gastrotrochs on chaetiger III, V, VII, and XII in 13‐chaetiger larvae.

**Figure 9.**
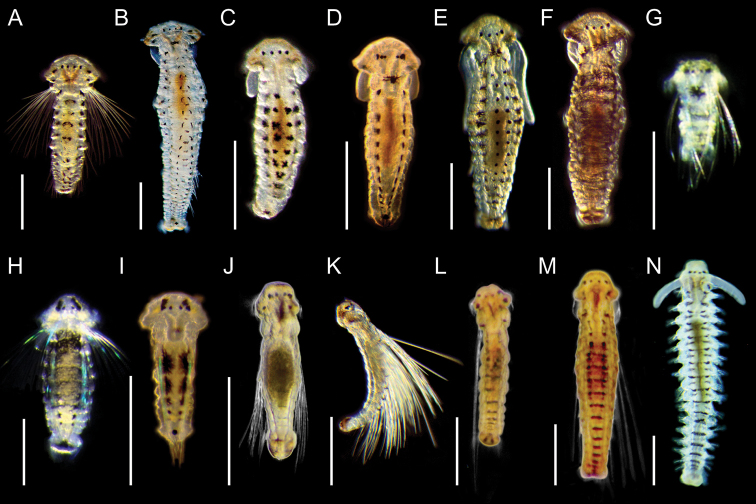
Light micrographs showing the morphologies of living spionid larvae of genera *Pseudopolydora* and *Spio***A, B**Pseudopolydora aff. achaeta, dorsal view of 12‐chaetiger (**A**) and 25‐chetiger larvae (**B**) **C**Pseudopolydora cf. kempi, dorsolateral view of 12‐chetiger larva **D***Pseudopolydora paucibranchiata*, dorsal view of 13‐chaetiger larva **E, F**Pseudopolydora cf. reticulata, dorsal view of 17‐chaetiger (**E**) and 16‐chaetiger larvae (**F**) **G, H***Pseudopolydora tsubaki*, dorsal view of 5‐chaetiger (**G**) and 11‐chaetiger larvae (**H**) **I***Pseudopolydora* sp., dorsal view of 7‐chaetiger larva **J, K***Spio* sp. 1, dorsal view of 8‐chaetiger (**J**) and lateral view of 12‐chaetiger larvae (**K**) **L–N***Spio* sp. 2, dorsal view of 10‐chaetiger (**L**) and 17‐chaetiger larvae (**M**), and 17‐chaetiger metamorphosing larvae (**N**). Scale bars: 300 μm.

###### Remarks.

Adult individuals of this species were collected from muddy bottom sediments at 22 m depth in Onagawa Bay in December 2010 and September and December 2011 by using a Smith‐McIntyre or Ekman‐Birge grab sampler. Adult morphology agrees with the descriptions of *P. achaeta* by Radashevsky and Hsieh (2000) and Abe et al. (2016). However, the 16S rRNA gene sequence obtained in the present study showed a 11.5% (35/304 bp) difference with that of *P. achaeta* from Taiwan (country of type locality), which indicate that these two are different species. Therefore, this species is referred to P. aff. achaeta. The larvae and adults were confirmed to 100% match using molecular data (Fig. 2).

Planktonic larvae of this species with more than 3‐chaetiger stages were abundant in Onagawa Bay during July to November (Abe et al. 2014). A dorsal pigmentation area shaped like “tilted wheels” is a unique characteristic of this species among the known *Pseudopolydora* larvae.

##### Pseudopolydora cf. kempi

Taxon classificationAnimaliaSpionidaSpionidae

(Southern, 1921)

16EC9D4B-3EC8-548E-96F2-57625D52BB4B

Fig. 9C

###### Larval morphology.

Overall body shape fusiform, head region enlarged due to broad prostomium and expanded lateral lips of vestibule. Prostomium gently notched anteriorly. Three pairs of black eyes present in more or less a straight line, most lateral pairs double‐eyes. Mid‐dorsal melanophore on first chaetiger usually absent (Fig. 9C), small non‐ramified melanophore present in some individuals. Dorsal pigment consists of four rows of lateral and central pairs of pigment spots. Lateral and central pigments usually begin from chaetigers II and III, respectively. There pigment spots undergo expansion and contraction. Ventral pigment begins on chaetiger III, consists of paired bars on posterior border of each chaetiger. Anterior and posterior margin of prostomium have considerable brown pigment. Black pigment spots occur on sides of peristomium. Pygidium has black central spot. Gastrotrochs on chaetigers V and VII in 13‐chaetiger larvae.

###### Remarks.

Adult individuals of this species were collected from muddy sediment in Gamo Lagoon in January, May, and December 2011, and April 2013. Adult morphology agrees with the description of P. cf. kempi by Abe et al. (2016). Therefore, these individuals were referred to P. cf. kempi. The 16S rRNA gene sequence obtained in the present study showed a 99.7% (305/306 bp) similarity with that of *P. kempi japonica* Imajima & Hartman, 1964 from Russia (MG460897) reported by Radashevsky et al. (2020b), indicating these two are same species. It will need to be clarified whether *P. kempi* (type locality India) and subspecies *P. kempi japonica* (type locality Japan) are the same species. The larvae and adults were confirmed to 100% match using molecular data (Fig. 2).

Planktonic larvae of this species larger than 12‐chaetiger stages were collected from Gamo Lagoon in August 2012. The larval morphology of this species observed in the present study agrees with the descriptions of *P. kempi* by Blake and Woodwick (1975) and of P. cf. kempi by Kondoh et al. (2017). These species have adelphophagic and lecithotrophic larval development, in which larvae feed on nurse eggs in brood capsules, hatch at a very late stage, and have a short pelagic life (Blake and Woodwick 1975; Kondoh et al. 2017). Reproduction and larval development of these species under the name of *P. kempi* and *P. kempi japonica* were also described by Srikrishnadhas and Ramamoorthi (1977), Myohara (1979), and Radashevsky (1985). However, the larvae of species in these descriptions resemble those of Pseudopolydora cf. reticulata Radashevsky & Hsieh, 2000 described by Kondoh et al. (2017) and of the present study in having planktotrophic development without nurse eggs and distinct dorsal melanophores including a middorsal melanophore on the first chaetiger. The taxonomy of *P. kempi* is unclear because its original description is quite brief, and the current location of type specimen is unknown (Radashevsky and Hsieh 2000). Therefore, studies resolving the taxonomy of *P. kempi* are necessary.

##### Pseudopolydora paucibranchiata

Taxon classificationAnimaliaSpionidaSpionidae

(Okuda, 1937)

A7B847FC-A7B4-5BF0-8233-191F99289062

Fig. 9D

###### Larval morphology.

Overall body shape fusiform, head region enlarged due to broad prostomium and expanded lateral lips of vestibule. Prostomium gently notched anteriorly. Three pairs of black eyes present in more or less a straight line, most lateral pair comma‐shaped. A mid‐dorsal ramified melanophore on chaetiger I. A pair of melanophores present dorso‐laterally from chaetigers II onwards. Black pigment spots occur on lateral surface of chaetiger II, on sides of peristomium, and pygidium. Two small medial black pigment spots occasionally present ventrally on approximately chaetiger VI and anterior margin of pygidium. Gut has yellow‐green color due to ingested food. Gastrotrochs on chaetiger V, VII, and XI in 13‐chaetiger larvae.

###### Remarks.

Adult individuals were collected from muddy bottom sediment in the intertidal zone of Mangoku‐ura Inlet in July 2014. Adult morphology agrees with the description of *Pseudopolydora paucibranchiata* by Okuda (1937, as *Polydora*). Therefore, these individuals were referred to this species. The larvae and adults were confirmed matching (18S: 1784/1784, 16S: 454/455 bp) using molecular data (Fig. 2).

The planktonic larvae of this species were reported to be common in Onagawa Bay during June to November (Abe et al. 2014). The larval morphology of this species observed in the present study agrees with the descriptions by Blake and Woodwick (1975), Ward (1977), Myohara (1980), Wu and Chen (1980), Radashevsky (1983), and Blake (2006). The dorsal pigment pattern of this species consists of one pair of melanophores, which agrees with that of the larvae of *Pseudopolydora vexillosa* Radashevsky & Hsieh, 2000 photographed by Mok et al. (2009) and Chandramouli et al. (2011, 2013), currently synonymized to *P. paucibranchiata* (Junqueira et al. 2009). The dorsal pigment pattern of these larvae is also similar to that of the larvae of *Pseudopolydora antennata* (Claparède, 1869) described by Hannerz (1956, as *Polydora antennata*), but the latter species has a more thickened body shape compared to the former.

##### Pseudopolydora cf. reticulata

Taxon classificationAnimaliaSpionidaSpionidae

Radashevsky & Hsieh, 2000

CD74A6AC-C684-50D4-AE55-9A609DDDA1E4

Fig. 9E, F

###### Larval morphology.

Overall larval shape fusiform, head region enlarged due to broad prostomium and expanded lateral lips of vestibule. Prostomium slightly notched anteriorly. Three pairs of black eyes present in more or less a straight line, most lateral pairs double‐eyes. Large patches of black pigment on lateral peristomium present. Mid‐dorsal melanophore on chaetiger I usually present. Dorsal pigments undergo expansion and contraction, expanding to cover almost complete dorsal surface with finely ramified black pigment (Fig. 9F) or contract to dot‐like black pigmentation without ramifications (Fig. 9E). Ventral pigment usually absent, consisting of paired bars on the posterior border on anterior chaetigers occasionally present. Black pigment on pygidium. Gastrotrochs on chaetigers V, VII, and XII in 17‐ and 18‐chaetiger larvae, late larvae lose gastrotrochs on chaetigers V and/or XXII.

###### Remarks.

Adult individuals of this species were collected from muddy sediment in Gamo Lagoon in April 2013 and Sasuhama in July and September in 2011. Adult morphology agrees with the description of P. cf. reticulata by Abe et al. (2016). Therefore, these individuals were referred to this species. The 16S rRNA gene sequence obtained in the present study showed a 99.4% (304/306 bp) similarity with that of *P. bassarginensis* (Zachs, 1933) from Russia (MG460894) reported by Radashevsky et al. (2020b), indicating these two are one species. Although the Japanese population shows intermediate morphological characteristics between *P. reticulata* (type locality Taiwan) and *P. bassarginensis* (type locality Russia), Abe et al. (2016) tentatively identified the Japanese population as P. cf. reticulata because the original description of *P. bassarginensis* is very brief and the status of the species remains unclear. The results of the present study indicate that the Japanese population likely belongs to *P. bassarginensis*, but whether the morphologically similar *P. reticulata* and *P. bassarginensis* are considered molecularly as the same or different species will need to be clarified. Planktonic larvae of P. cf. reticulata larger than the 3-chaetiger stage were collected from Gamo Lagoon, Sasuhama, and Onagawa Bay mainly from July to September. The larvae and adults were confirmed to match (18S: 1775/1775, 16S: 468/470 bp) using molecular data (Fig. 2).

Pseudopolydora cf. reticulata and P. cf. kempi are very similar sister species; specimens from Japan once misidentified as P. cf. kempi or *P. kempi japonica* were distinguished based on their morphology and 18S and 28S rRNA gene sequences by Abe et al. (2016). The larvae of these two species are also quite similar, but the mid‐dorsal melanophore on chaetiger I is usually present in Ps. cf. reticulata and absent in P. cf. kempi; moreover, the dorsal pigmentation is more distinct in the former species than in the latter. The two species also differ in reproduction and larval development: P. cf. kempi has lecithotrophic development with a short planktonic phase, whereas P. cf. reticulata has planktotrophic development with a long planktonic phase (Kondoh et al. 2017).

##### Pseudopolydora tsubaki

Taxon classificationAnimaliaSpionidaSpionidae

Simon, Sato‐Okoshi & Abe, 2017

3170EFC7-7FFB-56E1-A64C-B53FB9BCF226

Fig. 9G, H

###### Larval morphology.

Overall larval shape fusiform, head region enlarged due to broad prostomium and expanded lateral lips of vestibule. Prostomium gently notched anteriorly. Three pairs of black eyes present, comprising one pair of rounded median eyes, one pair of large lateral eyes, and one pair of large anterior eyes. Lateral and anterior pairs of eyes link with each other and form dumbbell‐shapes almost divided into two equal parts by a deep constriction. Mid‐dorsal ramified melanophore present on chaetiger I in early larvae. Mid‐dorsal melanophore on chaetiger I occasionally absent or expanded to finely ramified melanophore in late larvae. A paired of melanophores occur dorso‐laterally from chaetiger II onwards, usually finely ramified in late larvae (Fig. 9H). Ramified melanophores cover almost entire ventral surface on chaetigers III–VII in 11‐chaetiger larvae. Black pigment spots on sides of peristomium absent. Conspicuous large black pigment on pygidium. Gastrotrochs on chaetigers V and VII in 11‐chaetiger larvae.

###### Remarks.

Adult individuals were collected from mud deposits in crevices of shells of living *C. gigas* oysters in Habu Port, Izu‐Oshima Island, and Tomiura, Boso Peninsula in April 2016. Adult morphology agrees with the description of *Pseudopolydora tsubaki* by Simon et al. (2019a). Therefore, these individuals were identified as *P. tsubaki*. The larvae and adults were confirmed to 100% match using molecular data (Fig. 2).

A small number of planktonic larvae of this species were collected in Habu Port and Tomiura in May and June 2016. The larvae of *P. tsubaki* are similar to those of *Pseudopolydora pulchra* (Carazzi, 1893) in having ramified melanophores covering the ventral side; however, these cover only the central part of the body in the former species, whereas those of latter species cover the ventral surface almost entirely (Hannerz 1956, as *Polydora pulchra*; Rullier 1963, as *Polydora pulchra*). The dorsal pigment pattern is also different in these two species: two pairs of melanophores are distinct in *P. pulchra*, whereas the melanophore pair is ambiguous in *P. tsubaki*.

##### Pseudopolydora

Taxon classificationAnimaliaSpionidaSpionidae

sp.

6E8A1177-1E32-582F-8E33-D4BF4457332D

Fig. 9I

###### Larval morphology.

Overall larval shape slightly fusiform, head region enlarged due to broad prostomium and expanded lateral lips of vestibule. Prostomium rounded anteriorly. Three pairs of black eyes present, comprising one pair of rounded median eyes, one pair of large lateral eyes, and one pair of large anterior eyes. Lateral and anterior pairs of eyes link with each other and form a dumbbell‐shape almost divided into two equal parts by a deep constriction. Small mid‐dorsal melanophore present on chaetiger I. A distinct paired melanophore occurs dorso‐laterally from chaetiger II onwards, ramified in anterior chaetigers. Black pigment spots on sides of peristomium absent. Dot‐like black pigmentation on pygidium.

###### Remarks.

No benthic adult stages were collected in the present study. The larvae formed a monophyletic clade with the other *Pseudopolydora* species with > 50% bootstrap support (Figs 2, 3). Therefore, this species was identified as a member of *Pseudopolydora*. As the 18S rRNA gene sequences obtained from the larvae did not match any of the available *Pseudopolydora* sequences, this species is referred to *Pseudopolydora* sp.

Only one larva individual was collected from Sasuhama in August 2011. The dorsal pigment pattern of this larva somewhat resembles that of *P. paucibranchiata*; however, the mid‐dorsal pigment of this species is weaker and its dorsolateral melanophores are more ramified than those of *P. paucibranchiata*. The eye arrangement of this larva resembles that of late *P. tsubaki* larvae: three pairs of black eyes are present, but not in a straight line.

##### Genus Spio

Taxon classificationAnimaliaSpionidaSpionidae

Fabricius, 1785

283E6785-5962-5122-83A6-C00DD7E311E5

###### Larval diagnosis.

Overall body shape long, slender, and weakly or moderately fusiform. Prostomium small and rounded anteriorly. Lateral part of peristomium weakly demarcated from prostomium. Three pairs of black eyes present, most lateral often double‐eyes. Dorsal pigmentation consists of transverse band‐shaped or dot‐like paired lateral melanophores. Some species lack black pigmentation. Ventral pigment usually absent. Dark‐brown pigment may be present on pygidium. Nototrochs occur in all chaetigers except first one or two chaetigers, where nuchal organs develop. Gastrotrochs occur regularly in every other chaetiger from chaetiger III onwards. Larval chaetae on first chaetiger usually fairly long. Branchiae develop in late larvae, first on chaetiger II or III. One pair of anal cirri present on pygidium in late larvae (Thorson 1946, as spionid larva C, E, and F; Hannerz 1956; Wu et al. 1965; Simon 1963, 1967, 1968; Guérin 1972; Srikrishnadhas and Ramamoorthi 1981; Plate and Husemann 1994).

#### Identification key to species of the larvae belonging to the genus *Spio* in northeastern Japan

**Table:** 

1	Two rows of black melanophore spots on each side of dorsum from chaetiger I onwards, linking by band‐shaped medial black pigmentation from chaetiger IV or V	***Spio* sp. 2**
–	Dorsal black melanophores not distinct; rows of faint transverse band‐shaped black pigmentation present on dorsum from chaetiger IV onwards	***Spio* sp. 1**

##### Spio

Taxon classificationAnimaliaSpionidaSpionidae

sp. 1

1AD75CAF-2760-535E-8A66-00177F5E01F6

Fig. 9J, K

###### Larval morphology.

Overall larval shape slender and weakly fusiform. Larval chaetae on first chaetiger fairly long. Prostomium round anteriorly. Small patches of black pigment on peristomium ventrally. Three pairs of black eyes present, most lateral pairs double‐eyes. Distinct black melanophore absent, rows of faint transverse band‐shaped black pigmentation on dorsum from chaetiger IV onwards. Pharynx exhibits weak dark or brownish pigmentation. Gut yellow‐green in color due to ingested food.

###### Remarks.

Adult individuals of this species were collected from Rishiri Island, northern Japan, in July and August 2017. These specimens were previously identified as *S. arndti* Meißner, Bick & Bastrop, 2011 (Abe et al. 2019c) since adult morphology agreed. Although 18S rRNA gene sequence obtained in the present study 100% match with that of *S. arndti* (FR823434, 1761/1761 bp), because the 16S rRNA gene sequences were different (6.7%, 30/451 bp), the species reported here is referred to *Spio* sp. 1. The 16S rRNA gene sequence of *Spio* sp. 1 was rather more similar (96.1%, 298/310 bp) to that of *Spio* sp. 2573 from Russia (KT200126), but conspecificity of these two is unclear. A few planktonic larvae of this species were collected from Onagawa Bay only in May 2011. The larvae and adults were confirmed to match (18S: 1762/1762, 16S: 466/467 bp) using molecular data (Fig. 2).

The absence of distinct black melanophores in larvae of this species differentiates them from those of *Spio* sp. 2 (see below). Slight dorsal pigmentation was also reported in adelphophagic benthic larvae of *Spio setosa* Verrill, 1873 sensu Simon (1967, 1968), which were essentially unpigmented, and in those of *Spio multioculata* (Rioja, 1918) described by Hannerz (1956). However, the larval morphologies of these two species are different from that of *Spio* sp. 1 in lacking ventral black pigment on the peristomium (in both former species) and long larval chaetae on the first chaetiger (in *S. setosa*), and in having a relatively thickened body shape (in both species).

##### Spio

Taxon classificationAnimaliaSpionidaSpionidae

sp. 2

D13BF9AA-B56A-5A53-93A0-AC65B8F0D346

Fig. 9L–N

###### Larval morphology.

Overall larval shape elongated, slender, weakly fusiform. Larval chaetae on first chaetiger fairly long. Prostomium round anteriorly. Small patches of black pigment on lateral peristomium present ventrally. Three pairs of black eyes present, most lateral pairs double‐eyes. Two rows of dot‐like black melanophores on each side of dorsum from chaetiger I onwards, linking by band‐shaped medial black pigmentation from chaetiger IV or V. Pharynx exhibits weak dark or brownish pigmentation. The larvae which are ready to metamorphose have branchiae from chaetiger II, pigment spot on palps, and a pygidium with four leaf-shaped anal cirri.

###### Remarks.

Adult individuals were collected from muddy sand sediments of shallow water in Sasuhama in September 2011. These adults were morphologically identified as a *Spio* species, but they could not be identified to species level. *Spio* spp. 1 and 2 are distinguishable morphologically by the number of ventral epidermal glands. The 18S and 16S rRNA gene sequences obtained in the present study did not match any of the available *Spio* sequences (Figs 2, 3). The larvae and adults were confirmed to 100% match using molecular data (Fig. 2).

Planktonic larvae of this species were found in Sasuhama and Onagawa Bay from April to August during the study period. Larval morphology and pigmentation pattern of this species is similar to that of *Spio decorata* Bobretzky, 1870 described by Guérin 1972. However, the latter species was originally described from the Black Sea and has not been recorded in Japan.

## Discussion

### Larval identification based on the molecular data

The present study identified 41 species from 14 genera of planktonic spionid larvae by comparing adult and larval gene sequences and revealed high diversity of spionid larvae in neritic plankton communities (Table 1, Figs 2, 3). Planktonic spionid larvae of several species could not be identified to species level because of the lack of adult reference sequences or difficulties in adult identification. As the genetic information available for many marine invertebrate taxa including polychaetes is insufficient, the increase in gene sequence data based on accurate species identification and the establishment of a comprehensive database of adult reference sequences are essential for a more precise and efficient larval molecular identification. However, most of the larvae from the present study that did not have sequences that matched those of adults were identified to genus level based on their position within the phylogenetic tree; this was only possible because many of the spionid genera were recovered well or moderately supported monophyletic groups in our molecular phylogenetic analyses (Figs 2, 3). In contrast, the monophyly of some spionid taxa, particularly of the genera *Dipolydora*, *Malacoceros*, and *Prionospio* were ambiguous and not well supported in the phylogenetic tree recovered herein. It should be noted that *Malacoceros indicus* and Malacoceros cf. indicus were recovered as quite distant from *Malacoceros fuliginosus* and *Malacoceros* sp. (Fig. 3), potentially indicating the paraphyletic origins of these two clades. The results of the phylogenetic analyses also showed that the monophyly of subfamily Nerininae is doubtful and more likely to be paraphyletic. Because intergeneric phylogenetic relationships were ambiguous due to the low statistical support of most of the higher internal nodes (Figs 2, 3), it was difficult to compare with the previous results of phylogenetic relationships among spionid genera provided by Sigvaldadóttir et al. (1997) and Blake and Arnofsky (1999). The results of our phylogenetic analyses reinforce the need for a more robust and comprehensive molecular phylogenetic study of this taxon to test the monophyly of each genus and subfamily and to shed light on the phylogenetic relationships among spionid genera.

In the present study, many spionid species were collected as planktonic larval stages. This emphasizes the effectiveness of field investigations of both larval and adult stages to assess the cryptic species diversity in benthic invertebrate fauna of coastal waters. The reference gene sequences used in the present study for adults covered most of the species belonging to the genera *Polydora* and *Pseudopolydora* hitherto recorded from Japan (Sato‐Okoshi 1999, 2000; Sato‐Okoshi and Abe 2012, 2013; Teramoto et al. 2013; Abe et al. 2016; Simon et al. 2019a). However, the sequences of some *Polydora* and *Pseudopolydora* larvae, namely *Polydora* sp. 2, *Polydora* sp. 3, and *Pseudopolydora* sp., did not match any adult reference sequences. This emphasizes the need for detailed taxonomic studies with a more comprehensive sampling of spionid adults to reveal the actual biological diversity of this taxon in Japan.

### Morphology of spionid larvae

The family Spionidae can be divided into two subfamilies: 1) Spioninae Söderström, 1920, which includes the genera *Spio*, *Microspio* Mesnil, 1896, *Pygospio* Claparède, 1863, and genera of the tribe Polydorini; and 2) Nerininae Söderström, 1920, which includes almost all remaining spionid genera, except for *Atherospio* Mackie & Duff, 1986 and *Pygospiopsis* Blake, 1983 (Blake 2006), besides *Poecilochaetus* and *Trochochaeta*, which were recently placed within the family Spionidae (Radashevsky et al. 2018). The larvae of these two subfamilies were distinguished in the present study based on color and number of eyes, body pigmentation, shape of peristomium, and distribution of gastrotrochs, and by the following characteristics identified by Hannerz (1956): larvae of Spioninae have three pairs of black eyes (lateral eyes are often double eyes), distinct black pigmentation with melanophores, lateral parts of the peristomium not demarcated from prostomium, and gastrotrochs present from chaetiger III, V, or VII onwards, but absent in all of the succeeding chaetigers (Figs 7–9); larvae of Nerininae have two pairs of red or dark red eyes, lack distinct black pigmentation, lateral parts of the peristomium are well developed and often demarcated from prostomium, and gastrotrochs present from chaetiger II or III onwards and in all succeeding chaetigers (Figs 4, 5). Blake (1969) also discussed the presence of ventral ciliary patches in early larval stages as a common characteristic of subfamily Spioninae, but these cilia were not herein observed because they are lost in early larval stages.

Hannerz (1956) reported the following exceptions to the abovementioned typical larval morphologies: larvae of *Prionospio fallax* Söderström, 1920 (as *P. malmgreni*, see Blake and Arnofsky 1999) with two pairs of black eyes; larvae of *Malacoceros* (as *Scolelepis*), which belongs to Nerininae, with intermediate characteristics between the two subfamilies, i.e., with three pairs of black eyes and gastrotrochs regularly distributed on every other chaetiger as in Spioninae larvae. However, Plate and Husemann (1994) reported that the larvae of *Malacoceros fuliginosus* (Claparède, 1868) have up to three pairs of red eyes in early stages and that eye color changes to black as larvae develop. Radashevsky and Migotto (2006) reported that the larvae of *Malacoceros* sp. have two pairs of red eyes. In the present study, the larvae of *Rhynchospio* have two pairs of dark red eyes (Fig. 4F, G), whereas their morphology resembled those of *Malacoceros* species described by Hannerz (1956). Radashevsky (2007) also reported that larvae of *Rhynchospio nhatrangi* have two pairs of red eyes. Besides the various reports on the number and color of eyes, the close relationships of *Malacoceros* and *Rhynchospio* to the subfamily Spioninae were indicated by larval morphology, and results of the phylogenetic analyses presented (Figs 2, 3) also provide some support for this hypothesis.

In the subfamily Spioninae, the most obvious larval differences between genera and species are the overall body shape and type and arrangement of pigmentation (Blake and Arnofsky 1999). The overall body shape of larvae of *Polydora*, *Dipolydora*, and *Spio* tended to be long and slender, whereas those of *Boccardiella*, *Boccardia*, and *Pseudopolydora* tended to be thick and fusiform (Figs 7–9), although *Boccardia proboscidea* (Fig. 7A, B) and *Boccardia* sp. 1 (Fig. 7D–F) showed relatively slender body shapes. The lateral enlargement of the prostomium in Spioninae is variable: large in *Boccardiella* and *Pseudopolydora*, moderate in *Polydora* and *Boccardia*, and small in *Dipolydora* and *Spio*. Fairly long larval chaetae on the first chaetiger are highly characteristic of *Spio* within Spioninae.

The dorsal black pigmentation with melanophores is distinct in the subfamily Spioninae, and the pattern of rows of melanophores is generally diagnostic among Spioninae genera. The typical patterns of dorsal pigmentation rows in larvae are as follows: a pair of transverse bands of black pigment on some anterior chaetigers followed by a pair of large branching melanophores in *Polydora*; lack of large melanophores, but with a pair of medial spots or bands, a pair of lateral pigment patches, and mid‐dorsal black pigment spot continuing posteriorly from the anterior chaetigers in *Dipolydora*; mid‐dorsal melanophores arranged in a single row in *Boccardia*; medial and lateral pairs of spots or bands with black pigmentation and a small patch of pigment at the base of the notopodia present on almost all chaetigers in *Boccardiella*; a mid‐dorsal melanophore on the first chaetiger, and one or two pairs of melanophores on each chaetiger in *Pseudopolydora*; a pair of black pigment spots and transverse black pigment bands linking them on each chaetiger in *Spio* (Figs 7–9). These typical dorsal pigment patterns were also reported in many previous studies (Hannerz 1956; Blake 1969, 2006; Blake and Arnofsky 1999; and references cited therein). However, unusual larval pigment patterns are often found in members of each of the aforementioned genera; therefore, these typical larval pigment patterns are not wholly consistent within each genus. For example, the single row of dorsal melanophores typical of *Boccardia* larvae was also observed in larvae of Polydora cf. glycymerica (Fig. 8E) and Dipolydora cf. commensalis (Fig. 7L), and have been reported in *Polydora glycymerica* (Radashevsky 1989), *Polydora hermaphroditica* (Hannerz 1956; Plate and Husemann 1994), *Dipolydora commensalis* (as *Polydora commensalis*: Andrews 1891; Hatfield 1965; Blake 1969; Radashevsky 1989), and *Pseudopolydora rosebelae* Radashevsky & Migotto, 2009. In contrast, the single row of dorsal melanophores is absent in *Boccardia chilensis* Blake & Woodwick, 1971 (Carrasco 1976; Blake and Kudenov 1981), *Boccardia pseudonatrix* (Fig. 7C), and *Boccardia semibranchiata* Guérin, 1990 (Guérin 1991). The larvae of Pseudopolydora cf. kempi lack a mid‐dorsal melanophore on the first chaetiger, which is typical in *Pseudopolydora* larvae (Fig. 9C; Kondoh et al. 2017). The distinct dorsal black pigment is absent in *Spio setosa* Verrill, 1873 (Simon 1967, 1968) and *Spio* sp.1 (Fig. 9J, K).

Larvae of the following Spioninae genera were not collected in the present study: *Microspio*, *Pygospio*, and the polydorid genera *Amphipolydora* Blake, 1983, *Carazziella* Blake & Kudenov, 1978, *Polydorella* Augener, 1914, and *Tripolydora* Woodwick, 1964 (among them, *Microspio* and *Carazziella* have records from Japan by Okuda 1937, Sato-Okoshi 1998). Little is known about the larval morphology of the genera *Amphipolydora*, *Polydorella*, and *Tripolydora*. The larvae of *Microspio* resemble those of *Spio* in having a long and slender body shape and band‐shaped dorsal black pigmentation (e.g., Hannerz 1956; Cazaux 1971). The larvae of *Carazziella* resemble those of the polydorid genus *Boccardia* in having a fusiform body shape and a single row of dorsal melanophores (Carrasco 1976, as *Polydora citrona*; Blake and Arnofsky 1999; Blake 2006). The morphology of planktonic larval stages of *Pygospio elegans* as described in Hannerz (1956) resembles that of *Pseudopolydora* in having a thick and fusiform body shape, laterally enlarged prostomium, and mid‐dorsal melanophore on the first chaetiger. Blake (1969) also noted the morphological similarity between the larvae of *Pseudopolydora* and *Pygospio elegans* and suggested the possibility that polydorids are closely related to *Pygospio* through *Pseudopolydora*. Subsequently, Blake and Woodwick (1975) reported the similarities of nurse egg feeding patterns between *Pseudopolydora kempi* and *Pygospio elegans*, further strengthening the view of a close relationship between these two genera. This hypothesis is supported by the results of the phylogenetic analysis presented, showing that polydorids plus *Pygospio* form a monophyletic clade with robust statistical supports (Fig. 3).

In the subfamily Nerininae, as in Spioninae, the most obvious differences among genera are also regarding their overall body shapes. The lateral parts of the peristomium are conspicuous, well developed, and distinctly demarcated from the prostomium in larvae of *Laonice*, *Rhynchospio*, and *Scolelepis*, but they are less pronounced in those of *Aonides*, *Paraprionospio*, *Prionospio*, and *Spiophanes*, as previously noted by Hannerz (1956). Larvae of the former group of genera (*Laonice*, *Rhynchospio*, *Scolelepis*) also have a relatively wide body shape, whereas those of the latter group have a narrow body shape. Regarding the larvae of the former group, the prostomium is more or less stumpy and not pointed anteriorly in *Rhynchospio* and *Laonice*; however, *Scolelepis* larvae have a unique body shape distinct from other spionid genera and their prostomium is pointed anteriorly, terminating in a tapered tip, and the lateral parts of the peristomium are demarcated and bearing a large peristomial umbrella. The larvae from the latter group (*Aonides*, *Paraprionospio*, *Prionospio*, *Spiophanes*), *Paraprionospio*, and *Prionospio* characteristically have extremely long and thin bodies with numerous chaetigers. In particular, larvae of *Paraprionospio* are extremely large in terms of body size and chaetiger number at metamorphosis among the spionid larvae (Yokoyama 1981). The larval morphology of the genus *Poecilochaetus* resembles that of *Paraprionospio* and *Prionospio*: larvae have extremely long and slender transparent bodies without distinct black pigmentation. However, the first differs from the other two in having small lateral pigment spots on each side of the chaetigers, a long metatrochophore stage (up to ca. 30–40 chaetiger stages), and a serpentine swimming behavior with developed parapodia bearing cirriform dorsal and ventral postchaetal lobes in the nectosoma stage. *Poecilochaetus* larvae are distinctive among spionid larvae in having gastrotrochs from chaetiger I onwards despite all other Nerininae larvae having gastrotrochs from chaetiger II or III onwards. In the present study, a pair of lateral processes on the prostomium developed in late larvae was found only in larvae of *Rhynchospio* and *Spiophanes*, it also previously described for *Malacoceros* larvae (Hannerz 1956). Fairly long and straight larval chaetae on the first chaetiger are highly characteristic of *Aonides* larvae, especially in the early stages (Fig. 4A, B); however, similar long and straight larval chaetae were also herein observed in larvae of *Spio* sp. 2 (Fig. 9L, M). Although larvae of the genera *Malacoceros*, *Marenzelleria* Mesnil, 1896, *Streblospio* Webster, 1879, and *Trochochaeta* (subfamily Nerininae) were not collected in the present study, larval morphologies of these genera have been well described in previous studies. The larvae of *Malacoceros* resemble those of *Rhynchospio* (see above). The larvae of *Marenzelleria* (Bochert and Bick 1995) resemble those of *Laonice* in having the following characters: the remains of the egg membrane visible in early stages; in late larvae, lateral parts of the peristomium are conspicuous, well developed and distinctly demarcated from prostomium, and palps start developing laterally on the peristomium; the body is broader than the prototroch; both notopodial and neuropodial larval chaetae are present. However, the larvae of these two genera differ in the arrangement of nototrochs and gastrotrochs. The larval morphology of the genus *Streblospio*, which is included in the *Prionospio* complex (Dean 1965; Blake and Arnofsky 1999; Blake 2006) resembles that of *Prionospio*. The larval morphology of the genus *Trochochaeta* is distinctive among spionid larvae in having unusually long larval chaetae on the first chaetiger, very pronounced peristomial umbrella with two rows of robustly developed prototrochs, and total absence of nototrochs; larvae of this genus also present the typical morphological characteristics of Nerininae larvae, such as two pairs of red eyes, lack of distinct black pigmentation, and gastrotrochs from chaetiger II onwards on all succeeding chaetigers, although gastrotrochs of *Trochochaeta* larvae are weakly developed and those on chaetiger II are especially small and inconspicuous in late‐stage larvae (Hannerz 1956, as *Disoma*; Blake and Arnofsky 1999; Blake 2006).

There is insufficient information on the larval morphology of the remaining genera of Nerininae. The larval development and morphology of *Dispio uncinata* Hartman, 1951 were described, and this species’ close relationship with *Aonides* was suggested by Blake and Arnofsky (1999) and Blake (2006). However, Radashevsky et al. (2011) pointed out that the larvae described by these authors are most likely those of *Aonides californiensis* Rioja, 1947 rather than of a *Dispio* Hartman, 1951 species. Species‐level identification of larvae from this subfamily is generally more difficult because of the lack of structured pigmentation, which is a useful characteristic for identifying species of Spioninae larvae. Especially in the genus *Prionospio*, larval morphology is quite simple and similar among species, which made it impossible to find morphological characters to distinguish between them in the present study.

Except for *Prionospio* spp., most of the planktonic spionid larvae collected in the present study have morphological characteristics that could be used to distinguish genera and species, and allowed morphological identification based on overall body shape and pigment patterns. The present paper provides identification keys to genera and species of planktonic spionid larvae from northeastern Japan; however, sufficient attention to developmental and/or intraspecific variation of larval morphological characteristics and the disappearance of pigments after fixation (only the black pigment usually remains after fixation) is required for accurate larval identification.

## Supplementary Material

XML Treatment for Aonides

XML Treatment for Aonides aff. oxycephala

XML Treatment for Laonice

XML Treatment for Laonice

XML Treatment for Laonice

XML Treatment for Paraprionospio

XML Treatment for Paraprionospio coora

XML Treatment for Poecilochaetus

XML Treatment for Poecilochaetus

XML Treatment for Prionospio

XML Treatment for Prionospio krusadensis

XML Treatment for Prionospio membranacea

XML Treatment for Prionospio

XML Treatment for Rhynchospio

XML Treatment for Rhynchospio aff. asiatica

XML Treatment for Scolelepis

XML Treatment for Scolelepis cf. kudenovi

XML Treatment for Scolelepis

XML Treatment for Scolelepis

XML Treatment for Spiophanes

XML Treatment for Spiophanes uschakowi

XML Treatment for Spiophanes aff. uschakowi

XML Treatment for Boccardia

XML Treatment for Boccardia proboscidea

XML Treatment for Boccardia pseudonatrix

XML Treatment for Boccardia

XML Treatment for Boccardia

XML Treatment for Boccardiella

XML Treatment for Boccardiella hamata

XML Treatment for Dipolydora

XML Treatment for Dipolydora bidentata

XML Treatment for Dipolydora cf. commensalis

XML Treatment for Dipolydora giardi

XML Treatment for Dipolydora cf. socialis

XML Treatment for Dipolydora

XML Treatment for Polydora

XML Treatment for Polydora brevipalpa

XML Treatment for Polydora cornuta

XML Treatment for Polydora cf. glycymerica

XML Treatment for Polydora hoplura

XML Treatment for Polydora onagawaensis

XML Treatment for Polydora cf. spongicola

XML Treatment for Polydora

XML Treatment for Polydora

XML Treatment for Polydora

XML Treatment for Pseudopolydora

XML Treatment for Pseudopolydora aff. achaeta

XML Treatment for Pseudopolydora cf. kempi

XML Treatment for Pseudopolydora paucibranchiata

XML Treatment for Pseudopolydora cf. reticulata

XML Treatment for Pseudopolydora tsubaki

XML Treatment for Pseudopolydora

XML Treatment for Spio

XML Treatment for Spio

XML Treatment for Spio
